# Two-year outcomes after early postnatal high-dose fat-soluble enteral vitamin A supplementation in extremely low birth weight infants: follow-up of the NeoVitaA randomized controlled trial

**DOI:** 10.1016/j.eclinm.2025.103495

**Published:** 2025-09-15

**Authors:** Martin Poryo, Ludwig Gortner, Johannes Bay, Axel R. Franz, Harald Ehrhardt, Lars Klein, Judith Behnke, Tina Frodermann, Jutta Petzinger, Christoph Binder, Susanne Kirschenhofer, Anja Stein, Britta Hüning, Axel Heep, Eva Cloppenburg, Julia Muyimbwa, Torsten Ott, Julia Sandkötter, Norbert Teig, Susanne Wiegand, Michael Schroth, Andrea Kick, Donald Wurm, Corinna Gebauer, Knud Linnemann, Jochen Kittel, Christian Wieg, Ursula Kiechl-Kohlendorfer, Susanne Schmidt, Ralf Böttger, Wolfgang Thomas, Francisco Brevis Nunez, Antje Stockmann, Thomas Kriebel, Andreas Müller, Daniel Klotz, Patrick Morhart, Donatus Nohr, Hans Konrad Biesalski, Eleni Z. Giannopoulou, Susanne Hilt, Stefan Wagenpfeil, Nadja Haiden, Matthew Rysavy, Christoph Härtel, Christian Ruckes, Anne Ehrlich, Sascha Meyer

**Affiliations:** a Saarland University Medical Center, Department of Paediatric Cardiology, Kirrberger Straße, Building 9, 66421, Homburg, Saar, Germany; b Saarland University Medical Center, Department of Paediatrics and Neonatology, Kirrberger Straße, Building 9, 66421, Homburg, Saar, Germany; c University Hospital Tübingen, Department of Neonatology and Center for Paediatric Clinical Studies, Calwerstraße 7, 72076, Tübingen, Germany; d University Medical Center Ulm, Department of Paediatrics and Adolescent Medicine, Division of Neonatology and Pediatric Intensive Care Medicine, Eythstrasse 24, 89075 Ulm, Germany and Justus-Liebig-Universität, Department of General Paediatrics and Neonatology, Feulgenstr.12, 35392, Gießen, Germany; e University Hospital Giessen, Department of General Paediatrics and Neonatology, Feulgenstr.12, 35392, Gießen, Germany; f Medical University Wien, Department of Neonatology, Paediatric Intensive Care and Neuropaediatrics, Währinger Gürtel 18-20, 1090, Wien, Austria; g University Hospital Essen, Clinic for Paediatrics I, Department of Neonatology, Hufelandstraße 55, 45147, Essen, Germany; h Clinical Centre Oldenburg, Department of Neonatology, Paediatric Intensive Care, Paediatric Cardiology, Paediatric Pneumonology and Allergology, Rahel-Straus-Straße 10, 26133, Oldenburg, Germany; i Medi Kids, Medical Centre for Children & Adolescents, Mareinstraße 11, 49377, Vechta, Germany; j University Hospital Münster, Department of Neonatology, Albert-Schweitzer-Campus 1, Building A1, 48149, Münster, Germany; k University Hospital Bochum, St. Josef-Hospital, Department of Neonatology and Paediatric Intensive Care, 44791, Bochum, Germany; l Cnopf'sche Kinderklinik, Department of Neonatology and Paediatric Intensive Care, St.-Johannis-Mühlgasse 19, 90419, Nürnberg, Germany; m Marienhaus Klinikum St. Elisabeth Saarlouis, Department of Paediatrics, Kapuzinerstraße 4, 66740, Saarlouis, Germany; n University Hospital Leipzig, Department of Neonatology, Liebigstraße 20a, Building 6, 04103, Leipzig, Germany; o University Hospital Greifswald, Department of Neonatology and Paediatric Intensiv Care, Ferdinand-Sauerbruch-Straße, 17475, Greifswald, Germany; p University Hospital Regensburg, University Children's Hospital Regensburg (KUNOClinics), Clinic St. Hedwig, Steinmetzstr. 1-3, 93049, Regensburg, Germany; q Clinical Centre Aschaffenburg-Alzenau, Department of Neonatology and Paediatric Intensive Care, Am Hasenkopf 1, 63739, Aschaffenburg, Germany; r Medical University Innsbruck, Department of Paediatrics II, Neonatology, Anichstraße 35, 6020, Innsbruck, Austria; s Ludwig-Maximilians-University Munich, Dr. von Haunersches Kinderspital, Department of Neonatology, Lindwurmstrasse 4, 80337, München, Germany; t University Hospital Magdeburg, Department of Neonatology, Leipziger Straße 44, 39120, Magdeburg, Germany; u Hospital Mutterhaus der Borromäerinnen, Deparment of Paediatrics, Feldstraße 16, 54290, Trier, Germany; v Sana Hospital Duisburg, Department of Neonatology and Paediatric Intensive Care, Zu den Rehwiesen 9, 47055, Duisburg, Germany; w Evangelical Hospital Oberhausen, Center of Paediatrics, Department of Neonatology, Virchowstraße 20, 46047, Oberhausen, Germany; x Westpfalz-Klinikum Kaiserslautern, Department of Paediatrics and Adolescents, Hellmut-Hartert-Strasse 1, 67655, Kaiserslautern, Germany; y University Hospital Bonn, Eltern-Kind-Zentrum (ELKI), Department of Neonatology and Paediatric Intensive Care, Building 30, Venusberg-Campus 1, 53127, Bonn, Germany; z Bethel Center for Pediatrics, Department of Neonatology and Pediatric Intensive Care Medicine, University Hospital OWL, University of Bielefeld, Grenzweg 10, 33617, Bielefeld, Germany; aa University Hospital Erlangen, Department of Neonatology and Paediatric Intensive Care, Loschgestraße 15, 91054, Erlangen, Germany; ab University Hohenheim, Department of Molecular Nutrition Sciences, Schloss Hohenheim 1, 70599 Stuttgart, Germany; ac University Hospital Ulm, Department of Paediatric Endocrinology and Diabetology, Eythstraße 24, 89075, Ulm, Germany; ad Saarland University, Department of Medical Biometrics, Epidemiology and Medical Informatics, Building 86, 66421, Homburg, Germany; ae Kepler University Hospital, Department of Neonatology, Krankenhausstraße 26-30, 4020, Linz, Austria; af UTHealth Houston McGovern Medical School, Pediatrics at McGovern Medical School, Houston, TX, USA; ag University Hospital Würzburg, Department of Pediatrics, Josef-Schneider Straße 2, 97080, Würzburg, Germany; ah Universitätsmedizin der Johannes Gutenberg-Universität Mainz, Interdisziplinäres Zentrum Klinische Studien (IZKS), Langenbeckstraße 1, 55131, Mainz, Germany; ai Städtisches Klinikum Karlsruhe, Department of Paediatrics, Moltkestraße 90, 76133, Karlsruhe, Germany

**Keywords:** Bayley-II, Bayley-III, Bronchopulmonary dysplasia, Death, Extremely low birth weight infants, Neurodevelopmental outcome, Respiratory outcome, Vitamin A

## Abstract

**Background:**

The longer-term effects of early high-dose vitamin A to support lung development in preterm infants remain to be clarified. The aim of the NeoVitaA follow-up study was to assess the effects of early postnatal additional high-dose fat-soluble enteral vitamin A supplementation (HD-VitA) vs. placebo (control) for 28 days on respiratory complications and neurodevelopmental outcome in ELBW infants receiving recommended basic vitamin A supplementation, specified as secondary outcome parameters in the NeoVitaA trial.

**Methods:**

The trial was approved by the ethics committee of Saarland, Saarbruecken, Germany (file number: 70/2011) as well as by all local ethics committees and the Bundesinstitut für Arzneimittel und Medizinprodukte (BfArM, Bonn, Germany). The NeoVitaA trial was registered with EudraCT (2013-001998-24) and DRKS (DRKS00006541). This follow-up covers secondary endpoints at 12 and 24 months as mentioned in the registration. Follow-up took place between September 2019 and June 2024. Follow-up assessment at 12 and 24 months’ corrected age (CA) of infants enrolled in the NeoVitaA-trial included anthropometric data, number of antibiotic treatments, antibiotic treatments for pulmonary infections, hospital admissions, and hospital admissions for pulmonary infections, composite scores of the Bayley Scale of Infant and Toddler Development, second (Bayley-II) or third edition (Baley-III), other medical diagnoses and medical treatments.

**Findings:**

Follow-up examinations were available for 759 infants (83.0%). HD-VitA had no effect on number of antibiotics needed for pulmonary infections or number of hospital admissions for pulmonary infections at 12 or 24 months' CA. At 24 months’ CA, the median number of antibiotic courses for pulmonary infections was one for both the HD-VitA and control group; the median number of hospital admissions for pulmonary infections per patient was 0 (HD-VitA) and 1 (control).

Successful Bayley assessment was completed in 618/759 infants (92 Bayley-II, 526 Bayley-III). The median Mental Development Index score for Bayley-II was 95 vs. 97 (median difference −5.0, 95%-CI [−12.0, 2.0]) and Psychomotor Development Index 96 vs. 92 (median difference 3.0, 95%-CI [−4.0, 8.0]), with intervention and placebo, respectively. The median cognitive composite score for Bayley-III was 95 vs. 95 (median difference 0.0, 95%-CI [−5.0, 0.0]) and motor score was 92 vs. 92 (median difference 0.0, 95%-CI [−4.0, 3.0]), respectively.

**Interpretation:**

Early postnatal high-dose enteral fat-soluble vitamin A supplementation in ELBW infants did not affect pulmonary or developmental outcomes at 24 months’ CA.

**Funding:**

The NeoVitaA trial was funded by the 10.13039/501100001659Deutsche Forschungsgemeinschaft ME 3827/1-1/2 and European Clinical Research Infrastructures Network.

**Research in context**

**Evidence before this study**

Bronchopulmonary dysplasia (BPD) affects as many as 45% of extremely premature and extremely low birth weight infants (ELBW; birth weight <1000 g). The disease is associated with mortality and long-term morbidity, including cerebral palsy and neurodevelopmental impairments. BPD has lifelong implications for adult health and quality of life entailing major health care resource utilisation.

Few therapeutic approaches in ELBW infants have proven safe or effective to reduce BPD. Vitamin A plays an integral role in lung growth and differentiation. Intramuscular (i.m.) vitamin A supplementation injection has reduced death or BPD in a large randomised controlled trial and subsequent meta-analyses. However, findings regarding the effect of high-dose enteral vitamin A supplementation to reduce BPD or death in the setting of recommended dietary enteral vitamin A supplementation have been inconclusive. The NeoVitaA RCT–the largest multicentre trial investigating high-dose enteral fat-soluble vitamin A supplementation compared to recommended dietary vitamin A supplementation–showed no benefit for the primary outcome BPD or death. However, data on respiratory morbidity and child development at 12 and 24 months’ corrected gestational age have not been previously described.

Prior to study initiation, we performed searches of PubMed, Scopus, Web of Science, MEDLINE, and Cochrane Library (CENTRAL), focusing on research articles published between 1990 and 2013. After study implementation, the time period was extended to the year 2025.

**Added value of this study**

The NeoVitaA trial did not demonstrate the superiority of high-dose enteral fat-soluble vitamin A supplementation compared to recommended dietary vitamin A supplementation for important respiratory and neurodevelopmental outcome parameters at 12 and 24 months’ corrected age.

**Implications of all the available evidence**

Although high-dose enteral fat-soluble vitamin A may be considered safe, higher enteral dosages than those proposed in the guideline by the ESPGHAN do not appear to provide benefit for respiratory or neurodevelopmental outcomes in ELBW infants. Alternative routes or formulations of vitamin A drug administration (e.g., by inhalation) may be worth exploring. Furthermore, recently increased routine dietary vitamin A supplementation, contemporary implementation of less-invasive ventilation and other interventions may alter the potential impact of high-dose vitamin A compared to earlier studies.

Our study demonstrated an absence of difference in respiratory outcomes (e.g., number of pulmonary infections) between the intervention and the control group at 12 and 24 months’ corrected age that is consistent with the absence of difference in the incidence of BPD during initial hospitalization. Moreover, the use of early high-dose enteral fat-soluble vitamin A supplementation did not impact the neurodevelopmental outcome at 24 months corrected age.

## Introduction

Improvements in perinatal care have increased survival in infants born extremely premature (EPT, <28 weeks of gestational age) and at extremely low birthweight (ELBW, <1000 g). Bronchopulmonary dysplasia (BPD) affects as many as 45% of EPT and ELBW infants. It is characterized by respiratory injury and alternations of development that persist into childhood and adulthood. BPD results in increased healthcare utilization during the first few years of life and is associated with cerebral palsy, neurodevelopmental impairment (NDI) and behavioral problems.^1^, ^2^, ^3^, ^4^, ^5^

Since the first description of BPD in the 1960s, both infants survive at lower gestations and the nature of intensive care provided to them has changed. Many therapies aiming to prevent or mitigate the severity of BPD and associated long-term sequelae in ELBW infants have proven ineffective (e.g., inhaled nitric oxide), or have significant adverse effects (e.g., postnatal corticosteroids).^6^, ^7^, ^8^, ^9^ Vitamin A plays an integral role in lung growth and differentiation.^10^, ^11^, ^12^ Early studies indicate that the intramuscular administration of vitamin A can reduce death or BPD, and can induce a sustained rise in vitamin A blood levels.^13^, ^14^, ^15^, ^16^ Prior studies of vitamin A treatment showed no differences on NDI at 18–22 months’ corrected age (CA), but were not powered to adequately evaluate this question.^14^^,^^17^^,^^18^ Notably, studies of high-dose vitamin A supplementation first appeared in the 1990s or earlier.^14^ Since this time, neonatal nutrition practices and respiratory management have advanced, leaving the relevance of high-dose vitamin A supplementation uncertain today. Contemporary dosages and routes of administering vitamin A to preterm infants vary considerably.^19^ Current European guidelines on enteral vitamin A supplementation in preterm infants recommend daily doses of 1333–3300 international units (IU)/kg.^20^

We reported our results of the NeoVitaA study in 2024, also showing that early postnatal additional high-dose enteral fat-soluble vitamin A supplementation in ELBW infants had no apparent adverse effects but failed to change the rate of moderate or severe BPD or death.^21^ The aim of the NeoVitaA follow-up study was to assess in ELBW infants receiving recommended basic enteral vitamin A supplementation of 1000 I.U./kg/day whether early postnatal additional high-dose enteral fat-soluble vitamin A supplementation (5000 I.U./kg body weight/day (HD-VitA)) vs. placebo for 28 days impacted respiratory morbidity or neurodevelopmental outcomes at 24 months’ CA.

## Methods

### Study design and patients

Details on the conduct of the NeoVitaA trial were previously reported.^21^ Briefly, this prospective, multicentre, randomised, stratified, parallel-group, double-blind, placebo-controlled, investigator-initiated, phase-III trial for superiority was conducted at 29 neonatal intensive care units (NICUs) in Austria (n = 2) and Germany (n = 27), and funded by the Deutsche Forschungsgemeinschaft (ME 3827/1-1/2) and European Clinical Research Infrastructures Network (ECRIN). The trial was approved by the ethics committee of Saarland, Saarbruecken, Germany (file number: 70/2011) as well as by all local ethics committees and the Bundesinstitut für Arzneimittel und Medizinprodukte (BfArM, Bonn, Germany).

The trial enrolled infants meeting all of these criteria: birth weight >400 and <1000 g, gestational age at birth ≤ 32+ ^0^ weeks’ postmenstrual age (PMA), need for mechanical ventilation, non-invasive respiratory support or supplemental oxygen (fraction of inspired oxygen (FiO_2_) > 0.21) within the first 72 postnatal hours, informed parental consent, at least minimal enteral feeds prior to administration of first dose of study medication, and planned outpatient follow-up at 1 and 2 years. Infants with severe congenital anomalies, suspected or proven non-bacterial infections, terminal illness as evidenced by pH < 7.0 for >2 h or persistent bradycardia (heart rate <100 beats per minute) associated with hypoxia for >2 h, or participation in another clinical drug trial (according to the German Medicinal Products Act), were excluded.

The primary endpoint was a composite outcome of moderate or severe bronchopulmonary dysplasia or death at 36+^0^ weeks PMA. Secondary endpoints were as follows: any bronchopulmonary dysplasia (all grades: mild, moderate, or severe); duration of any invasive positive pressure ventilation and duration of any positive pressure respiratory support; days of evolving bronchopulmonary dysplasia; necrotising enterocolitis of modified Bell stage IIA or greater; intraventricular haemorrhage of any grade; periventricular leukomalacia; retinopathy of prematurity of any grade; all-cause mortality; safety and tolerability of trial medication (daily clinical examinations, adverse events, laboratory data, serum retinol concentrations, and repeated cranial ultrasound on study days 7 [plus or minus 3 days], 14 [plus or minus 7 days], 21 [plus or minus 7 days], and 28 [plus or minus 7 days], and at PMA 36+ ^0^ weeks); serum retinol concentrations at birth, after study intervention, and at 36+ ^0^ weeks PMA; and anthropometric data (bodyweight, length, and head circumference at 36+ ^0^ weeks PMA). If home discharge occurred before 36+ ^0^ weeks PMA, all endpoints scheduled for assessment at 36+ ^0^ weeks PMA were assessed at discharge. In addition, secondary outcome parameters included respiratory and neurological asessment at 12 and 24 months CA as detailed below.

This follow-up covers secondary endpoints at 12 and 24 months as mentioned in the registration.

### Study intervention

The treatment between study groups differed only in the dose of supplemented vitamin A. The investigational medicinal product was fat-soluble vitamin A provided as Vitadral® (Aristo Pharma GmbH, Berlin, Germany) oral drops. The comparator trial medication was peanut oil (placebo oral drops), which is the vitamin A carrier in the aforementioned preparation. Following randomisation, subjects received either an additional provision of 5000 I.U./kg body weight/day of vitamin A supplementation (high-dose vitamin A (HD-VitA) group) or placebo (control group) enterally for 28 days. Both treatment groups received standard vitamin A supplementation of 1000 I.U./kg body weight/day throughout their study participation. A more detailed description of the study intervention is found in the original NeoVitaA trial.^21^

### Patient follow-up

Follow-up took place between September 2019 and June 2024. All surviving infants were invited for follow-up assessment at 12 and 24 months' CA according to German national guidelines.^22^ Assessment at follow-up included measurement of anthropometric data and thorough history taking about details regarding antibiotic treatment and rehospitalisation following initial discharge home after birth. At 24 months’ CA, the Bayley Scale of Infant and Toddler Development, second (Bayley-II) or third edition (Baley-III) were used to evaluate cognitive and motor development and the infant underwent a neurological exam.

### Neurodevelopmental outcomes

The follow-up visit was conducted by specially trained staff (neonatologists and/or neuropaediatricians) with experience in follow-up care for ELBW infants, including Bayley-II or Bayley-III as part of usual care.^23^^,^^24^ The Bayley-III was introduced in Germany in 2014 and, while 85.1% (n = 526/618) received the Bayley-III, the study period included some transition between Bayley-II and Bayley-III. The Bayley-II and Bayley-III include play tasks and questionnaires completed by parents/caregivers to assess their child's functioning level. For the cognitive, language, and motor scales, a composite score is calculated with a mean score of 100 (standard deviation (SD) ± 15). A composite score in any domain <85 (>−1 SD) indicated mild impairment requiring monitoring; and a composite score <70 (>−2 SD) indicated moderate impairment. Profound impairment was defined by a composite score <55 (>−3 SD).^25^^,^^26^

The Gross Motor Function Classication System (GMFCS) was used to classify patients with cerebral palsy identified during neurological examination.^27^ The GMFCS is scored a scale of 0 (no deficits) to 5 (most impaired).

### Randomisation and masking

Treatment allocation was done using a randomisation list generated at IZKS Mainz (Mainz, Germany) via SAS software (version 9.4, SAS Institute Inc. (2023), Cary, NC). Randomisation was stratified by trial site. Block randomisation with variable block sizes (two and four) was applied. A copy of the randomization list was provided to the University Medical Center pharmacy at Johannes Gutenberg University (Mainz, Germany; hereafter referred to as Mainz University Pharmacy) for packaging of double-blinded, numbered trial medication. Randomly assigned participants received the next lowest available medication number at the study centre. Multiple-birth infants were independently randomly assigned. Participants and all staff involved in the conduct or analysis of the trial remained masked to treatment until the database lock.

### Statistical analysis

Sample size calculation was based on a comparison of the primary composite outcome, with an expected event rate of 42% (event rate of moderate or severe BPD of 35% and an approximate mortality rate of 7%), taking into consideration birthweight, gestational age, and time interval until study inclusion.^4^^,^^28^ A 20% relative reduction (9% absolute reduction) in the primary composite outcome parameter was considered clinically relevant. Sample sizes of at least 457 in the control group and 457 in the experimental group were needed to achieve 80.9% power to detect an odds ratio (OR) of 0.68 (for proportions of 0.42 in the placebo group and 0.33 in the experimental treatment group), using a two-sided χ^2^ test with a global 0.05 significance level with one prespecified interim analysis after 50% of the patients were enrolled, considering the O'Brien-Fleming α-spending function. With the included 759 patients the follow-up still had 80% power to detected odds ratios of 0.57, 0.62, 0.65, or 0.66 when the reference probability amounts to 20%, 30%, 40%, or 50%.

Continuous data were analysed by Wilcoxon rank sum tests. For categorical data, chi-square tests were used. Sample characteristics and 95%-confidence intervals (95%-CI) for changes between intervention groups were calculated by means of the non-parametric Hodges-Lehmann estimator and a non-parametric confidence interval. In case of dichotomous data odds ratios and 95%-CI were presented. The analyses were performed by SAS Version 9.4 (SAS Institute Inc. (2023), Cary, NC).

### Ethics

The study was conducted in strict accordance with the ethical principles outlined in the Declaration of Helsinki. Measures were taken to ensure the confidentiality, safety, and welfare of all subjects. All participants provided informed consent prior to inclusion, and the research protocol was approved by the ethics committee of Saarland, Saarbruecken, Germany (file number: 70/2011) as well as by all local ethics committees, and the Bundesinstitut für Arzneimittel und Medizinprodukte (BfArM, Bonn, Germany).

### Role of funding source

The funders of the study had no role in study design, data collection, data analysis, data interpretation, or writing of the report.

## Results

### Patients

**Fig. 1:**
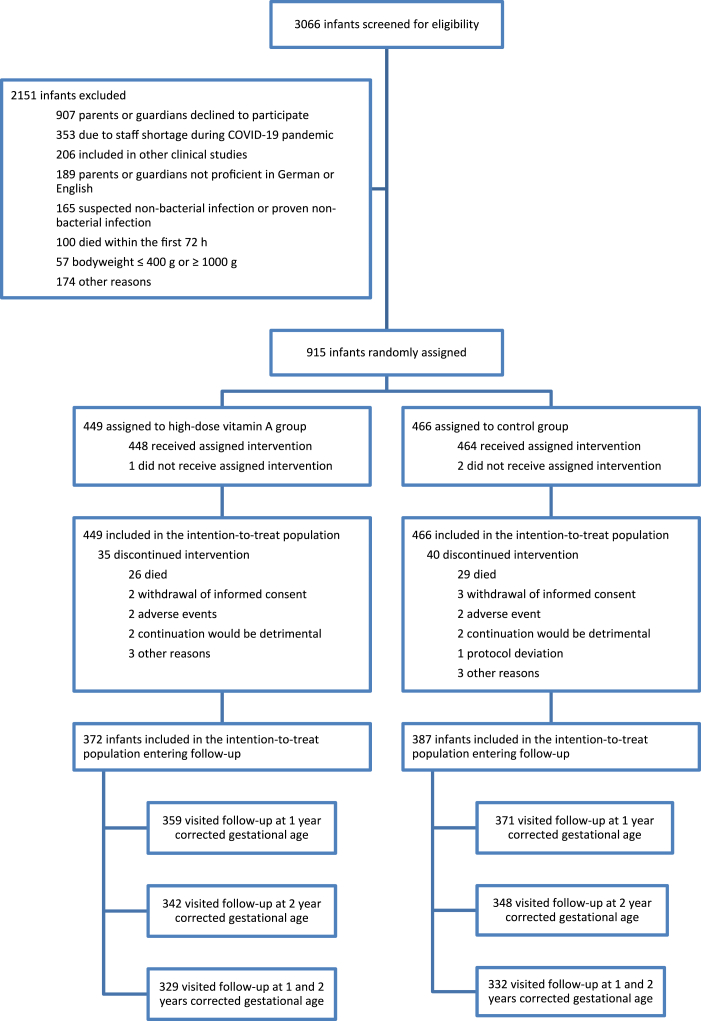
*Enrollment, allocation and analysis of NeoVitA patients*.

Between March 2015 and February 2022, 3066 infants born with a birth weight >400 g and <1000 g and gestational age ≤32 weeks' PMA were admitted to the participating NICUs and screened for eligibility. Finally, 915 of these infants were included in the intention-to-treat (ITT) analysis of the original NeoVita A-trial: 449 were randomized to the HD-VitA group and 466 to the control group. The NeoVita A follow-up study took place between September 2019 and June 2024 and included 759 (83%) patients of the initial study cohort: 372 infants in the HD-VitA group and 387 in the control group (Fig. 1). All-cause mortality during follow-up was n = 3 (0.8%) in the HD-VitA group and n = 6 (1.6%) in the control group. Follow-up at 12 and 24 months’ CA was visited by 661 (87.1%) patients: 329 infants in the HD-VitA group and 332 in the control group. Three patients (0.8%) in the HD-VitA group and 6 patients (1.6%) in the control group died during follow-up (OR 1.9, 95%-CI [0.5, 7.8], p = 0.34). Baseline characteristics of infants entering follow-up and their mothers were similar in both groups (Table 1 & Table S4).

**Table 1:** Birth history and anthropometric data at birth, 12- and 24-months of ITT patients available for follow-up.

	High-dose vitamin A group (n = 372)	Control group (n = 387)	Odds Ratio or median difference^a^ [95%-confidence interval]	p-value
Gender male	194 (52.2%)	199 (51.4%)	1.0 [0.7, 1.3]	0.84^a^
Birth				
Birth weight [g]	790 (400–995)	775 (400–995)	5.0^a^ [−15.0, 25.0]	0.65^b^
Birth length [cm]	33.0 (25.0–42.0)	33.0 (24.0–43.0)	0.0^a^ [0.0, 1.0]	0.31^b^
Birth head circumference [cm]	24.0 (19.0–28.0)	24.0 (19.0–29.0)	0.0^a^ [0.0, 0.0]	0.85^b^
Gestational age [weeks]	26.0 (23.0–32.0)	26.0 (23.0–32.0)	0.0^a^ [0.0, 0.0]	0.76^b^
Umbilical arterial pH	7.32 (6.8–7.6)	7.34 (6.9–7.6)	0.0^a^ [0.0, 0.0]	0.05^b^
Apgar score 5 min	8.0 (2.0–10.0)	8.0 (3.0–10.0)	0.0^a^ [0.0, 0.0]	0.84^b^
Apgar score 10 min	9.0 (3.0–10.0)	9.0 (4.0–10.0)	0.0^a^ [0.0, 0.0]	0.88^b^
Mode of delivery			–	0.76^a^
Assisted vaginal delivery	38 (10.2%)	38 (9.8%)		
Caesarean section	271 (72.9%)	287 (74.2%)		
Emergency caesarean section	63 (16.9%)	62 (16.0%)		
Birth complications			–	0.23^a^
Asphyxia	8 (2.2%)	2 (0.5%)		
Haemorrhage	1 (0.3%)	1 (0.3%)		
Others	23 (6.2%)	29 (7.5%)		
None	340 (91.4%)	355 (91.7%)		
Respiratory distress			1.0 [0.7, 1.4]	0.99^a^
Yes	297 (79.8%)	309 (79.8%)		
No	75 (20.2%)	78 (20.2%)		
Missing	0	0		
Sepsis			0.9 [0.4, 2.0]	0.76^a^
Yes	12 (3.2%)	11 (2.8%)		
No	360 (96.8%)	376 (97.2%)		
Missing	0	0		
12 Months				
Body weight [kg]	8.4 (5.1–12.8)	8.4 (5.7–13.0)	0.0^a^ [−0.2, 0.2]	0.92^b^
Length [cm]	73.1 (61.0–88.0)	73.0 (62.0–83.0)	−0.0^a^ [−0.6, 0.5]	0.82^b^
Head circumference [cm]	45.0 (37.0–54.0)	44.8 (38.5–50.5)	0.0^a^ [−0.2, 0.3]	0.87^b^
24 Months				
Body weight [kg]	11.0 (7.4–17.0)	10.8 (7.6–15.4)	0.1^a^ [−0.1, 0.3]	0.44^b^
Length [cm]	85.0 (71.5–99.2)	85.0 (74.0–99.0)	0.0^a^ [−0.7, 0.5]	0.84^b^
Head circumference [cm]	47.1 (39.0–51.4)	47.0 (41.0–54.2)	0.1^a^ [0.0, 0.5]	0.16^b^

Data depicted as absolute and relative numbers or in the median and range. In case of continuous parameters, the Hodges-Lehmann estimate of the difference^a^ and its non-parametric 95%-confidence interval is reported.

a Chi2-test.

b Wilcoxon-Mann-Whitney test.

### Respiratory outcomes

High-dose enteral fat-soluble vitamin A had no effect on the total number of antibiotics needed for pulmonary infections or on the total number of hospital admissions for pulmonary infections at 12 and 24 months' CA (Table 2), with a median of 1 course administered to infants in each arm by 12 months (median difference 0.0, 95%-CI [0.0, 0.0], p = 0.44). The median number of hospital admissions for pulmonary infections was 0 in the HD-VitA group and in the control group at 12 months' CA (median difference 0.0, 95%-CI [0.0, 0.0], p = 0.94). At 24 months’ CA, in median one course of antibiotics was administered to the HD-VitA group and control group for pulmonary infections (median difference 0.0, 95%-CI [0.0, 0.0], p = 0.97); the median number of hospital admissions for pulmonary infections per patient was 0.0 in the HD-VitA group and 1.0 in the control group at 24 months CA (median difference 0.0, 95%-CI [0.0, 0.0], p = 0.43).

**Table 2:** Pulmonary assessment at 12- and 24-months corrected age of ITT patients available for follow-up.

	12 months				24 months			
	High-dose vitamin A group (n = 372)	Control group (n = 387)	Odds Ratio or median difference^a^ [95%-confidence interval]	p-value	High-dose vitamin A group (n = 372)	Control group (n = 387)	Odds Ratio or median difference^a^ [95%-confidence interval]	p-value
Antibiotics administered			1.3 [0.9, 1.9]	0.15^a^			0.9 [0.6, 1.2]	0.44^a^
Yes	69 (21.7%)	89 (26.5%)			111 (36.0%)	106 (33.1%)		
No	249 (78.3%)	247 (73.5%)			197 (64.0%)	214 (66.9%)		
Missing	54	51			64	67		
Total number of courses of antibiotic treatments per patient	1.0 (1.0–10.0)	1.0 (1.0–6.0)	0.0^a^ [0.0, 0.0]	0.15^b^	1.0 (1.0–10.0)	2.0 (1.0–24.0)	0.0^a^ [0.0, 0.0]	0.20^b^
Total number of courses of antibiotic treatments because of pulmonary infections per patient	1.0 (0.0–5.0)	1.0 (0.0–6.0)	0.0^a^ [0.0, 0.0]	0.44^b^	1.0 (0.0–5.0)	1.0 (0.0–24.0)	0.0^a^ [0.0, 0.0]	0.97^b^
Hospital admissions			0.9 [0.7, 1.3]	0.61^a^			0.9 [0.6, 1.2]	0.47^a^
Yes	140 (42.8%)	141 (40.9%)			112 (36.3%)	109 (33.5%)		
No	187 (57.2%)	204 (59.1%)			197 (63.7%)	216 (66.5%)		
Missing	45	42			63	62		
Total number of hospital admissions per patient	1.0 (1.0–9.0)	1.0 (1.0–7.0)	0.0^a^ [0.0, 0.0]	0.46^b^	1.0 (1.0–11.0)	1.0 (1.0–10.0)	0.0^a^ [0.0, 0.0]	0.88^b^
Total number of hospital admissions because of pulmonary infections per patient	0.0 (0.0–7.0)	0.0 (0.0–4.0)	0.0^a^ [0.0, 0.0]	0.94^b^	0.0 (0.0–7.0)	1.0 (0.0–4.0)	0.0^a^ [0.0, 0.0]	0.43^b^

Data depicted as absolute and relative numbers or in the median and range. In case of continuous parameters, the Hodges-Lehmann estimate of the difference^a^ and its non-parametric 95%-confidence interval is reported.

a Chi2-test.

b Wilcoxon-Mann-Whitney test.

Recurrent wheezing was present in 5.2% in the HD-VitA group and 5.0% in the control group at 12 months' CA (OR 1.0, 95%-CI [0.5, 1.9], p = 0.91); and 5.4% in the HD-VitA group and 5.0% in the control group at 24 months’ CA (OR 0.9, 95%-CI [0.5, 1.8], p = 0.81).

### Neurodevelopmental assessment and neurological/non-neurological diseases

High-dose enteral fat-soluble vitamin A had no association with Bayley scores (Table 3). Of 759 infants, 92 completed the Bayley-II. The median Mental Development Index score was 95 vs. 97 (median difference −5.0, 95%-CI [−12.0, 2.0], p = 0.16) and Psychomotor Development Index 96 vs. 92 (median difference 3.0, 95%-CI [−4.0, 8.0], p = 0.40), with intervention and placebo, respectively. Among the 526 infants with Bayley-III scores, the median cognitive composite score was 95 vs. 95 (median difference 0.0, 95%-CI [−5.0, 0.0], p = 0.21) and motor score was 92 vs. 92 (median difference 0.0, 95%-CI [−4.0, 3.0], p = 0.48), respectively. At 24 months’ CA, 61 (16.4%) patients of the HD-VitA group and 80 (20.7%) patients of the control group participated in the follow-up, but neurodevelopmental assessment could not be accomplished for different reasons.

**Table 3:** Neurodevelopmental assessment at 24 months corrected age of the ITT patients available for follow-up.

	High-dose vitamin A group (n = 372)	Control group (n = 387)	Odds ratio or median difference^a^ [95%-confidence interval]	p-value
Bayley			1.1 [0.7, 1.7]	0.70^a^
Bayley-II	48 (15.4%)	44 (14.3%)		
Bayley-III	263 (84.6%)	263 (85.7%)		
Missing	61	80		
Bayley-II				0.16^b^
Mental development index	95.0 (49.0–145.0)	97.0 (50.0–128.0)	−5.0^a^ [−12.0, 2.0]	0.40^b^
Psychomotor development index	96.0 (45.0–134.0)	92.0 (50.0–142.0)	3.0^a^ [−4.0, 8.0]	
Bayley-III				
Cognitive scale	95.0 (28.0–145.0)	95.0 (45.0–145.0)	0.0^a^ [−5.0, 0.0]	0.21^b^
Language scale	87.0 (21.0–148.0)	91.0 (0.0–151.0)	−3.0^a^ [−6.0, 3.0]	0.43^b^
Motor scale	92.0 (45.0–140.0)	92.0 (45.0–134.0)	0.0^a^ [−4.0, 3.0]	0.48^b^

Data depicted as absolute and relative numbers or in the median and range. In case of continuous parameters, the Hodges-Lehmann estimate of the difference^a^ and its non-parametric 95%-confidence interval is reported.

a Chi2-test.

b Wilcoxon-Mann-Whitney test.

Nevertheless, about one-third of the study cohort presented neurological and non-neurological diseases during follow-up (Tables S5 & S6). However, there was no difference between study groups in any item.

### Drug treatment

Only a few patients needed specific drug treatment during follow-up (Table S7). Most common therapies at 24 months’ CA were oxygen supplementation (HD-VitA group 1.2% vs. control group 1.5%), use of laxatives (HD-VitA group 0.8% vs. control group 1.0%), and vitamins (HD-VitA group 0.8% vs. control group 0.8%).

## Discussion

In this trial we found that early additional high-dose enteral fat-soluble vitamin A supplementation in ELBW infants who were already receiving recommended enteral vitamin A supplementation did not impact important pulmonary and neurodevelopmental outcomes at 24 months’ CA.

Improvements in neonatal intensive care medicine such as the use of surfactant, antenatal steroids and non-invasive ventilatory support have altered BPD characteristics. The “new BPD” is histopathologically characterised by simple alveolar structures caused by premature interruption of intrauterine lung maturation. In contrast, the “old BPD”, defined by inflammation, fibrosis, emphysema and hypertrophic smooth muscle cells of the airways, is less common today.^29^^,^^30^ Most studies evaluating the longterm pulmonary outcome of preterm infants concentrate on patients with BPD. However, it is generally assumed that also premature infants without BPD are at risk for pulmonary morbidities.^29^^,^^30^ This thesis is supported by the fact that premature infants also exhibit pulmonary symptoms in childhood and adolescence, although many pulmonary morbidities lessen as children grow.^30^^,^^31^

During their first few years of life, children born prematurely carry a higher risk of being rehospitalised for pulmonary complications.^32^ Interestingly, when parents of preterm born babies were asked to describe important pulmonary outcomes, many of the outcomes they described (e.g., hospital readmission or pulmonary infections) had a direct impact on the parents or the whole family (e.g., fear, loss of work or lack of sleep). BPD, which is usually considered a major respiratory outcome in neonatal trials, is not perceived by parents and caregivers as a relevant or important outcome parameter.^33^ This is why we decided to evaluate the aforementioned pulmonary outcome parameters.

In our study, 36.3% of HD-VitA patients and 33.5% of control patients had been admitted to the hospital by their follow-up at 24 months CA (p = 0.47). When considering hospital admissions due to pulmonary infections per patient, our follow-up study demonstrated that the median hospital admission rate was low, with a median of 0 in the HD-VitA group and 1 in the control group (p = 0.43).

Prematurity is a risk factor for both pulmonary complications and neurological dysfunction and NDI. As one of the most common morbidities in premature infants, BPD represents an additional risk factor for impaired fine and gross motor development regardless of the gestational age.^34^ NDI is reported significantly more often in patients with moderate and severe BPD than in patients without or only mild BPD.^3^^,^^34^^,^^35^ Possible reasons for this are an impaired gas exchange (e.g., hypoxia), a prolonged hospital stay accompanied by reduced stimuli for neuromotor development, longer exposure to analgo-sedatives, administration of systemic steroids and reduced physical activity due to pulmonary impairment.^35^ Structural lung anomalies in patients with BPD are associated with alterations in the white matter of the central nervous system that could negatively affect the neurological development of premature born babies.^36^

Different BPD definitions make it difficult to compare studies of BPD and NDI. Katz et al.^37^ examined the association between the BPD severity and pulmonary and neurodevelopmental longterm outcome at 2 and 5 years CA according to different BPD definitions, namely the 2001-NICHD, 2018-NICHD and 2019-Jensen definitions. They demonstrated that children with severe/grade 3 BPD according to the 2001-NICHD and 2018-NICHD definitions had a higher risk for NDI or death than less severe BPD-grades. Conversely, patients with moderate/grade 2 BPD according to the 2019-Jensen definition were found to carry a significantly increased risk of NDI or death. Differences became apparent in respiratory morbidities at 2 years CA when diverse definitions were used: severe BPD according to the 2001-NICHD definition as well as grade 2/grade 3 BPD according to the 2018-NICHD and 2019-Jensen definition were associated with a significantly increased risk for respiratory morbidities.

Retinoic acid, the active metabolite of vitamin A, is a modulator of the development of the embryonic central nervous system. After fetal development is completed, retinoic acid signaling weakens, but it persists and has effects on neural plasticity in the hippocampus, olfactory bulb and hypothalamus.^38^ Our analysis revealed no significant differences in neurologic development between the HD-VitA group and control group at 24 months CA.

The risk of cerebral palsy and neurosensory disease like blindness, deafness, and cognitive impairment, is elevated in preterm infants and is aggravated by the increasing severity of BPD.^39^^,^^40^ We detected no differences regarding neurologic diseases at 24 months CA between the HD-VitA and control group (p = 0.92).

Serum retinol concentrations in our study were similar between groups before study intervention (immediately after birth), after study intervention, and at 36 weeks PMA. Serum retinol concentrations were numerically slightly lower at the end of the trial than those seen at baseline and after treatment. Notably, plasma retinol concentrations at 36 weeks of gestation in our study were slightly higher in the control group — albeit clinically non-significant — when compared with the high-dose vitamin A group. Retinol concentrations in our control group were almost twice those that Wardle and colleagues^41^ observed in their control group (0.12 μg/mL), indicating more efficient basic supplementation from parenteral and enteral nutrition (milk feeds) and basic supplements amounting to at least 1500 IU/kg per day. This more efficient basic vitamin A supplementation in our control group could have obscured any beneficial effect of high-dose supplementation, as indicated by a subgroup analysis in a 2021 systematic review and meta-analysis, showing that the positive effects of vitamin A in reducing bronchopulmonary dysplasia at 36 weeks postmenstrual age might be limited to studies involving baseline vitamin A intake of less than 1500 IU/kg per day.^42^ There is some evidence that lower serum retinol concentrations in ELBW infants are due to inflammation rather than vitamin A deficiency, because transport proteins of retinol — retinol-binding protein (RBP) and transthyretin (TTR) — are reduced by inflammation, and infants who later develop bronchopulmonary dysplasia have a preserved RBP–TTR ratio rather than a low RBP–TTR ratio as would be seen with vitamin A deficiency.^17^ Moreover, impaired hydrolysis of retinyl esters, reduced bioavailability of bile salts required to form micelles, or insufficient carrier proteins required for absorption could have contributed to poor vitamin A absorption in our high-risk group. Overall, our study results are consistent with a 2022 meta-analysis examining the effect of enteral vitamin A supplementation (varying from 1500 IU/kg per day to 5000 IU/kg per day) on respiratory outcomes, which showed no effect on the duration of mechanical ventilation, oxygen requirement at 36 weeks postmenstrual age, or moderate or severe bronchopulmonary dysplasia at 36 weeks postmenstrual age.^43^ Conversely, a 2022 umbrella review of systematic reviews and meta-analyses showed that vitamin A supplementation — mostly in studies involving parenteral supplemention — lowered the rate of bronchopulmonary dysplasia.^44^ The lack of effectiveness of enteral high-dose vitamin A might, at least partly, be attributable to insufficient retinol absorption, and our data analysis and interpretation is limited by the fact that we were unable to obtain serial serum retinol concentrations during the study intervention due to regulatory and ethical specifications (blood sample volumes). Also, because of technical problems, we could not quantify RBP and retinyl ester in this study.

The results of our NeoVita A trial may not be entirely generalisable to preterm infants of other ethnic backgrounds and geographic regions as the large majority of the study infants were Caucasian and the NICUs were located in Austria and Germany, which may also have different neonatal practices than some other regions of the world. There is ample evidence that efficacy of enteral high-dose vitamin A may be at least partially affected by insufficient enteral retinol absorption, and our data analysis and interpretation is limited by the fact that we were unable to obtain serial serum vitamin A concentrations during the study intervention because of regulatory and ethical concerns (blood sample volumes).^21^ Moreover, the vitamin A dose we selected was extrapolated from studies, expert opinion, and safety factors^13^^,^^41^ and may have been too low. We thus cannot exclude with certainty that higher doses, a longer time interval of high-dose vitamin A supplementation and more readily dissolvable and absorbable preparations of enteral vitamin A supplementation—most importantly water-soluble preparations—might have demonstrated a positive effect on relevant neonatal outcome parameters in this high-risk cohort.

We administered Bayley-III in most patients, which was introduced in Germany in 2014. However, because the NeoVitaA trial was carried out in a transition period between Bayley-II and Bayley-III, some individuals were assessed with Bayley-II. Results of Bayley-II are not directly comparable with Bayley-III, which is more restrictive in its evaluation.^45^ Today, Bayley-4 is used which has comparable scores to Bayley-III and uses an updated normative sample. Moreover, it has comparable scores to those on the Wechsler Preschool a Primary Scale of Intelligence (WPPSI-IV) and the Peabody Developmental Motor Scales 2nd edition (PDMS-2) which supports the changes made in the Bayley-III and carried forward in the Bayley-4.^46^ Since 85.1% of our follow-up cohort was evaluated by Bayley-III, these results are representative for our overall cohort and are also comparable to future data collected with Bayley-4 scores.

Finally, while we assessed the number of antibiotic treatments per patient as well as the number of hospital admissions per patient for pulmonary infections, we did not survey our cohort's immunization status or respiratory syncytial virus (RSV) prophylaxis, nor did we collect information on specific respiratory infections (e.g., RSV).

In summary, the NeoVitA trial did not demonstrate the superiority of high dose enteral vitamin A supplementation over placebo for the composite outcome of death or moderate/severe BPD, or evidence of longer-term respiratory and neurodevelopmental outcome parameters within the first 2 years of life in ELBW infants receiving basic vitamin A supplementation. Therefore, although high dose vitamin A may be considered safe, as no adverse effects were noted, higher dosages than those proposed by the ESPGHAN^20^ are not generally recommended in ELBW infants.

## Contributors

Martin Poryo was responsible for data compilation and analysis and writing the manuscript. He accessed and verified the underlying data.

Ludwig Gortner was co-chief investigator. He was responsible for study conception, realisation, including patient recruitment, patient safety, supervision, and inspiring the investigator group. Most unfortunately, he passed away during the course of this study, but he played an essential role from the very beginning, and his motivation and leadership inspired this manuscript.

Johannes Bay was study assistant, and was responsible for study realisation, data compilation, and critically revising the manuscript.

Axel Franz was responsible for study conception, data compilation, data analysis and critically revising the manuscript.

Harald Ehrhardt was responsible for data compilation and analysis and critically revising the manuscript.

Lars Klein was responsible for data compilation and analysis and critically revising the manuscript.

Judith Behnke was responsible for data compilation and analysis and critically revising the manuscript.

Tina Frodermann was responsible for data compilation and analysis and critically revising the manuscript.

Jutta Petzinger was responsible for data compilation and analysis and critically revising the manuscript.

Christoph Binder was responsible for data compilation and analysis and critically revising the manuscript.

Susanne Kirschenhofer was responsible for data compilation and analysis and critically revising the manuscript.

Anja Stein was responsible for data compilation and analysis and critically revising the manuscript.

Britta Hüning was responsible for data compilation and analysis and critically revising the manuscript.

Axel Heep was responsible for data compilation and analysis and critically revising the manuscript.

Eva Cloppenburg was responsible for data compilation and analysis and critically revising the manuscript.

Julia Muyimbwa was responsible for data compilation and analysis and critically revising the manuscript.

Torsten Ott was responsible for data compilation and analysis and critically revising the manuscript.

Julia Sandkötter was responsible for data compilation and analysis and critically revising the manuscript.

Norbert Teig was responsible for data compilation and analysis and critically revising the manuscript.

Susanne Wiegand was responsible for data compilation and analysis and critically revising the manuscript.

Michael Schroth was responsible for data compilation and analysis and critically revising the manuscript.

Andrea Kick was responsible for data compilation and analysis and critically revising the manuscript.

Donald Wurm was responsible for data compilation and analysis and critically revising the manuscript.

Corinna Gebauer was responsible for data compilation and analysis and critically revising the manuscript.

Knud Linnemann was responsible for data compilation and analysis, and critically revising the manuscript.

Jochen Kittel was responsible for data compilation and analysis, and critically revising the manuscript.

Christian Wieg was responsible for data compilation and analysis, and critically revising the manuscript.

Ursula Kiechl-Kohlendorfer was responsible for data compilation and analysis, and critically revising the manuscript.

Susanne Schmidt was responsible for data compilation and analysis, and critically revising the manuscript.

Ralf Böttger was responsible for data compilation and analysis, and critically revising the manuscript.

Wolfgang Thomas was responsible for data compilation and analysis, and critically revising the manuscript.

Francisco Brevis Nunez was responsible for data compilation and analysis, and critically revising the manuscript.

Antje Stockmann was responsible for data compilation and analysis, and critically revising the manuscript.

Thomas Kriebel was responsible for data compilation and analysis, and critically revising the manuscript.

Andreas Müller was responsible for data compilation and analysis, and critically revising the manuscript.

Daniel Klotz was responsible for data compilation and analysis, and critically revising the manuscript.

Patrick Morhart was responsible for data compilation and analysis, and critically revising the manuscript.

Donatus Nohr was responsible for study conception, vitamin A analysis and critical revision of the manuscript.

Hans Konrad Biesalski was responsible for study conception, vitamin A analysis and critical revision of the manuscript.

Eleni Z. Giannopoulou was study assistant, and was responsible for study realization, data compilation, and critically revising the manuscript.

Susanne Hilt was responsible for data compilation, data integrity, and critical review of the manuscript.

Stefan Wagenpfeil was responsible for initial statistical planning and study conception.

Nadja Haiden was responsible for data compilation and analysis.

Matthew Rysavy was responsible for critically revising the manuscript.

Christoph Härtel was responsible for data compilation and analysis, and critically revising the manuscript.

Christian Ruckes was responsible for data compilation and statistical analysis.

Anne Ehrlich was responsible for study implementation and supervision, data compilation, analysis and integrity, and drafting and critically revising the manuscript.

Sascha Meyer was chief investigator. He was responsible for study conception, realisation, including patient recruitment, patient safety, data compilation, data analysis, and writing the manuscript. He accessed and verified the underlying data.

## Data sharing statement

We agree that individual de-identified participant data (including data dictionaries) will be shared, including all relevant demographic data as well as data pertinent to our primary and all secondary research issues. We will also share and make available our study protocol and statistical analysis plan to the medical and neonatal research community. We will share our data with investigators whose proposed use of the data has been approved by an independent review committee (learned intermediary) identified for this purpose. Proposals should be directed to sascha.meyer@klinikum-karlsruhe.de. To gain access, data requestors will need to sign a data access agreement. Data will be available for 5 years after publication of the original NeoVita A trial.

## Declaration of interests

The vitamin A study medication and placebo were provided by Aristo Pharma GmbH, Berlin, Germany. Aristo Pharma GmbH had no influence on study protocol, data compilation, and data analysis.

Nadja Haiden received grants or contracts from Nestle, consulting fees from Nestle and ELGAN as well as payment or honoraria for lectures, presentations, speakers bureaus, manuscript writing or educational events from Nestle, ELGAN, Danone, Hipp, EFCNI, Chiesi, Baxter. She is Chair of the Committee on Nutrition at ESPGHAN.

Christoph Härtel received honoraria for lectures on complications after preterm birth from Chiesi. He is vice president of the German Society of Child and Adolescent Health.

The remaining authors have no conflict of interest to declare.

## Appendix A Supplementary data

Supplementary Tables

Collaborateurs

Clinical Trial Protocol

Statistical Analysis Plan
