# Supplementary material for: Two-year outcomes after early postnatal high-dose fat-soluble enteral vitamin A supplementation in extremely low birth weight infants: follow-up of the NeoVitaA randomized controlled trial
Source: eClinicalMedicine. 2025 Sep 15;89:103495. doi: 10.1016/j.eclinm.2025.103495 (PMC12675030; doi:10.1016/j.eclinm.2025.103495)
Supplement: Supplementary Tables [file mmc1.docx]

**Supplement**

***Table S4:*** *Maternal demographics and obstetric history of the ITT patients available for follow-up.*

|  | High-dose vitamin A group  (n = 372) | Control group  (n = 387) | Odds Ratio or median difference^a^ [95%-confidence interval] | p-value |
| --- | --- | --- | --- | --- |
| Age [years] | 31·0 (18·0 – 52·0) | 31·0 (17·0 – 52·0) | 0·0^a^ [-1·0, 1·0] | 0·61*°* |
| Weight [kg] | 78·0 (48·0 – 159·0) | 76·0 (38·0 – 174·0) | 1·0^a^ [-2·0, 3·0] | 0·70*°* |
| Height [cm] | 165·0 (140·0 – 186·0) | 167·0 (145·0 – 185·0) | 0·0^a^ [-1·0, 1·0] | 0·55*°* |
| Gravida including current pregnancy | 2·0 (1·0 – 14·0) | 2·0 (1·0 – 13·0) | 0·0^a^ [0·0, 0·0] | 0·17*°* |
| Parity including current pregnancy | 1·0 (1·0 – 12·0) | 1·0 (1·0 – 9·0) | 0·0^a^ [0·0, 0·0] | 0·41*°* |
| Current pregnancy:  Singleton  Multiple | 262 (70·4%)  110 (29·6%) | 270 (69·8%)  117 (30·2%) | 1·0 [0·7, 1·3] | 0·84^#^ |
| Premature rupture of membranes  Yes  No  Missing | 124 (33·3%)  248 (66·7%)  0 | 138 (35·8%)  248 (64·2%)  1 | 1·1 [0·8, 1·5] | 0·48^#^ |
| Prolonged rupture of membranes  Yes  No  Missing | 71 (19·1%)  300 (80·9%)  1 | 67 (17·4%)  319 (82·6%)  1 | 0·9 [0·6, 1·3] | 0·52^#^ |
| Pregnancy induced hypertension  Yes  No  Missing | 71 (19·1%)  301 (80·9%)  0 | 70 (18·1%)  316 (81·9%)  1 | 0·9 [0·7, 1·4] | 0·74^#^ |
| Pre-eclamptic toxemia  Yes  No  Missing | 81 (21·8%)  291 (78·2%)  0 | 69 (17·9%)  317 (82·1%)  1 | 0·8 [0·5, 1·1] | 0·18^#^ |
| Smoking during pregnancy  Yes  No  Missing | 40 (11·3%)  313 (88·7%)  19 | 36 (9·9%)  329 (90·1%)  22 | 0·9 [0·5, 1·4] | 0·52^#^ |
| Recreational drug use  Yes  No  Missing | 9 (2·5%)  347 (97·5%)  16 | 3 (0·8%)  364 (99·2%)  20 | 0·3 [0·1, 1·2] | 0·07^#^ |
| Other complications of pregnancy  Yes  No  Missing | 284 (76·8%)  86 (23·2%)  2 | 279 (72·5%)  106 (27·5%)  2 | 0·8 [0·6, 1·1] | 0·18^#^ |
| Prenatal infections incl. chorioamnionitis  Yes  No  Missing | 96 (25·8%)  276 (74·2%)  0 | 108 (28·1%)  277 (71·9%)  2 | 1·1 [0·8, 1·5] | 0·49^#^ |

*Data depicted as absolute and relative numbers or in the median and range In case of continuous parameters, the Hodges-Lehmann estimate of the differencea and its non-parametric 95%-confidence interval is reported, which may lead to degenerate confidence intervals ranging from 0 to 0. #Chi2-test, °Wilcoxon-Mann-Whitney test*

***Table S5:*** *Neurologic diseases during the first 12 respectively 24 months of the ITT patients available for follow-up.*

|  | 12 months | | | | 24 months | | | |
| --- | --- | --- | --- | --- | --- | --- | --- | --- |
|  | **High-dose vitamin A group**  **(n = 372)** | **Control group**  **(n = 387)** | **Odds Ratio [95%-confidence interval]** | **p-value** | **High-dose vitamin A group**  **(n = 372)** | **Control group**  **(n = 387)** | **Odds Ratio [95%-confidence interval]** | **p-value** |
| Neurologic diseases overall  Yes  No  Missing | 104 (28·0%)  268 (72·0%)  0 | 117 (30·2%)  270 (69·8%)  0 | 1·1 [0·8, 1·5] | 0·49^#^ | 130 (35·5%)  236 (64·5%)  6 | 137 (35·9%)  245 (64·1%)  5 | 1·0 [0·8, 1·4] | 0·92^#^ |
| Sensory disease  Yes  No  Missing | 45 (12·1%)  327 (87·9%)  0 | 64 (16·5%)  323(83·5%)  0 | 1·4 [1·0, 2·1] | 0·08^#^ | 72 (19·7%)  294 (80·3%)  6 | 71 (18·6%)  311 (81·4%)  5 | 0·9 [0·6, 1·3] | 0·71^#^ |
| Motor disease  Yes  No  Missing | 8 (2·2%)  364 (97·8%)  0 | 12 (3·1%)  375 (96·9%)  0 | 1·5 [0·6, 3·6] | 0·41^#^ | 13 (3·6%)  353 (96·4%)  6 | 17 (4·5%)  365 (95·5%)  5 | 1·3 [0·6, 2·6] | 0·53^#^ |
| Visual impairment  Yes  No  Missing | 39 (11·2%)  309 (89·8%)  24 | 59 (16·2%)  305 (83·8%)  23 | 1·5 [1·0, 2·4] | 0·05^#^ | 61 (18·3%)  273 (81·7%)  38 | 63 (18·3%)  281 (81·7%)  43 | 1·0 [0·7, 1·5] | 0·99^#^ |
| Use of corrective glasses  Yes  No  Missing | 20 (5·7%)  329 (94·3%)  23 | 21 (5·8%)  344 (94·2%)  22 | 1·0 [0·5, 1·9] | 0·99^#^ | 35 (10·5%)  299 (89·5%)  38 | 39 (11·3%)  305 (88·7%)  43 | 1·1 [0·7, 1·8] | 0·72^#^ |
| Hearing impairment  Yes  No  Missing | 3 (0·9%)  347 (99·1%)  22 | 7 (1·9%)  358 (98·1%)  22 | 2·3 [0·6, 8·8] | 0·23^#^ | 9 (2·7%)  326 (97·3%)  37 | 11 (3·2%)  334 (96·8%)  42 | 1·2 [0·5, 2·9] | 0·70^#^ |
| Use of hearing aids/Cochlea implant  Yes  No  Missing | 0 (0·0%)  350 (100·0%)  22 | 1 (0·3%)  364 (99·7%)  22 | - | 0·33^#^ | 2 (0·6%)  334 (99·4%)  36 | 0 (0·0%)  345 (100·0%)  42 | - | 0·15^#^ |
| Cerebral palsy  Yes  No  Missing | 6 (1·7%)  343 (98·3%)  23 | 8 (2·2%)  357 (97·8%)  22 | 1·3 [0·4, 3·7] | 0·65^#^ | 12 (3·6%)  324 (96·4%)  36 | 15 (4·4%)  330 (95·6%)  42 | 1·2 [0·6, 2·7] | 0·60^#^ |
| Developmental delay  Yes  No  Missing | 63 (18·0%)  287 (82·0%)  22 | 67 (18·5%)  295 (81·5%)  25 | 1·0 [0·7, 1·5] | 0·86^#^ | 84 (25·2%)  250 (74·8%)  38 | 87 (25·4%)  255 (74·6%)  45 | 1·0 [0·7, 1·4] | 0·93^#^ |
| Epilepsy  Yes  No  Missing | 3 (0·9%)  347 (99·1%)  22 | 6 (1·7%)  358 (98·3%)  23 | 1·9 [0·5, 7·8] | 0·34^#^ | 5 (1·5%)  331 (98·5%)  36 | 7 (2·0%)  338 (98·0%)  42 | 1·4 [0·4, 4·4] | 0·59^#^ |
| Obstructive hydrocephalus  Yes  No  Missing | 9 (2·6%)  342 (97·4%)  21 | 15 (4·1%)  350 (95·9%)  22 | 1·6 [0·7, 3·8] | 0·25^#^ | 11 (3·3%)  325 (96·7%)  36 | 12 (3·5%)  333 (96·5%)  42 | 1·1 [0·5, 2·4] | 0·88^#^ |
| Others (e.g., congenital, familial and genetic disorders, nervous system disorders, eye disorders, psychiatric disorders)  Yes  No  Missing | 23 (6·6%)  326 (93·4%)  23 | 24 (6·6%)  341 (93·4%)  22 | 1·0 [0·6, 1·8] | 0·99^#^ | 28 (8·3%)  308 (91·7%)  36 | 30 (8·7%)  315 (91·3%)  42 | 1·0 [0·6, 1·8] | 0·87^#^ |

*Data depicted as absolute and relative numbers or in the median and range. ^#^Chi^2^-test*

***Table S6:*** *Non-neurological diseases during the first 12 respectively 24 months of the ITT patients available for follow-up.*

|  | 12 months | | | | 24 months | | | |
| --- | --- | --- | --- | --- | --- | --- | --- | --- |
|  | **High-dose vitamin A group**  **(n = 372)** | **Control group**  **(n = 387)** | **Odds Ratio [95%-confidence interval]** | **p-value** | **High-dose vitamin A group**  **(n = 372)** | **Control group**  **(n = 387)** | **Odds Ratio [95%-confidence interval]** | **p-value** |
| Non-neurological diseases overall  Yes  No  Missing | 111 (29·8%)  261 (70·2%)  0 | 113 (29·2%)  274 (70·8%)  0 | 1·0 [0·7, 1·3] | 0·85^#^ | 106 (29·0%)  260 (71·0%)  6 | 99 (25·9%)  283 (74·1%)  5 | 0·9 [0·6, 1·2] | 0·35^#^ |
| Chronic lung disease^@^  Yes  No  Missing | 27 (7·7%)  323 (92·3%)  22 | 28 (7·7%)  335 (92·3%)  24 | 1·0 [0·6, 1·7] | 1·0# | 34 (10·1%)  302 (89·9%)  36 | 26 (7·6%)  315 (92·4%)  46 | 0·7 [0·4, 1·3] | 0·25^#^ |
| Recurrent wheezing  Yes  No  Missing | 18 (5·2%)  330 (94·8%)  24 | 18 (5·0%)  343 (95·0%)  26 | 1·0 [0·5, 1·9] | 0·91^#^ | 18 (5·4%)  316 (94·6%)  38 | 17 (5·0%)  325 (95·0%)  45 | 0·9 [0·5, 1·8] | 0·81^#^ |
| Short bowel syndrome  Yes  No  Missing | 10 (2·8%)  342 (97·2%)  20 | 6 (1·6%)  359 (98·4%)  22 | 0·6 [0·2, 1·6] | 0·28^#^ | 8 (2·4%)  330 (97·6%)  34 | 4 (1·2%)  341 (98·8%)  42 | 0·5 [0·1, 1·6] | 0·23^#^ |
| Feeding problems/failure to thrive  Yes  No  Missing | 52 (14·8%)  300 (85·2%)  20 | 53 (14·5%)  312 (85·5%)  22 | 1·0 [0·6, 1·5] | 0·92^#^ | 53 (15·6%)  286 (84·4%)  33 | 41 (11·9%)  304 (88·1%)  42 | 0·7 [0·5, 1·1] | 0·15^#^ |
| Others (e.g., congenital, familial and genetic disorders, infections, gastrointestinal disorders)  Yes  No  Missing | 49 (14·1%)  299 (85·9%)  24 | 53 (14·6%)  309 (85·4%)  25 | 1·0 [0·7, 1·6] | 0·83^#^ | 47 (13·9%)  291 (86·1%)  34 | 44 (12·9%)  297 (87·1%)  46 | 0·9 [0·6, 1·4] | 0·70^#^ |

*Data depicted as absolute and relative numbers. ^#^Chi^2^-test @As per individual physician´s assessment based on medical and drug history, clinical and laboratory findings* ***Table S7:*** *Use of specific drug treatment during the first 12 respectively 24 months of the ITT patients available for follow-up.*

|  | 12 months | | | | 24 months | | | |
| --- | --- | --- | --- | --- | --- | --- | --- | --- |
|  | **High-dose vitamin A group**  **(n = 372)** | **Control group**  **(n = 387)** | **Odds Ratio [95%-confidence interval]** | **p-value** | **High-dose vitamin A group**  **(n = 372)** | **Control group**  **(n = 387)** | **Odds Ratio [95%-confidence interval]** | **p-value** |
| Diuretics  Yes  No  Missing | 5 (1·5%)  341 (98·5%)  26 | 2 (0·6%)  360 (99·5%)  25 | 0·4 [0·1, 2·0] | 0·23^#^ | 1 (0·3%)  336 (99·7%)  35 | 0 (0·0%)  342 (100·0%)  45 | - | 0·31^#^ |
| Methylxanthines  Yes  No  Missing | 0 (0·0%)  341 (100·0%)  27 | 0 (0·0%)  361 (100·0%)  26 | - | - | 1 (0·3%)  336 (99·7%)  35 | 1 (0·3%)  341 (99·7%)  45 | 1·0 [0·1, 15·8] | 0·99^#^ |
| Steroids  Yes  No  Missing | 1 (0·3%)  344 (99·7%)  27 | 3 (0·8%)  359 (99·2%)  25 | 2·9 [0·3, 27·8] | 0·34^#^ | 2 (0·6%)  335 (99·4%)  35 | 2 (0·6%)  340 (99·4%)  45 | 1·0 [0·1, 7·0] | 0·99^#^ |
| Oxygen  Yes  No  Missing | 9 (2·6%)  339 (97·4%)  24 | 8 (2·2%)  355 (97·8%)  24 | 0·8 [0·3, 2·2] | 0·74^#^ | 4 (1·2%)  334 (98·8%)  34 | 5 (1·5%)  337 (98·5%)  45 | 1·2 [0·3, 4·7] | 0·75^#^ |
| Others (e.g., Vitamins, antianemia preparations, thyroid therapy, antihypertensives, proton pump inhibitors)  Yes  No  Missing | 44 (12·7%)  302 (87·3%)  26 | 54 (14·9%)  308 (85·1%)  25 | 1·2 [0·8, 1·8] | 0·40^#^ | 25 (7·4%)  313 (92·6%)  34 | 22 (6·4%)  320 (93·6%)  45 | 0·9 [0·5, 1·6] | 0·62^#^ |

*Data depicted as absolute and relative numbers. ^#^Chi^2^-test*
