# Supplementary material for: Two-year outcomes after early postnatal high-dose fat-soluble enteral vitamin A supplementation in extremely low birth weight infants: follow-up of the NeoVitaA randomized controlled trial
Source: eClinicalMedicine. 2025 Sep 15;89:103495. doi: 10.1016/j.eclinm.2025.103495 (PMC12675030; doi:10.1016/j.eclinm.2025.103495)
Supplement: Collaborateurs [file mmc2.docx]

**Collaborateurs**

|  | **First Name** | **Surnames** |
| --- | --- | --- |
| 5 | Rahel | Schuler |
| 5 | Birgit | Kampschulte |
| 5 | Annesuse | Schmidt |
| 5 | Svilen | Atanasov |
| 7 | Mark | Dzietko |
| 7 | Sebastian | Prager |
| 7 | Ioana | Bialas |
| 8 | Petra | Kramps |
| 8 | Sarah | Beckmann |
| 8 | Jürgen | Seidenberg |
| 10 | Katja | Majosthusmann |
| 10 | Jenny | Potratz |
| 10 | Alijda | van den Heuvel |
| 10 | Maria | Tekaat |
| 11 | Susanne | Dettmers |
| 12 | Marie-Therese | Unterweger |
| 12 | Heike | Nowak |
| 13 | Jens | Möller |
| 14 | Ulrich | Thome |
| 3 | Jörg | Arand |
| 3 | Beate | Luger |
| 3 | Christian A. | Maiwald |
| 3 | Martin | Heideking |
| 15 | Matthias | Heckmann |
| 16 | Annette | Keller-Wacherbauer |
| 16 | Holger | Michel |
| 41 | Tanja | Karen |
| 18 | Anna | Schmid |
| 18 | Yasmin | Pellkofer |
| 18 | Elke | Griesmaier-Falkner |
| 19 | Orsolya | Genzel-Boroviczeny |
| 42 | Raquel Mata | Fernandez |
| 20 | Stefan | Avenarius |
| 21 | Andrea | Czoske |
| 21 | Christoph | Block |
| 22 | Simone | Schwarz |
| 43 | Andreas | Jenke |
| 25 | Tamara | Grass |
| 26 | Martin | Kuntz |
| 27 | Fabian | Fahlbusch |
| 35 | Johannes | Pöschl |
| 35 | Jule | Metzger |
| 34 | Sebastian | Ronellenfitsch |
| 36 | Thomas | Schaible |
| 36 | Julia | Reinhard |
| 37 | Sonja | Trepels-Kottek |
| 38 | Jacqueline | Bauer |
| 39 | Egbert | Herting |
| 40 | Wolfram | Henn |
| 33 | Annette | Laupert |
| 33 | Rebecca | Jathe |

*^3^ University Hospital Tübingen, Department of Neonatology and Center for Paediatric Clinical Studies, Calwerstraße 7, 72076 Tübingen, Germany*

*^5^ University Hospital Giessen, Department of General Paediatrics and Neonatology, Feulgenstr.12, 35392 Gießen, Germany*

*^7^ University Hospital Essen, Clinic for Paediatrics I, Department of Neonatology, Hufelandstraße 55, 45147 Essen, Germany*

*^8^ Clinical Centre Oldenburg, Department of Neonatology, Paediatric Intensive Care, Paediatric Cardiology, Paediatric Pneumonology and Allergology, Rahel-Straus-Straße 10, 26133 Oldenburg, Germany*

*^10^ University Hospital Münster, Department of Neonatology, Albert-Schweitzer-Campus 1, Building A1, 48149 Münster, Germany*

*^11^ University Hospital Bochum, St. Josef-Hospital, Department of Neonatology and Paediatric Intensive Care, 44791 Bochum, Germany*

*^12^ Cnopf´sche Kinderklinik, Department of Neonatology and Paediatric Intensive Care, St.-Johannis-Mühlgasse 19, 90419 Nürnberg, Germany*

*^13^ Clinical Centre Saarbrücken, Department of Paediatrics, Winterberg 1, 66119 Saarbrücken, Germany*

*^14^ University Hospital Leipzig, Department of Neonatology, Liebigstraße 20a, Building 6, 04103 Leipzig, Germany*

*^15^ University Hospital Greifswald, Department of Neonatology and Paediatric Intensiv Care, Ferdinand-Sauerbruch-Straße, 17475 Greifswald, Germany*

*^16^ University Hospital Regensburg, University Children’s Hospital Regensburg (KUNOClinics), Clinic St. Hedwig, Steinmetzstr. 1-3, 93049 Regensburg, Germany*

*^18^ Medical University Innsbruck, Department of Paediatrics II, Neonatology, Anichstraße 35, 6020 Innsbruck, Austria*

*1^9^ Ludwig-Maximilians-University Munich, Dr. von Haunersches Kinderspital, Department of Neonatology, Lindwurmstrasse 4, 80337 München, Germany*

*^20^ University Hospital Magdeburg, Department of Paediatrics, Leipziger Straße 44, 39120 Magdeburg, Germany*

*^21^ Hospital Mutterhaus der Borromäerinnen, Deparment of Paediatrics, Feldstraße 16, 54290 Trier, Germany*

*^22^ Sana Hospital Duisburg, Department of Neonatology and Paediatric Intensive Care, Zu den Rehwiesen 9, 47055 Duisburg, Germany*

*^25^ University Hospital Bonn, Eltern-Kind-Zentrum (ELKI), Department of Neonatology and Paediatric Intensive Care, Building 30, Venusberg-Campus 1, 53127 Bonn, Germany*

*^26^ University Hospital Freiburg, Department of Neonatology, Breisacher Straße 62, 79106 Freiburg, Germany*

*^27^ University Hospital Erlangen, Department of Neonatology and Paediatric Intensive Care, Loschgestraße 15, 91054 Erlangen, Germany*

*3^3^ Universitätsmedizin der Johannes Gutenberg-Universität Mainz, Interdisziplinäres Zentrum Klinische Studien (IZKS), Langenbeckstraße 1, 55131 Mainz, Germany*

*^34^ Pediatric doctor’s office Ronellenfitsch, Ringstrasse 1, 69168 Wiesloch, Germany*

*^35^ University Hospital Heidelberg, Department of Neonatology, Im Neuenheimer Feld 430, 69120 Heidelberg, Germany*

*^36^ University Hospital Mannheim, Department of Neonatology, Theodor-Kutzer-Ufer 1-3, 68167 Mannheim, Germany*

*^37^ University Hospital Aachen, Department of Neonatology, Pauwelsstraße 30, 52074 Aachen, Germany*

*^38^ Wolfsburg Clinic, Clinic for Pediatrics and Adolescent Medicine and Psychosomatics,* *Sauerbruchstr. 7, 38440 Wolfsburg, Germany*

*^39^ University Hospital Schleswig-Holstein, Clinic for Paediatrics, Ratzeburger Allee 160, Building A, 23538 Lübeck, Germany*

*^40^ Saarland University, Faculty M, Clinical Human Genetics, Building 68, 66421 Homburg, Germany*

*^41^ Lucerne Cantonal Hospital, Obesity Center, Spitalstrasse, 6004 Luzern, Switzerland*

*^42^ M1 Praxisklinik, Private Practice Gauting, Pippinplatz 4, 82131 Gauting*

*^43^ Kassel Clinic, Department of Neonatology and General Paediatrics, Mönchebergstr. 41-43, Haus F, 34125 Kassel, Germany*
