# Supplementary material for: Two-year outcomes after early postnatal high-dose fat-soluble enteral vitamin A supplementation in extremely low birth weight infants: follow-up of the NeoVitaA randomized controlled trial
Source: eClinicalMedicine. 2025 Sep 15;89:103495. doi: 10.1016/j.eclinm.2025.103495 (PMC12675030; doi:10.1016/j.eclinm.2025.103495)
Supplement: Clinical Trial Protocol [file mmc3.pdf]

CLINICAL TRIAL PROTOCOL

A prospective, multicenter, double blind, placebo-controlled, two-arm parallel group phase 3 trial to evaluate the effect of early postnatal additional high dose oral vitamin A supplementation of 5000 IU/kg/d versus placebo for 28 days for preventing bronchopulmonary dysplasia (BPD) or death in extremely low birth weight (ELBW) infants.

NeoVitaA Trial

EudraCT:	**2013-001998-24**
Sponsor trial code:	**ME 3827/1-1**
IZKS trial code:	**2012-008 NeoVitaA**
Draft/final:	**Final**
Version:	**5.1**
Date:	**25.11.2019**

Sponsor

Saarland University
Postfach 15 11 50
66041 Saarbrücken

represented by the Vice-president for Research and Technology Transfer

represented by the scientific member of the executive board
Prof. Dr. Martina Sester

Operating Institution of Sponsor

Institution:	Saarland University, Department of Pediatrics and Neonatology Homburg/Saar
Name:	**Prof. Dr. med. Sascha Meyer**
Address:	Neonatal Intensive Care Unit, Department of Pediatrics and Neonatology, University Children’s Hospital of Saarland, Campus Homburg, Building 9, FR 2.15 66421 Homburg/Saar, Germany
Phone.:	+49 (0) 6841 16-28 313 (direct line)
	+49 (0) 6841 16-28 301 (director’s secretary)
Fax:	+49 (0) 6841 16-28 310
E-mail:	sascha.meyer@uks.eu

This protocol contains confidential information and is intended solely for the guidance of the clinical trial. This protocol may not be disclosed to third parties not associated with the clinical trial or used for any other purpose without prior written consent of the Sponsor.

Coordinating Investigator

Institution:	Saarland University, Department of Pediatrics and Neonatology Homburg/Saar
Name:	**Prof. Dr. med. Sascha Meyer**
Address:	Neonatal Intensive Care Unit, Department of Pediatrics and Neonatology,<br>University Children's Hospital of Saarland,<br>Campus Homburg, Building 9, FR 2.15<br>66421 Homburg/Saar, Germany
Phone.:	+49 (0) 6841 16-28 313 (direct line)<br>+49 (0) 6841 16-28 301 (Director's secretary)
Fax:	+49 (0) 6841 16-28 310
E-mail:	sascha.meyer@uks.eu

Biometrician

Institution	Interdisciplinary Center for Clinical Trials (IZKS)<br>at the University Medical Center of the Johannes Gutenberg-University Mainz
Name:	**Christian Ruckes, Dipl. Math.**
Address:	Langenbeckstr. 1<br>55131 Mainz, Germany
Phone:	+49 (0)6131 / 17 9919
Fax:	+49 (0)6131 / 17 9914
E-Mail:	ruckes@izks-mainz.de

Trial Coordination

Operational

Institution	Interdisciplinary Center for Clinical Trials (IZKS)<br>at the University Medical Center of the Johannes Gutenberg-University Mainz
Name:	**Dr. med. Anne Ehrlich, MD**
Address:	Langenbeckstr 1<br>55131 Mainz, Germany
Phone:	+49 (0)6131 / 17 9909
Fax:	+49 (0)6131 / 17 9914
E-Mail:	ehrlich@izks-mainz.de

Safety Management

Institution	Interdisciplinary Center for Clinical Trials (IZKS)<br>at the University Medical Center of the Johannes Gutenberg-University Mainz
Name:	**Christoph Medler, MD, Safety Physician**
Address:	Langenbeckstr. 1<br>55131 Mainz, Germany
Phone:	+49 (0)6131 / 17 9894
Fax:	+49 (0)6131 / 17 9914
E-Mail:	medler@izks-mainz.de

Monitoring

Institution Interdisciplinary Center for Clinical Trials (IZKS)
at the University Medical Center of the Johannes Gutenberg-University Mainz

Name: **Annette Laupert, Senior CRA**

Address: Langenbeckstr. 1
55131 Mainz, Germany
Phone: +49 (0)6131 / 17 9891
Fax: +49 (0)6131 / 17 9925
E-Mail: laupert@izks-mainz.de

Data Management

Institution Interdisciplinary Center for Clinical Trials (IZKS)
at the University Medical Center of the Johannes Gutenberg-University Mainz

Name: **Anja Powaska**

Address: Langenbeckstr. 1
55131 Mainz, Germany
Phone: +49 (0)6131 / 17 9922
Fax: +49 (0)6131 / 17 9914
E-Mail: powaska@izks-mainz.de

Abbreviations

AE	Adverse Event
ALP	Alkaline Phosphatase
ALT	Alanine Aminotransferase
AMG	Arzneimittelgesetz (German drug law)
AST	Aspartate Aminotransferase
BfArM	Bundesinstitut für Arzneimittel und Medizinprodukte (Federal Institute for Drugs and Medical Devices)
BPD	Bronchopulmonary Dysplasia
BW	Birth Weight
CHE	Cholinesterase
CLD	Chronic Lung Disease
CA	Corrected gestational age
CPAP	Continuous Positive Airway Pressure
CRF	Case Report Form
CUSS	Cranial Ultrasound Scan
CV	Curriculum Vitae
DBS	Dried Blood Spot
DMP	Data Management Plan
DSMB	Data Safety Monitoring Board
EC/IEC	Ethics Committee/Independent Ethics Committee
eCRF	Electronic Case Report Form
ELBW	Extremely Low Birth Weight Infant (< 1000 g)
GA	Gestational Age
GBA	Gemeinsamer Bundesausschuss
GCP	Good Clinical Practice
GCP-V	GCP-Verordnung (German GCP Regulation)
GI	Gastrointestinal
Hb	Hemoglobin
HFNC	High Flow Nasal Cannula
HFOV	High-frequency oscillatory ventilation
HR	Heart Rate
I.M.	Intramuscular
I.V.	Intravenous
ICH	International Conference on Harmonization of Technical Requirements for Registration of Pharmaceuticals for Human Use
IMP	Investigational Medicinal Product
IMPD	Investigational Medicinal Product Dossier
INN	International Nonproprietary Name
INR	International Normalized Ratio
ISF	Investigator Site File
ITT	Intention To Treat
IVH	Intraventricular Hemorrhage
IZKS	Interdisciplinary Center for Clinical Trials
LKP	Leiter der Klinischen Prüfung (Coordinating Investigator according to German drug law)
MedDRA	Medical Dictionary for Regulatory Activities Terminology
NDI	Neurodevelopmental Impairment
NEC	Necrotizing Enterocolitis
NGT	Naso-gastric tube

NICU	Neonatal Intensive Care Unit
OGT	Oro-gastric tube
PMA	Postmenstrual Age
PN	Parenteral Nutrition
PNA	Postnatal Age
PNS	Postnatal Steroids
PO	Per Os
PP	Per Protocol
PPS	Positive Pressure Support
PPV	Positive Pressure Ventilation
PVL	Periventricular Leukomalacia
RBP	Retinol Binding Protein
RDE	Remote data entry
RCT	Randomized Controlled Trial
RDR	Relative Dose Response
RDS	Respiratory Distress Syndrome
RE	Retinyl Ester
ROP	Retinopathy of Prematurity
RR	Respiratory Rate
SAE	Serious Adverse Event
SAP	Statistical Analysis Plan
SOP	Standard operating procedure
SUSAR	Suspected Unexpected Serious Adverse Reaction
VA	Vitamin A
VAD	Vitamin A Deficiency
VLBW	Very Low Birth Weight Infant (< 1500 g)
WBC	White Blood Cell Count

Synopsis

**Title**	A prospective, multicenter, double blind, placebo-controlled, two-arm parallel group phase 3 trial to evaluate the effect of early postnatal additional high dose oral vitamin A supplementation of 5000 IU/kg/d versus placebo for 28 days for preventing bronchopulmonary dysplasia (BPD) or death in extremely low birth weight (ELBW) infants.
**Short title**	NeoVitaA Trial
**EudraCT**	2013-001998-24
**Sponsor trial code**	ME 3827/1-1
**IZKS trial code**	2012-008 NeoVitaA
**Indication**	Bronchopulmonary Dysplasia
**Phase**	III
**Trial Treatments**	Test product: 5000 IU/kg/d of vitamin A oral solution (retinyl palmitate in peanut oil, Vitadral® Drops)<br>Reference therapy: Placebo oral solution (peanut oil)<br>Treatment of trial medication is applied for 28 days commencing within 72hrs after birth
**Primary objective**	To investigate whether early postnatal additional high dose oral vitamin A supplementation (5000 IU/kg body weight/day) for 28 days reduces the absolute risk of bronchopulmonary dysplasia (moderate/severe) or death at 36+0 weeks PMA or at date of discharge to home, whichever comes first in ELBW infants when compared to placebo treatment.
**Secondary objectives**	To assess the impact on high dose oral vitamin A supplementation on:<br>1. All-cause mortality<br>2. All grade BPD (mild/moderate/severe)<br>3. Duration of PPV and PPS<br>4. Duration of evolving BPD since birth<br>5. Serum VA status<br>6. Retinopathy of prematurity (ROP)<br>7. Intraventricular hemorrhage (IVH)<br>8. Periventricular leukomalacia (PVL)<br>9. Necrotizing enterocolitis (NEC)<br>10. Safety and tolerability of trial medication<br>11. Pulmonary assessment at 12 and 24 months c. a..<br>12. Neurological assessment at 24 months c.a.<br>13. Anthropoemetric data
**Trial design**	A prospective, multicenter, double-blind, randomized, placebo-controlled parallel group trial
**Trial population**	**Inclusion criteria:**<br>Subjects meeting all of the following criteria will be considered for enrolment in the trial:<br>- Neonates with a birth weight <1000 g<br>- Neonates with a gestational age < 32 + 0 weeks at time of inclusion into the trial<br>- Who receive any oxygen supplementation or respiratory support after admission to the NICU within 72 hours following birth<br>- Who (will) receive a basic VA supplementation of 1000 IU/kg body weight/day (7000 IU/kg body weight/week)<br>- Postnatal age <72 hours of life<br>- Minimal/enteral feeds commenced<br>- Signed and dated informed parental consent<br>**Exclusion criteria:**<br>Subjects presenting with any of the following criteria will not be enrolled in the trial:<br>- ≥1 major congenital abnormalities<br>- Congenital nonbacterial infection with overt signs at birth

	- Terminal illness as evidenced by pH <7.0 for >2 hours or persistent bradycardia (heart rate <100 bpm) associated with hypoxia for >2 hours<br>- Birth weight <400 g<br>- Participation in another clinical trial according to German Drug Law (AMG)
**Trial duration and dates**	First subject in: Q1 2015<br>Last subject in: Q4 2020<br>Last subject out: Q2 2021<br>Last Subject Follow up: Q2 2023<br>Duration of the trial 72 months
**Number of subjects**	To be screened: 1000 subjects<br>To be enrolled/randomized: 914 subjects (expected screening failure rate of 10%)
**Number of sites**	> 25 trial sites are planned to participate.
**Primary endpoint**	Combined Incidence of BPD (moderate/severe) or death at 36+0 weeks PMA or at date of discharge to home, whichever comes first.
**Secondary endpoints**	1. Any death<br>2. Any occurrence of BPD (mild/moderate/severe)<br>3. Days of PPV or PPS<br>4. Days of evolving BPD<br>5. Serum retinol levels, RBP, RE<br>6. Diagnosis of ROP using ophthalmological examination<br>7. Diagnosis of all grades of IVH using cranial ultrasound investigation<br>8. Diagnosis of PVL using cranial ultrasound investigation<br>9. Diagnosis of NEC using clinical examination and X-ray investigation<br>10. Occurrence of any adverse event<br>11. Total number of antibiotic treatments at 12 and 24 months of c.a.<br>12. Total number of antibiotic treatments for pulmonary infections at 12 and 24 months of c.a.<br>13. Total number of hospital admissions at 12 and 24 months of c.a.<br>14. Total number of hospital admissions for pulmonary infections at 12 and 24 months of c.a.<br>15. Results of Bayley-II or Bayley III scale at 24 months of c.a.<br>16. Anthropometric data at 12 and 24 months of c.a.<br>17. Neurological and non-neurological diseases during the first 12 and 24 months c.a.<br>18. Use of supportive therapies and specific drug treatment
**Statistical analysis**	Primary analysis variable:<br>- Evaluation of the primary endpoint is planned in the ITT population by a logistic regression analysis with treatment group and trial site as covariates. The global, two-sided significance level is set to 0.05.<br>Secondary analysis variables:<br>- Evaluation of secondary outcomes is explorative. Descriptive statistical analyses are used for each of the two trial groups separately. For quantitative variables mean and standard deviation and for qualitative variables absolute and relative frequencies are computed, respectively.<br>Interim analysis: There is one interim analysis after 50% of subjects have reached 36+0 weeks PMA or date of discharge to home, whichever comes first. 2 sequential tests are made and the O'Brien-Fleming alpha spending function is used to account for the issue of multiple testing. The global two-sided significance level is 0.05. No stop for futility is incorporated in this design.

Table of Contents

Abbreviations 4
Synopsis 6
Table of Contents 8
1 INTRODUCTION 11
1.1 Scientific background 11
1.2 Trial rationale 14
1.3 Treatments and rationale for dose selection 16
1.4 Summarized risk benefit assessment 20
2 TRIAL OBJECTIVES 23
2.1 Primary objective 23
2.2 Secondary objectives 23
3 TRIAL DESIGN 24
3.1 Trial duration and schedule 25
3.2 Number of subjects and centers 25
3.3 Primary and secondary endpoints 26
3.3.1 Primary endpoint 26
3.3.2 Secondary endpoints 26
3.4 Measures taken to minimize/avoid bias 27
3.4.1 Randomization 27
3.4.2 Blinding 27
3.5 Selection and withdrawal of subjects 27
3.5.1 Inclusion criteria 27
3.5.2 Exclusion criteria 28
3.5.3 Required during the trial 28
3.5.4 Withdrawal criteria 28
3.5.5 Premature closure of trial sites 28
3.5.6 Premature closure of the clinical trial 29
4 TRIAL TREATMENTS 29
4.1 Investigational Medicinal Product 29
4.1.1 General information about investigational medicinal product (IMP) 29
4.1.2 Therapeutic effects 29
4.1.3 Known side effects 30
4.1.4 Dosage schedule and administration of the trial drug 30
4.1.5 Treatment assignment 31
4.1.6 Treatment after the end of the trial 31
4.1.7 Overdose instructions 31
4.1.8 Emergency procedures or treatment, rescue medication 31
4.1.9 Packaging and labeling 32
4.1.10 Drug storage, supplies and accountability 32
4.1.11 Procedures for monitoring treatment compliance 33
5 Trial Schedule 33
5.1 Review of clinical records and *Informed Consent* procedures 35
5.2 Baseline visit 35
5.3 Daily visits during trial treatment 36
5.4 Post-treatment visits 37
5.5 End of trial visit 37
5.6 Follow-up (FU) Visits 38
5.6.1 12 month-FU-visit 38
5.6.2 24 month-FU-visit 38

6 TRIAL METHODS 38
6.1 Assessment of efficacy 38
6.1.1 Assessment of evolving BPD 38
6.1.2 Assessment of BPD 39
6.1.3 End-of-trial assessment 40
6.1.4 Assessment of neurological development 41
6.2 Blood tests for VA status 41
6.3 Assessment of safety and tolerability 42
6.3.1 Assessment of clinical condition 42
6.3.2 Assessment of complications and sequelae of prematurity 43
6.3.3 Blood tests – Safety 44
6.3.4 Cranial Ultrasound Investigations 45
6.3.5 Adverse events 45
6.4 Other assessments 45
6.4.1 Maternal medical history 45
6.4.2 Maternal obstetric history 45
6.4.3 Subject's medical history 46
6.4.4 Growth 46
6.4.5 Nutritional assessment 46
6.4.6 End-of-trial assessment 47
7 SAFETY 47
7.1 Definitions 47
7.2 Assessment of AEs by investigator 49
7.3 Period of observation 50
7.4 Documentation of AEs and Follow-up 50
7.5 Immediate reporting of SAEs by investigator 51
7.6 Safety evaluation and reporting by Sponsor 51
7.7 Immediate reporting of pregnancy by investigator 51
7.8 Documentation of Abuse, Misuse, Overdose and Medication Error 51
7.9 Emergency procedures 52
7.10 Other safety data 52
7.10.1 Previous and intercurrent illnesses 52
7.10.2 Previous and intercurrent medical treatments 52
8 STATISTICS 52
8.1 Sample size 52
8.2 Analysis populations 53
8.3 Efficacy analyses 53
8.3.1 Definition and analysis of primary endpoint 53
8.3.2 Analysis of secondary endpoints 53
8.3.3 Analysis of subgroups 53
8.3.4 Interim analyses 54
8.4 Analysis of adverse events 54
8.5 Analysis of clinical laboratory findings 54
8.6 Analysis of Follow-up 54
9 QUALITY CONTROL AND QUALITY ASSURANCE 55
9.1 Requirements for investigator, investigational sites and members of the investigational staff 55
9.2 Direct entries 55
9.3 Direct access to source data/documents 55
9.4 Investigator site file and archiving 55
9.5 Monitoring 56
9.6 Risk Management 56
9.7 Measures to secure compliance 56

9.8 Inspection by authorities 56
9.9 Audits 57
10 DATA MANAGEMENT 57
10.1 Responsibilities 57
10.2 Data collection 57
10.3 Data handling 57
10.4 Storage and archiving of data 58
11 ETHICAL AND LEGAL ASPECTS 58
11.1 Good clinical practice 58
11.2 Subject information and *Informed Consent* 58
11.3 Confidentiality 59
11.4 Responsibilities of investigator 59
11.5 Approval of trial protocol and substantial amendments 59
11.6 Continuous information to independent ethics committee 60
11.7 Submission to local regulatory/competent authorities 60
11.8 Independent data safety and monitoring board (DSMB) 60
11.9 Insurance 60
11.10 Agreements 61
11.10.1 Financing of the trial 61
11.10.2 Report 61
11.10.3 Publication policy 61
12 SIGNATURES 62
13 DECLARATION OF INVESTIGATOR 63
14 REFERENCES 64

1 INTRODUCTION

This document is a protocol for a human research trial. The planned trial will be conducted in compliance with this protocol, Good Clinical Practice (GCP) standards and all applicable regular and ethical regulations.

1.1 Scientific background

Summary

Bronchopulmonary dysplasia (BPD) affects as many as 35% of extremely low birth weight infants (ELBW; birth weight < 1000 g) [1, 2]. BPD is defined as the need for oxygen supplementation at 36 weeks postmenstrual age or at date of discharge to home, whichever comes first (PMA) [3]. The disease is marked by respiratory compromise and is associated with high mortality and severe long-term morbidity [4], including cerebral palsy. Unfortunately, many therapeutic approaches are non-effective (e.g., inhaled nitric oxide) or have significant side effects (administration of steroids). The beneficial role of intramuscular (i.m.) vitamin A (VA) supplementation has been demonstrated in a recent Cochrane meta-analysis; however, because of the substantial pain associated with repetitive i.m. injection, this form of VA supplementation is not common practice. Moreover, recent data indicate that there is substantial variation in how and how much VA is given to preterm infants in different Neonatal Intensive Care Units (NICUs).

The aim of this trial is to assess the role of early postnatal additional high-dose oral VA supplementation versus placebo for 28 days for preventing BPD or death in ELBW infants.

Bronchopulmonary dysplasia is associated with an increased mortality rate and continues to carry significant long-term morbidity. Although a variety of medical interventions have been examined, most of them have proven to be futile (e.g., inhaled nitric oxide), or the interventions themselves carry substantial side effects (e.g., early administration of steroids). The intra-muscular administration of VA has been shown to be effective in significantly reducing the rate of BPD in preterm infants. However, given the fact that i.m. VA injections are painful, and that they have to be repeated several times, this practice has not been widely accepted in neonatal intensive care medicine.

Therefore, a randomized controlled trial (RCT) that will assess the role of early postnatal additional high-dose oral VA supplementation to reduce and prevent the occurrence of BPD or death in this susceptible cohort (ELBW infants) is warranted.

Bronchopulmonary Dysplasia and Prematurity

Bronchopulmonary dysplasia remains a major threat to preterm neonates (<32 weeks gestational age; 1.4% of all live births in Germany) and is currently being observed in up to 20% of preterm neonates and as many as 35% of ELBW infants [1, 2]. Main clinical risk factors that have been identified include degree of prematurity, pre- and postnatal infections, postnatal asphyxia, intrauterine growth retardation (IUGR), and male gender.

Therapy of BPD is still controversial. An increase in survival rates of neonates without BPD has been demonstrated following introduction of surfactant replacement therapy about 20 years ago. Therefore, intratracheal surfactant administration has become standard therapy in neonatal intensive care treatment. However, other pharmacological treatments remain controversial. Postnatal steroids (PNS) reduce the incidence of BPD; however, this has come at the expense of an increased number of neonates with neurodevelopmental impairment (NDI). Therefore, this approach is reserved for neonates with life-threatening pulmonary dysfunction. Low-dose inhaled nitric oxide failed to reduce the incidence of BPD in very preterm infants in a European clinical trial enrolling more than 1,600 infants [5], which stresses the need for further clinical research.

Short-term effects of BPD during early childhood include an increased risk of re-hospitalization, mainly secondary life-threatening acute respiratory infections, and over the long term, an increased risk for asthma and persistent airway hyperresponsiveness until the early adult age. The most critical impact on long-term outcome represents the association of BPD with NDI, even in the absence of PNS administration. A very recently published trial from the NICDH network proved the association of BPD with spastic diplegia [6].

Vitamin A is one of the most important micronutrients affecting the health of children. Recognized for nearly 90 years as an essential dietary constituent, it is vital for orderly growth and differentiation of tissues. In the developing world, VA supplementation programs have significantly reduced infant mortality as well as the incidence of xerophthalmia, respiratory infection, and morbidity from gastrointestinal infection [7]. Supplementing newborn infants with VA within the first two days of life reduces infant mortality by almost 25%, with the greatest benefit to those of low birth weight [8]. The World Bank estimates that VA supplementation is one of the most cost-effective health interventions available [9]. In the developed world, most infants and children are VA sufficient. Term infants are well supplied with VA in utero (at the expense of maternal stores), and both human milk and infant formulae contain adequate amounts of VA for normal growth and health in the first six months of life [10]. Clinical vitamin A deficiency (VAD) occurs rarely, and almost exclusively in children with malabsorptive disorders.

Unfortunately, VA sufficiency cannot be assumed for preterm infants [11]. This important subgroup of our infant population is born with inadequate body stores of VA, is often unable to tolerate routine oral supplementation, and is prone to diseases of the respiratory and gastrointestinal tract, and the eye. Preterm infants have low plasma concentrations of both retinol and retinol binding protein (RBP) at birth compared with term infants, and this reflects low hepatic stores [11, 12]. Plasma concentrations of retinol remain low throughout the first year of life [11-13]. These issues and problems have been recognized for some 20 years; yet preterm infants (particularly ELBW infants remain at risk of VAD [14].

Uptake and metabolism of vitamin A

The term VA refers to a group of compounds, including retinol, retinal aldehyde, and retinoic acid. Retinol may be obtained directly from foods of animal origin or be formed in the body from metabolism of ß-carotene [11]. Absorption of dietary retinyl esters (REs) is complex, involving hydrolysis and complexation with bile acids in the gut lumen before uptake by enterocytes [15]. Metabolism of VA within these cells and subsequent transfer into the lymphatic system depends on a specific carrier protein, cellular retinol binding protein type 2, the availability of which may be limited in the preterm infant [16]. After absorption, retinol is bound to RBP in the liver and transported in plasma as the retinol-RBP complex, bound in a 1:1 ratio with transthyretin. Circulating retinol is delivered to target tissues via a specific membrane receptor and is oxidized within the cell to its active metabolite, retinoic acid. The precise mechanisms by which retinoic acid affects intracellular activity are complex and incompletely defined [17]. Some 90% of the body's reserve of VA is stored in the liver as retinyl esters: other sites of major VA storage include the eye and the lung [11].

Figure 1 adapted from Mactier and Weaver, 2005 [11]: Uptake and metabolism of VA. CRBP II, cellular retinol binding protein type 2; IV, intravenous; IM, intramuscular; RSB, retinol binding protein; TTR, transthyretin

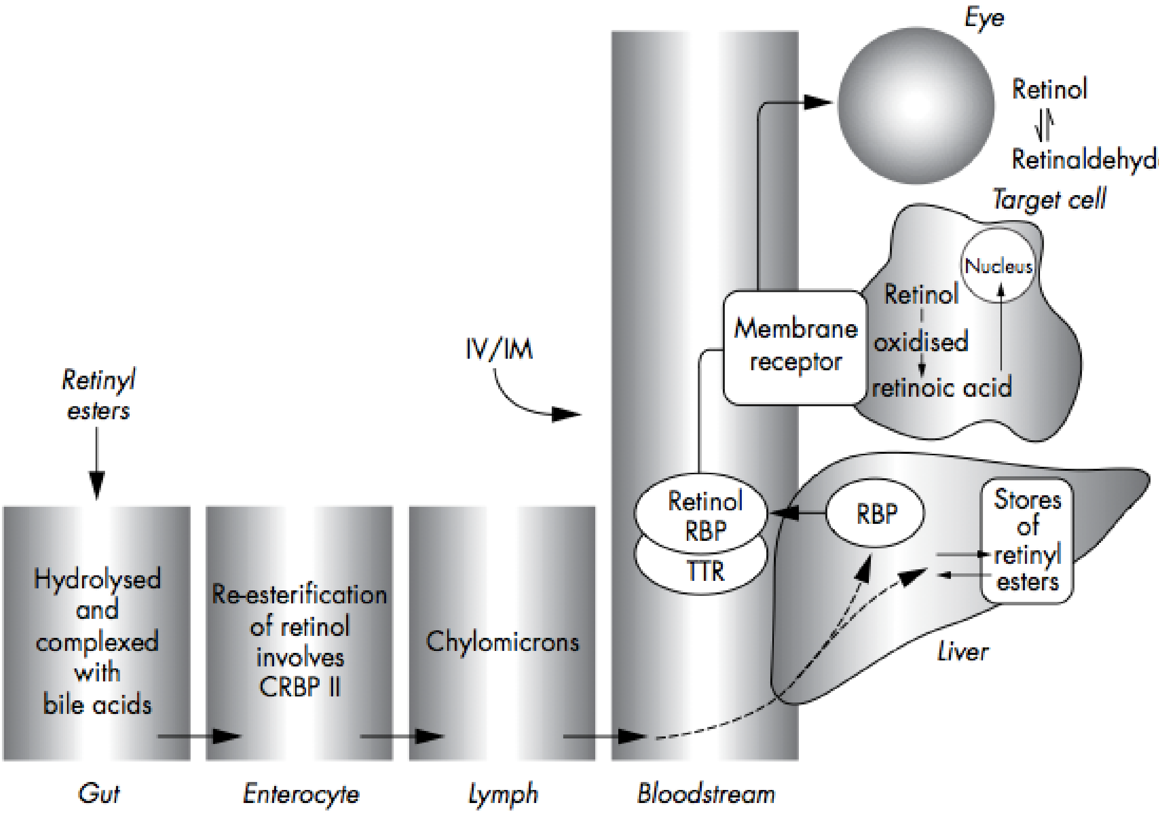

Biological effects of vitamin A in the preterm infant

Vitamin A is required in the fetal lung for both cellular differentiation and surfactant synthesis [18]. In particular, VA stores in the form of RE appear to be of crucial significance as is evident through their quick consumption during the crucial phase of lung development. In the rat, significant storage of VA in the lungs occurs in the third trimester. These stores are rapidly depleted during late pregnancy and the early weeks of postnatal life as the lungs grow and develop [14, 19]. Vitamin A and steroid hormones have similar effects on prenatal and postnatal lung development, operate through similar cell receptors, and may be interdependent [17]. The pathological changes of chronic lung disease (CLD) are similar to those observed in VA deficient experimental animals [20]. Plasma retinol concentrations were lower and hepatic stores less in preterm infants who developed BPD, supporting the hypothesis that VAD contributes to the development of chronic lung disease and/or respiratory tract infections in this population. ELBW infants have low plasma concentrations of VA, [21, 22] and VAD predisposes these infants to BPD (Brandt RB et al., 1978). Randomized controlled trials and a systematic review indicate VA supplementation given intramuscularly (i.m.) to decrease BPD or death [23-26] and therefore VA therapy represents a potentially important advance in the therapy of this condition. A recent large, multicenter, randomized, blinded trial reported intramuscular VA supplementation (5000 IU three times a week for 4 weeks) to slightly reduce the risk of BPD at 36 weeks PMA or death before 36 weeks. In this trial 55% in the VA group versus 62% in the control group died or developed BPD by 36 weeks PMA (relative risk, 0.89; 95% CI, 0.80 to 0.99; P=0.03, n=807, number needed to treat: 1:14.3).

Further effects of vitamin A
Low plasma VA concentrations have been associated in some studies with the development of retinopathy of prematurity (ROP) [12, 15], and abnormal conjunctival impression cytology has been associated with ROP requiring treatment. Pooled data show a non-significant trend towards a reduction in ROP in VA supplemented infants [24]. The pathogenesis of ROP is complex and includes free radical mediated oxidative damage to the developing retina, which could in theory be ameliorated by the antioxidant properties of VA. ROP adversely affects the developing photoreceptors; whether this is influenced by the availability of VA and/or rhodopsin is unknown. The incidence of threshold ROP in a group of ELBW infants who received 10000 IU of intramuscular VA three times a week was nil, compared with 16% in those who received half this dose. Although this result did not achieve statistical significance, it suggests that higher doses of VA than those associated with improved respiratory outcome in preterm infants may have a beneficial effect on the incidence of ROP.
Moreover, in a recent clinical trial Mactier et al. could demonstrate that i.m. VA supplementation improves retinal function in susceptible preterm neonates [27].

Neurological Outcome

Several studies have demonstrated that BPD is significantly associated with long-term NDI [4, 6, 28]. Vitamin A may have direct effects on neurodevelopment. Retinoic acid, the active metabolite of VA, is essential for postnatal maturation of the cerebral cortex in animal models [29] and for development of the hindbrain and the visual and auditory systems [30]. Even in the mature brain, retinoic acid signaling has been localized to sites of functional or structural plasticity in the central nervous system. Vitamin A deficiency, which is common in ELBW infants, may therefore contribute to neurologic sequelae.
Alternatively, VA supplementation may influence NDI indirectly through respiratory effects, perhaps by attenuating lung inflammation or cytokine release. Vitamin A is also required for innate and adaptive immunity [31, 32], and it is possible that VAD may increase the risk of sepsis or lead to lung and brain injury by accentuation of inflammatory processes and consequent cytokine release.

1.2 Trial rationale

Bronchopulmonary dysplasia develops as a consequence of prematurity and progressive lung inflammation in infants with specific risk factors.

The long-term health consequences of BPD include respiratory disease that can persist into adulthood and increased susceptibility to respiratory infections, asthma, repeated hospitalizations, NDI, and also increased mortality.

Therapy of BPD, however, remains controversial as outlined above. In this clinical trial we aim to assess one therapeutic option, high dose oral VA supplementation, which is currently intensely debated in the scientific community.

Rationale for patient population
Because of the ELBW infants' early exposure to a number of different BPD risk factors [33], such as premature birth, IUGR or lung immaturity, they constitute the group of infants who are at the greatest risk for developing BPD. In particular, infants ≤28 weeks gestational ages are affected [34] as they are also commonly ≤1000 g of weight. Extremely low birth weight infants will often require positive pressure ventilation (PPV) or positive pressure support (PPS) or supplemental oxygen therapy after birth due to their lung immaturity. These interventions themselves constitute highly relevant risk factors for the development of BPD [33].

Rationale for trial design
In order to investigate the hypothesis that high dose oral VA therapy reduces the risk of BPD or death at 36+0 weeks (36 weeks and zero days) postmenstrual age (PMA) or at date of discharge to home,

whichever comes first, when compared to standard therapy, a prospective, multicenter, double blind, placebo-controlled, two-arm parallel group phase 3 trial with a group sequential design seems appropriate.

A multi-center trial is necessary due to the small cohort of subjects usually encountered in one center. Based on our sample size calculations, we realistically will need at least 25 trial sites with recruitment of infants over a 3-year trial period. Moreover, external validity of trial results is limited in a single center trial.

A current neonatology guideline [35] recommends to apply trial designs that minimize the size of a clinical trial. Therefore we chose a group sequential design with one interim analysis. The chance to demonstrate early superiority of a treatment group with respect to the primary endpoint was considered more important for this trial than the chance to prematurely stop the trial for futility. Consequently, we chose a two-sided test strategy without futility stop.

The required patient number is based on evidence reviewed in a recent Cochrane review [36] and an original trial [37] which showed a trend towards risk reduction when treating ELBW infants with VA but showed no statistically significant result – in part because of an inadequate number of enrolled infants.

All endpoints selected for this trial are common, routine clinical variables used in most clinical trials with regard to BPD, [5, 38] and are also recommended in respective guidelines [35].

The primary endpoint, BPD (moderate/severe) or death at 36+0 weeks PMA or at date of discharge to home, whichever comes first, is a well-established endpoint in neonatal studies, and BPD is used frequently as a marker of respiratory morbidity in this patient cohort [5].

The choices of secondary endpoints selected for this trial are observational variables to assess efficacy, safety and tolerability of VA supplementation in the cohort of ELBW infants. They include variables of development of respiratory function, variables of serum VA status to assess the effect of VA supplementation and known complications and sequelae of prematurity.

The justification for trial duration until 36 weeks and zero days (36+0 weeks) PMA or date of discharge to home, whichever comes first is based on the diagnosis of BPD, which is made/defined by assessment of pulmonary function at 36+0 weeks PMA or at date of discharge to home, whichever comes first.

Rationale for follow-up

Given the long-term consequences of premature birth and BPD, it is crucial not only to assess potential, short-term benefits of high-dose oral vitamin A supplementation on pulmonary function, but also to evaluate medium- and long-term pulmonary and neurological outcome as demonstrated in a number of studies and nicely summarized in a recent meta-analysis (*JAMA. 2009;302:1418-20. Childhood course of lung function in survivors of bronchopulmonary dysplasia. Filippone M, Bonetto G, Cherubin E, Carraro S, Baraldi E; Understanding the Short and Long-term Respiratory Outcomes of Prematurity and Bronchopulmonary Dysplasia.; Am J Respir Crit Care Med. 2015;192:134-56. Understanding the Short- and Long-Term Respiratory Outcomes of Prematurity and Bronchopulmonary Dysplasia. Islam JY, Keller RL, Aschner JL, Hartert TV, Moore PE; Arch Dis Child Fetal Neonatal Ed, 2010.* ***96****(1): p. F20-9. Does bronchopulmonary dysplasia contribute to the occurrence of cerebral palsy among infants born before 28 weeks of gestation?* Van Marter, L.J., et al.). Thus, given the medium- and long-term effects of BPD on both lung function and neurological outcome, it is imperative to enroll our patient cohort into a standardized follow-up program as laid out by the Gemeinsamer Bundesausschuss (GBA) (39). The rationale with regard to the necessity of medium- and long-term follow-up is derived from and based on the key findings of the aforementioned review study.

At these follow-up visits - at 12 months and 24 months corrected age (CA) - specific data with regard to prior hospitalizations and pulmonary infections as detailed above will be collected.
Moreover, the results from the Bayley-II or Bayley III-test at 24 months CA will be used to evaluate and monitor neurological outcome. Because of the complexity of standardized lung function testing in this cohort, we will preferably use a number of surrogate parameters instead of direct lung function tests, i.e. total number of antibiotic treatments as outpatient and total number of hospital admissions both for all causes and for pulmonary infections, respectively. All collected data will be stored in the amended electronic case report form (eCRF) as provided by the IZKS.
Of note, the initial study protocol does not foresee any standardized follow-up visits.
The extension period of the NeoVitaA trial will amount to 2 additional years of study time.
The realization of two standardized follow-up visits in extremely low birth weight infants is in accordance with current German neonatal guidelines (*AWMF-Leitlinien Sozialpädiatrische Nachsorge extrem unreifer Frühgeborener mit einem Geburtsgewicht unter 1000 Gramm; AWMF-Leitlinien-Register Nr. 071-013, Entwicklungsstufe: S2k; Leitlinien-Koordinator: Dr. med. M. Schulz unter Mitarbeit von: • Dr. med. C. Hertzberg; • Prof. Dr. med. C. Roll; • Dr. med. W. Voss; • Dipl. Psych. M. Wachtendorf; • Päd. M.A. A. Weißbrodt; • Prof. Dr. med. B. Wilken*) and will benefit from the fact that these follow-up visits have become routine clinical practice in Germany, thus guaranteeing a very high turn-out rate of patients.

1.3 Treatments and rationale for dose selection

The pathological changes of BPD are similar to those observed in VA deficient experimental animals [20]. Retinol is required in the fetal lung for both cellular differentiation and surfactant synthesis [18], and it is thought that VA induces the formation of alveoli postnatally [42]. This forms the pathophysiological justification for the conclusion that VA therapy is able to help to prevent BPD.

Figure 2: adapted from Massaro and Massaro, 2010 [42].

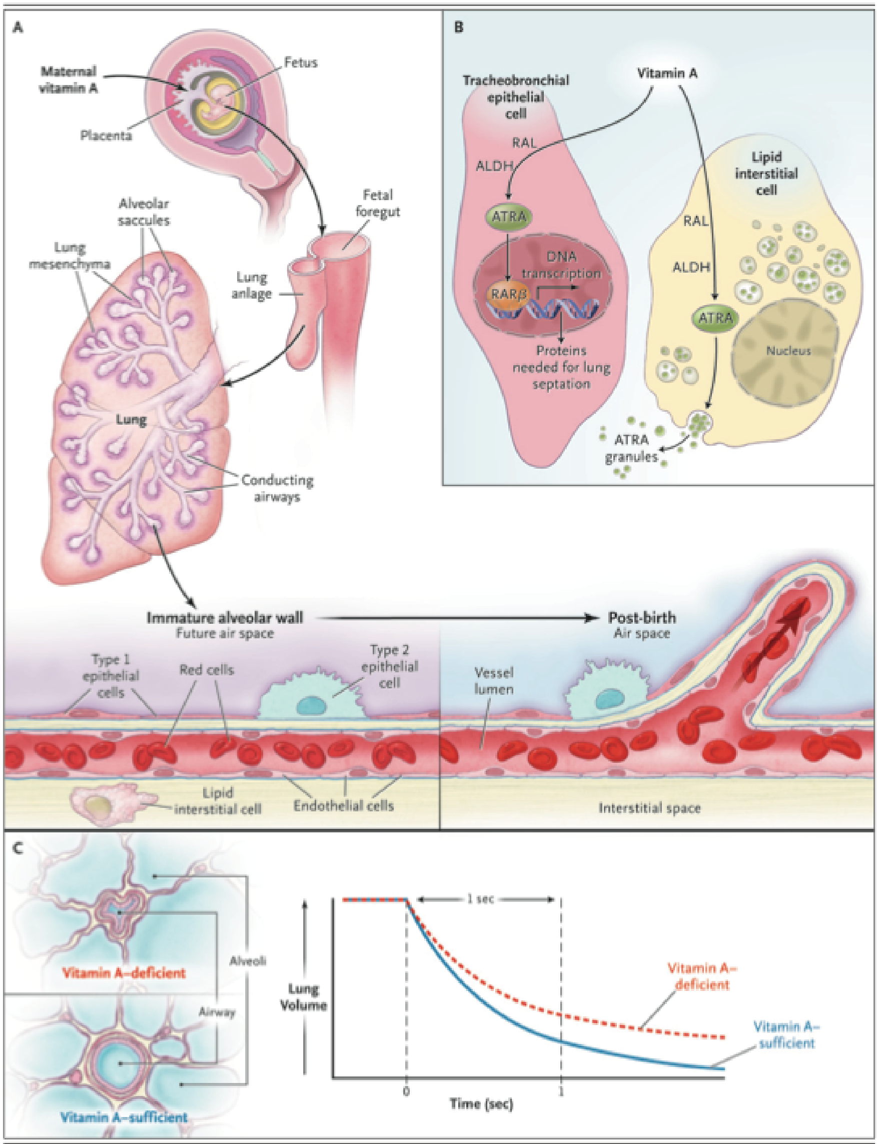

Maternal VA enters the fetal circulation through the placenta (Panel A). In the cytoplasm of tracheobronchial cells of the fetus, it is enzymatically converted to all-*trans* retinoic acid (ATRA), which can then enter the nucleus, where it is bound by retinoic acid receptor β (RARβ). RARβ carrying ATRA can then bind to the retinoic acid response element of DNA, leading to the formation of messenger RNA, which travels to the cytoplasm, where it leads to the formation of a protein critical for lung septation. In the alveolar region of the immature lung, VA enters interstitial fibroblasts (which store retinol), is enzymatically converted into ATRA, and can then be secreted into the interstitial space, from which it can enter other cells of the alveolar wall (e.g., alveolar type 1 and type 2 cells and capillary endothelial cells). In alveolar wall cells, ATRA, acting through RARγ and retinoid X receptor α (RXR α), results in the formation of alveolar septa capable of gas exchange (Panel B). In addition, in lungs with sufficient VA, the alveolar attachments to airways are taut and maintain better airway patency than in lungs that are deficient in VA (Panel C). This could lead to the improved airflow noted by Checkley et al. [43]. ALDH denotes aldehyde dehydrogenase, and RAL retinal aldehyde dehydrogenase.

Early supplementary VA is usually given to ELBW infants intravenously (i.v.) using a multivitamin preparation (in addition to vitamin D, E, and K) as long as the neonate is on parenteral nutrition (PN), normally between one and three weeks after birth. The recommended dose of approximately 500-1000 IU/kg/day [44] is equivalent to the approximate daily retinol intake of a term, breast-fed infant of a well-nourished mother during the first week of life. The American Society for Clinical Nutrition recommends 910 IU/kg/d as the minimum dose suitable for preterm infants and has suggested evaluation of a higher dose [45]. European guidelines currently recommend a maximum intake of

enteral VA of 3300 IU/kg BW [46]. Enteral VA supplementation usually consists of enteral nutrition containing VA and VA supplements.

Moreover, RCTs and a systematic review also support the notion that VA supplementation (mainly when given i.m.) is beneficial in decreasing BPD or death [23-26].

Unfortunately, i.m. VA injections are painful and have to be repeated several times. Therefore, this practice has not been widely accepted in neonatal intensive care medicine [47].

Due to the widespread non-acceptance of repeated i.m. injection, in our opinion, there is a clear necessity to investigate whether additional high dose oral VA supplementation may be beneficial in this patient cohort.

Early oral VA supplementation as part of individual unit protocols and in addition to standard intravenous supplementation is widespread clinical practice. However, data regarding its efficacy and benefit is lacking, and doses may still be too low [37].

This topic has been examined before in a trial by Wardle et al [37]. The authors examined the oral application of 5000 IU/kg VA versus placebo administered over an oro-gastric tube (OGT) over 28 days. This trial showed a positive trend, however failed to show a difference in the incidence of CLD between the treatment and placebo group. They conclude that a further trial of higher dose oral supplementation may be useful.

Scrutinizing the trial by Wardle et al., we believe that there were some significant methodological concerns, and the trial was not adequately powered.

Based on the above stated, we propose a new trial in which we now intend to increase the dosage of postnatal oral VA supplementation from 5000 IU/kg to 6000 IU/kg. In the “verum group”, this will be achieved by providing additional 5000 IU/kg to 1000 IU/kg baseline therapy. This dose will be given for 28 days, commencing within 3 days after birth, and is about 20% higher than the dose used in the Wardle trial.

The dose chosen in the intervention group is a fairly conservative maximum dose as to date. Even within carefully controlled clinical trials utilizing doses of up to 5000 IU/kg/day, there has been neither evidence of acute VA toxicity in this population nor any evidence of long-term adverse effects. Of note, there is no published evidence to suggest that parenteral supplemental of VA in preterm infants in doses of up to 8500 IU/kg/day is associated with significant side effects [11].

In preterm infants, pharmacokinetics are complex and individual variation is likely to be large [11], a view that is shared by renowned experts in the field (Prof. Biesalski, University Hohenheim, Stuttgart, Germany; personal communication).

The endogenous VA status of ELBW infants is also dependent on various factors, including gestational age, maternal VA status as well as the use of antenatal steroids. It is known that corticosteroids, whether given antenatally or postnatally significantly increase plasma retinol concentrations in preterm infants [12, 48-50]. This may be due to depletion of hepatic stores as demonstrated in the rat model [51].

The assessment of body stores may therefore be more important than the assessment of circulating concentrations of retinol [11, 52].

The “adequate” concentration of plasma VA in very low birth weight infants is not known. Serum concentrations <0.7 µmol/L have been considered to indicate VAD in premature infants, concentrations <0.35 µmol/L severe deficiency and depleted liver stores. Normal values for children (and adults) older than 6 months of age is ~1.0 – 2.8 µmol/L [53].

Further, it is unclear whether peak or trough plasma retinol levels are more important in relation to the overall VA status [54] and plasma levels are closely coupled to the availability of RBP, which is known to be low in premature infants [22, 55].

Hence, in terms of pharmacokinetics, a low plasma concentration of VA is not necessarily indicating low absorption, but may indicate immediate distribution from the blood to empty storage sites (liver, lung). Efficacy of the investigated therapy may therefore not be adequately defined through biochemical markers of VA status (retinol, RBP, RE).

Nevertheless, their monitoring may help to show associations between therapy responders and non-responders as well as, in case toxicity concerns are raised, to show associations between plasma levels and potential toxicity. Retinol, as a marker of effective VA supplementation, has also been widely used in various other clinical trials [12, 26, 37, 56] and therefore is also used in this clinical trial for comparison reasons. It has been accepted that RE and RBP reflect VA body stores and evaluation of those may help to determine the VA status of an infant.

Supplementing VA by the enteral route in VLBW infants rather than the parenteral route seems not to be as effective when given in the same dosage [57], however when comparing 5000 IU given enterally to 2000 IU given i.m. the biochemical markers of assumed VA sufficiency (retinol, RBP, RDR test) after supplementation corrected an assumed deficiency and were similar at the end-point assessment [58]. The decision to supplement 6.000 IU/kg/d is based on above findings and on the trial by Wardle et al. [37], who have demonstrated a positive trend in reducing BPD using 5000 IU/kg/d oral supplementation without experiencing significant side effects.

As trial medication we will be using Vitadral® drops - an already registered oral VA product approved for treatment of VAD and use in children <1 year in a dose of between 4000 – 6000 IU/day. In this product, VA is solved in peanut oil. This product will be compared to a placebo containing pure, refined peanut oil.

The rationale for the treatment period of 28 days is based on the pathophysiology of the development of BPD: minimal exposure to noxious factors may affect the normal processes of pulmonary microvascular growth and alveolarization during the crucial time of initial lung development in the cohort of ELBW infants [33]. It is during this time period that the developmental arrest or delay takes place (see also Figure 3).

Early VA supplementation is considered to be most beneficial, whereby the duration of 28 days was also previously used in a similar trial [37].

Figure 3: adapted from Baraldi and Filippone, 2007 [33].

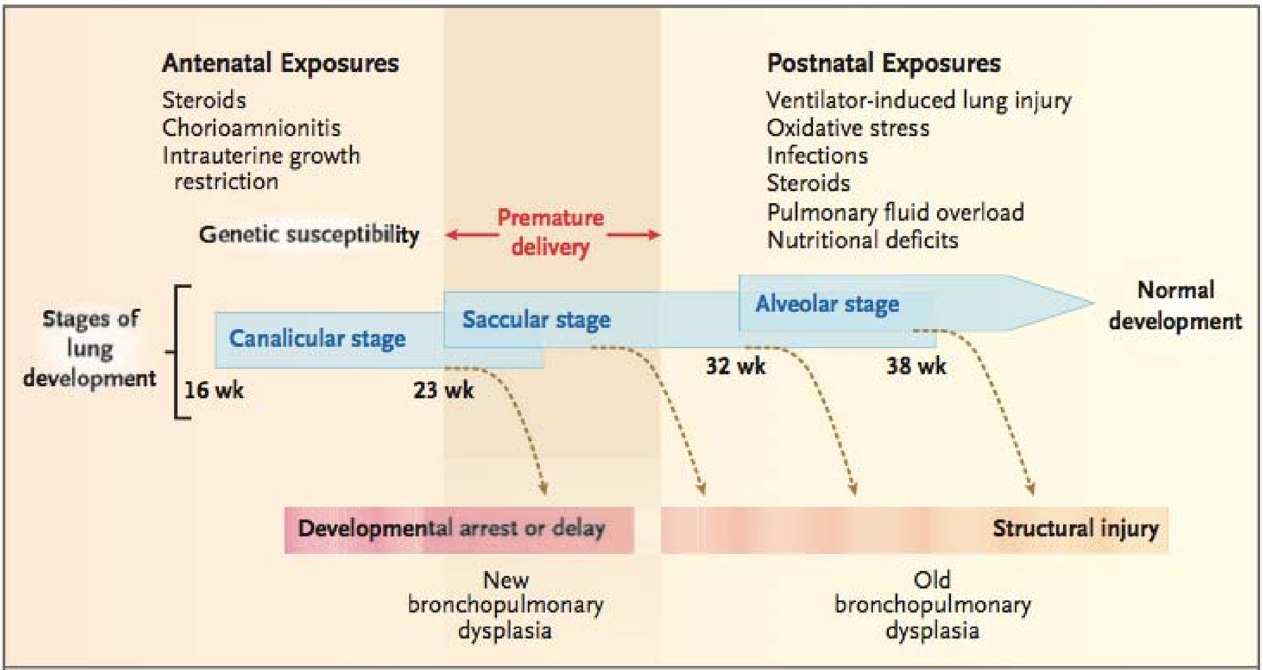

Figure 1. Stages of Lung Development, Potentially Damaging Factors, and Types of Lung Injury.
In premature newborns, the lungs are often exposed to several sources of injury, both before and after birth. Such exposures — as well as genetic susceptibility to problematic lung development — may cause direct airway and parenchymal damage and induce a deviation from the normal developmental path. Depending on the timing and extent of the exposures, lung injury may range from early developmental arrest (new bronchopulmonary dysplasia) to structural damage of a relatively immature lung (old bronchopulmonary dysplasia). Premature infants born at a gestational age of 23 to 30 weeks (region shaded light red) — during the canalicular and saccular stages of lung development — are at the greatest risk for bronchopulmonary dysplasia.

1.4 Summarized risk benefit assessment

Administration of the maximum of 5000 IU/kg (plus 1000 IU/kg baseline) VA supplementation therapy administered orally over 28 days of life is assumed to be efficient and safe for the following reason:

Preterm infants are known to be born with inadequate body stores of VA [11]. High doses of VA are thus assumed to replete endogenous body stores during the first 4 weeks of life, thus maintaining adequate plasma concentrations during the crucial phase of lung development. Furthermore, in the trial by Wardle et al., using oral VA supplementation, no difference was found in the incidence of possible adverse events between the high dose VA group and the standard VA group. More importantly, there was no increased incidence of seizures, vomiting, or NEC in the intervention group. They included a total of 154 neonates <27 PMA with a birth weight of <1000 g. A daily, single dose of 5000 IU/kg of oral VA was given for 28 days. Concomitant therapy included 233 IU/kg/d VA intravenously while on PN [37]. A number of other relevant studies have reported their side effect profile, however, without showing any evidence of significant side effects [25, 26, 37, 54, 59-62].

However, the treatment risks are those potential risks as associated with the administration of peanut oil and a high dose of VA in the form of retinyl palmitate.

Side effects may be experienced following an acute or chronic overdose and are as described in the Vitadral® Product leaflet. There is the theoretical risk of causing an acute anaphylactic reaction using peanut oil; however, to the best of our knowledge, no such effect has been described in the literature

and is unlikely due to immune system immaturity. A short overview of the potential side effects, as detailed in the Vitadral® Drug leaflet, is given in Table 1: Potential side effects of Vitadral® Therapy.

Table 1: Potential side effects of Vitadral® Therapy

Acute Side effects	Side effects on laboratory markers	Chronic side effects
Headaches, lethargy, nausea, papilledema, nycturia, irritability, anorexia, weight loss, slight increased body temperature, subcutaneous swelling and tinnitus, excessive exfoliation of the skin (after 24 hours) and bulging fontanelle.	Acute:<br>Increases in the fibrinolysis time, increased INR, increased ALT and AST.<br><br>Symptoms resolve after 36 hours. Very rarely anemia, thrombocytopenia.	Early symptoms:<br>Dried, scaling or itchy skin, perioral lesions, hair growth impairment, tiredness, bone pain, hemorrhagic events.
	Chronic:<br>Increases of serum alkaline phosphate and serum calcium.	Late symptoms:<br>Hepatosplenomegaly, hypertrophy of liver fatty tissue, fibrosis of the liver, hepatic central vein sclerosis, liver cirrhosis following portal hypertension and ascites, pseudotumor cerebri through increase of cerebrospinal liquor production, early closure of the epiphysis and enlargement of the cortical region of long bones resulting in skeletal growth impairment.

The above described side effects and risks will be carefully monitored using clinical examinations and laboratory and imaging investigations.

Clinical examinations will be performed twice daily to look for signs and symptoms of VA toxicity. This will not pose an additional significant stressor for the infant.

Additional blood tests will coincide with regular clinical blood examinations so that no additional painful procedures are required. Cranial ultrasound examinations (CUSS) are already scheduled as part of the clinical routine.

Laboratory investigations for safety will consist of 4 trial specific sampling procedures. Trial specific blood tests for safety analysis will be performed on treatment days 4 (± 1 day), 12 (± 2 days), 19 (± 2 days) and 26 (± 2 days). The additional blood volume collected during the trial specific tests for safety will not exceed 2.4 ml (0.3 ml maximum each for serum and EDTA blood sample container per sampling procedure).

Due to the potential Vitadral side effects on laboratory markers, (including reported changes in ALT, AST, CHE, ALP, serum calcium and creatinine) anemia and thrombocytopenia, we consider close monitoring of liver, renal and hematological function during the above mentioned time periods crucial for safety reasons. Blood tests for safety analysis require a minimal blood volume of 0.3 ml per sampling container for technical reasons. Although changes in the clotting cascade have been rarely reported, we believe that risk-benefit assessment favors the avoidance of additional coagulation screening which would require further blood samples.

Blood tests for investigation of VA status (retinol, RE, retinol binding protein) will be performed on each patient at three different times: at birth (on cord blood), 20-28 hours after the last application of the trial medication and at 36+0 weeks PMA or at date of discharge to home, whichever comes first.

Due to the unforeseen circumstances of premature birth it may not always be possible to include all suitable subjects prior to birth and therefore investigations of VA status in some individuals may not include blood samples at birth.

The additional blood volume required for analysis of VA status will be two drops, each approximately 50 µL of blood, from a heel prick or venous blood sample, equalizing approximately 100 µL, per sampling procedure. This will then be a total of maximum 200 µL or 0.2 ml (cord blood is excluded from the calculation) additional blood volume for VA status analysis from each patient over the trial duration.

The total trial related blood loss (trial specific blood tests for safety analysis and VA status analysis) will therefore be approximately 2.6 ml over the trial duration until 36+0 weeks PMA or date of discharge to home, whichever comes first (approximation of trial duration: 7-12 weeks) and approximately 2.5 ml over a 4 week period. Due to the importance of the blood tests for safety analysis of the investigational therapy we consider this blood volume as justified.

According to the respective guideline [63] the above mentioned interventions pose no or only a minimal risk to trial subjects.

The investigational product will be administered enterally using either an OGT or naso-gastric tube (NGT). As part of their clinical routine, it is common practice for any neonate to have either an OGT or NGT in situ and hence no additional procedure is required.

We have established a data safety and monitoring board (DSMB).The DSMB will supervise the conduct of this trial and will issue recommendations for early termination, modifications or continuation of the trial according to the DSMB operating procedure.

In case of adverse events, additional scans as well as additional examinations and blood sampling procedures as part of the usual clinical routine may be necessary for safety evaluation.

The development of BPD in preterm neonates constitutes a severe clinical problem with an increased risk of re-hospitalization, mainly due to secondary life-threatening acute respiratory infections during early childhood, and long-term impairment in both respiratory function and neurodevelopmental outcome including cerebral palsy [6, 64-71]. So far, most interventions to reduce the rate of BPD have been ineffective (e.g. caffeine, and other methylxantines, nitric oxide via inhalation), or have been associated with negative long-term sequelae (PNS).

As outlined above, we conclude from the published data that the treatment benefit for the individual subject included in the trial is a potential reduction in the risk of developing BPD or death.

Moreover, if we are able to prove a beneficial effect of VA supplementation in the susceptible cohort, VA supplementation therapy may be adopted as standard treatment for all ELBW infants in the prevention or treatment of BPD, which would have significant implications not only for the individuals involved but could also result in cost reduction in health care systems.

In conclusion, a positive risk benefit ratio exists for this clinical trial as the trial interventions are unlikely to cause a significant burden to the trial subjects, and the trial investigational product may reduce the risk to the individual and the population of ELBW infants to develop BPD, which is associated with considerable morbidity and mortality.

We consider the risks and inconveniences associated with the investigational therapy and specific trial procedures as acceptable and limited due an intense clinical laboratory and sonographic surveillance.

2 TRIAL OBJECTIVES

The primary aim of the trial is to investigate the influence of early postnatal additional high dose oral VA supplementation in ELBW infants on the incidence of BPD or death.

We also aim to investigate the safety and tolerability of additional high dose oral VA supplementation and to assess its efficacy on the development of respiratory function and on known complications and sequelae of prematurity. Moreover, pulmonary and neurological assessments will be performed at 12 and 24 months (CA) follow-up outpatient visits.

Obtaining plasma retinol levels in a subgroup of subjects is a further objective and will give indications about adequacy of VA supplementation (efficacy of supplementation).

The trial objectives, as they are outlined below, will be assessed at their respective endpoints, which are detailed in chapter 3.3.

2.1 Primary objective

The primary objective of the trial is to investigate whether early postnatal additional high dose oral VA supplementation (5000 IU/kg body weight/day) for 28 days reduces the absolute risk of BPD (moderate/severe) or death at 36+0 weeks PMA or at date of discharge to home, whichever comes first in ELBW infants when compared to placebo treatment.

2.2 Secondary objectives

As secondary objectives, the effect of high dose oral VA supplementation on important neonatal (ELBW infant population) development such as respiratory function and on known complications and sequelae of prematurity will be assessed.

Secondary objectives of the trial are:

1. All-cause mortality
2. All grade BPD (mild/moderate/severe)
3. Duration of PPV and PPS
4. Duration of evolving BPD since birth
5. Serum VA status
6. Retinopathy of prematurity (ROP)
7. Intraventricular hemorrhage (IVH)
8. Periventricular leukomalacia (PVL)
9. Necrotizing enterocolitis (NEC)
10. Safety and tolerability of trial medication
11. Pulmonary assessment at 12 and 24 months corrected gestational age
12. Neurological assessment at 24 months corrected gestational age
13. Anthropometric data

These objectives also follow recommendations as outlined in the “Guideline on the investigation of medicinal products in the term and preterm neonate” [35] and further guidance as requested by the German Federal Institute for Drugs and Medical Devices.

Further details about the efficacy assessments can be found in chapter 6.1 (Assessment of efficacy).

3 TRIAL DESIGN

This is a prospective, multicenter, double blind, placebo-controlled, two-arm parallel group phase 3 trial to evaluate the effect of early postnatal additional high dose oral VA supplementation of 5000 IU/kg/d versus placebo for 28 days in preventing BPD or death in ELBW infants.

There will be two groups, a “verum” and a “placebo” group, with 457 subjects in each group.
Trial subjects are ELBW premature neonates whose parents have given *Informed Consent* and who comply with the trial’s inclusion and exclusion criteria (chapter 3.5.1 and 3.5.2).

After confirmation of subjects’ eligibility for trial inclusion, they will be randomly assigned to either the treatment with the investigational medicinal product (IMP) or with the placebo for 28 days and assessed until 36+0 weeks PMA or date of discharge to home, whichever comes first (Figure 4: Trial Flow Chart).

Figure 4: Trial Flow Chart

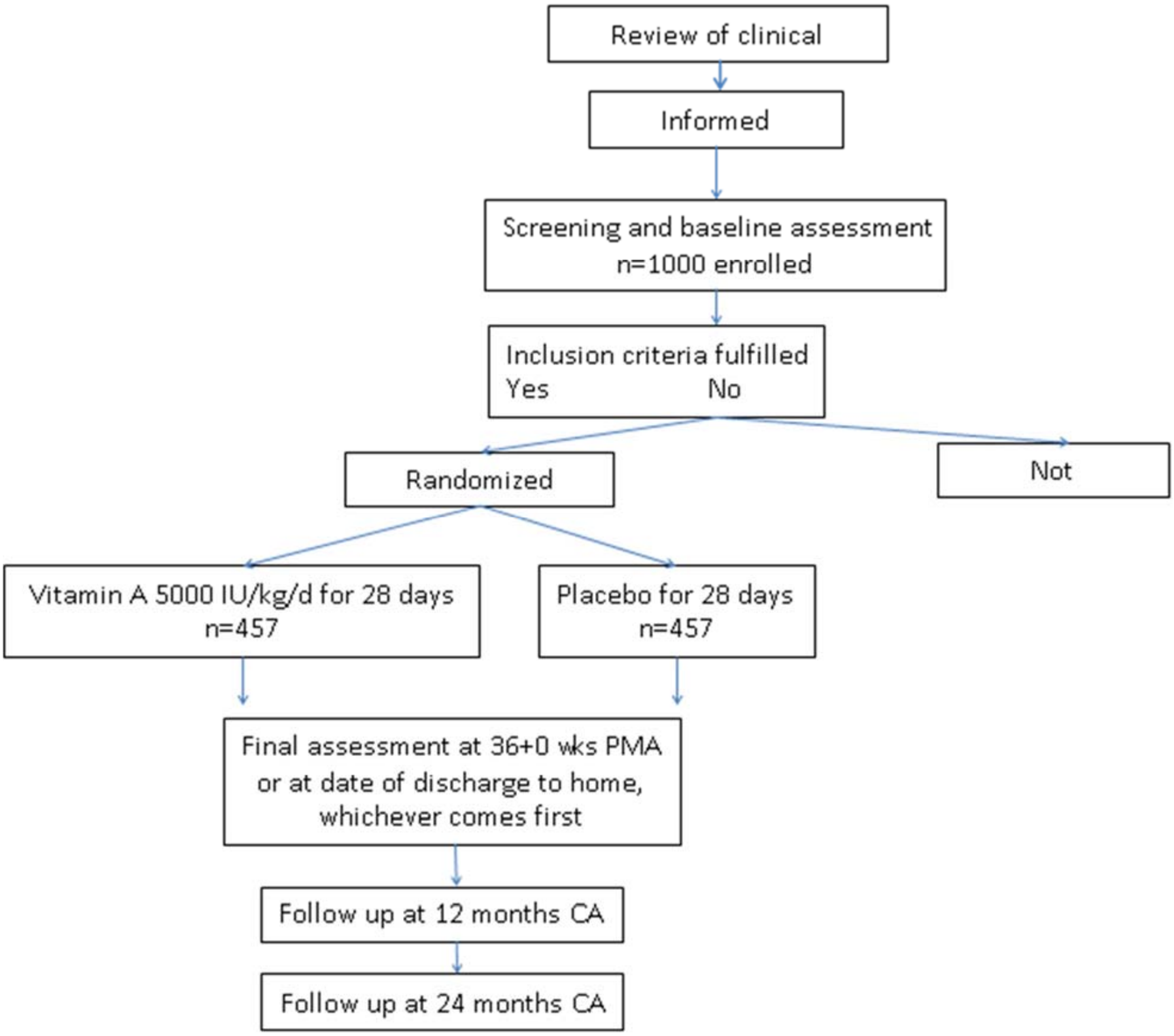

Detailed description of the randomization procedures are outlined in chapter 3.4. Trial treatment is outlined in chapter 4.

3.1 Trial duration and schedule

The overall duration of this trial is planned to be 72 months. Subject recruitment is scheduled to start in Q1 2015 and end in Q4 2020. The last visit for the last subject (end of trial) is scheduled for Q2 2021. The last 24 month follow up visit will be expected by Q2 2023.

Subjects will participate in the trial once included. Trial inclusion, randomization and application of first trial medication must be accomplished until 72 hours after birth.

Details of the complete trial schedule including the different trial visits and specific detailed actions as well as a description of examinations at each visit are outlined in chapter 5. Figure 5 details the timeline of the trial.

Figure 5: Timeline of the trial

**Preterm Birth**			**28 days**	**at 36+0 weeks PMA or at date of discharge to home, whichever comes first**	**at 12 months**	**at 24 months**
antenatal	postnatal	up to 72 hours of life			Follow up 12 months corrected age	Follow up 24 months corrected age
Review of medical records and consent procedures, Cord blood sampling		Eligibility check and randomization	Treatment with trial medication	Trial termination and final assessment	Clinical outcome of pulmonary function	Clinical outcome of pulmonary function and neuro-logical development

3.2 Number of subjects and centers

914 subjects must be randomized, i.e. 457 subjects per treatment group. Calculating a screening failure rate of around 10%, approximately 1000 subjects will be screened in order to enroll 914 subjects in this clinical trial.

Recruitment and treatment of subjects is expected to take place in more than 25 trial centers in Germany and Austria and Switzerland.

3.3 Primary and secondary endpoints

All endpoints used in this trial are common and routine clinical variables that are used in most clinical trials with regard to BPD [5, 38].
Since assessment of pulmonary function with standardized pulmonary function tests is not feasible at this age, a number of surrogate parameters – as detailed below – will be employed.
Conversely, neurological assessment can be done using standardized test batteries at corrected age of 24 months (Bayley-II or Bayley-III-scales).
Details, methods and timing for uniform recording and assessment of all efficacy and safety variables throughout all trial sites are outlined in chapter 5 and 6.

3.3.1 Primary endpoint

The primary endpoint of the trial is the combined incidence of BPD (moderate/severe) or death at 36+0 weeks PMA or at date of discharge to home, whichever comes first.

The primary endpoint, BPD or death at 36+0 weeks PMA or at date of discharge to home, whichever comes first, is a well-established endpoint in neonatal studies, and BPD is used frequently as a marker of respiratory morbidity in this patient cohort. Further, this is a commonly selected primary compound endpoint in neonatal studies investigating any therapeutic modality in BPD [5, 38].

3.3.2 Secondary endpoints

Efficacy, safety and tolerability of the interventional therapy are monitored through clinical examination and observation, laboratory tests and imaging investigations.

The secondary endpoints of the trial are:

1. Any death
2. Any occurrence of BPD (mild/moderate/severe)
3. Days of PPV or PPS
4. Days of evolving BPD
5. Serum retinol levels, RBP, RE
6. Diagnosis of ROP
7. Diagnosis of all grades of IVH
8. Diagnosis of PVL
9. Diagnosis of NEC
10. Occurrence of any adverse event
11. Total number of antibiotic treatments at 12 and 24 months of c.a.
12. Total number of antibiotic treatments for pulmonary infections at 12 and 24 months of c.a.
13. Total number of hospital admissions at 12 and 24 months of c.a.
14. Total number of hospital admissions for pulmonary infections at 12 and 24 months of c.a.
15. Results of Bayley-II or Bayley III scale at 24 months of c.a.
16. Anthropometric data at 12 and 24 months of c.a.
17. Neurological and non-neurological diseases during the first 12 and 24 months c.a.
18. Use of supportive therapies and specific drug treatment

For investigation of the effect of VA supplementation on the development of respiratory function, any occurrence of all forms, including the mild form, of BPD, as well as the days spent on PPV or PPS, or days of evolving BPD will be assessed as these are routinely used and generally accepted variables for clinical monitoring.

VA supplementation effect on the development of known complications of prematurity including ROP, IVH, PVL and NEC will be closely monitored and assessed for safety and tolerability reasons.

3.4 Measures taken to minimize/avoid bias

3.4.1 Randomization

Bias is minimized by randomized treatment allocation based on a randomization list generated at IZKS Mainz by means of a SAS program. Randomization will be stratified by trial site. Block randomization with variable block size will be applied.

The randomization list will be kept in safe and confidential custody at IZKS Mainz.
A copy of the randomization list will be provided to the pharmacy of the University Medical Centre Mainz for packaging double-blind trial medication. A patient receives the lowest available medication number within the correct stratum and the corresponding medication package.

3.4.2 Blinding

The trial will be performed in a double-blind fashion. Subjects and all staff involved in the conduct and/or analysis of the trial will remain blinded with regard to the randomized treatment until breaking the blind after database lock. Blinding of the investigator is achieved by providing verum and matching placebo oral drops in identical dark glass bottles of 30 ml.

In addition to the trial medication the investigator will receive a set of sealed envelopes, one for each medication number. An identical set of sealed envelopes will be held at the pharmacy of the University Medical Centre Mainz. These envelopes contain information on the subject's trial medication and are to be opened only under circumstances in which it is medically imperative for diagnostic or therapeutic decisions to know what the subject is receiving. Date and reason for opening of a sealed envelope must be documented by the investigator or an authorized person on the open envelope, on the CRF and on the medical record of the subject. In either case before unblinding, the investigator must try to consult the coordinating investigator (LKP). The randomization envelopes are not to be opened by the investigator at the end of the trial. All envelopes will be collected by the monitor at the end of the trial.

3.5 Selection and withdrawal of subjects

The trial population of this trial comprises ELBW infants being treated in a neonatal intensive care unit (NICU) who are at high risk for BPD, whose parents or legal representatives have given *Informed Consent*, and who comply with the inclusion and exclusion criteria listed below. No subject will be allowed to enroll in this trial more than once.

Eligibility will be checked by assessment of inclusion and exclusion criteria.

3.5.1 Inclusion criteria

Subjects meeting all of the following criteria will be considered for trial inclusion:

- Neonates with a birth weight <1000 g
- Neonates with a gestational age < 32+0 weeks at time of inclusion into the trial
- Who receive any oxygen supplementation or respiratory support after admission to the NICU within 72 hours following birth
- Who (will) receive a basic VA supplementation of 1000 IU/kg body weight/day (7000 IU/kg body weight/week)
- Postnatal age <72 hours of life
- Minimal/enteral feeds (≥2.5 / <10 ml/kg body weight per day) commenced
- Signed and dated informed parental or legal representative's consent

3.5.2 Exclusion criteria

Subjects presenting with any of the following criteria will not be enrolled in the trial:

- ≥1 major congenital abnormalities
- Congenital nonbacterial infection with overt signs at birth
- Terminal illness as evidenced by pH <7.0 for >2 hours or persistent bradycardia (heart rate <100 bpm) associated with hypoxia for >2 hours.
- Birth weight <400 g
- Participation in another clinical trial according to German Drug Law (AMG)

3.5.3 Required during the trial

All subjects in this trial will receive baseline VA supplementation of 1000 IU/kg VA for the treatment period, either intravenously as multivitamin preparation in addition to their PN or, when enteral feeds are commenced, orally.

Therefore, all subjects in this trial must

- be provided with baseline intravenous VA supplementation of 1000 IU/kg/day through a multivitamin preparation added to their PN for the duration the infant receives PN

and

- receive baseline oral VA supplementation of 1000 IU/kg once PN is discontinued and enteral feeds have been commenced until treatment day 28

3.5.4 Withdrawal criteria

Parents or legal representatives can withdraw their consent at any time without giving any specific reasons. This must be without disadvantages for the subject. However the investigator should try to perform a final visit to get concluding findings of the investigation.

In addition, subjects may be withdrawn from the trial for the following reasons

- At the instigation of the Sponsor
- If, in the investigator’s opinion, continuation of the trial would be detrimental to the subject’s well-being
- If the trial site is prematurely closed

The Principal investigator decides about withdrawal of subjects from the clinical trial in case of occurrence of criteria mentioned above. The investigator will inform the Sponsor as soon as feasible about the decision. In all cases, the reason for withdrawal must be recorded in the CRF and the subject’s medical notes with date and time of the decision.

In case of withdrawal at the request of the parents or legal representative, all reasons should be explored and documented in the CRF as detailed as possible. The subject must be observed further and as far as possible, all examinations, assessments and records as outlined in chapter 5 under “End of trial visit” should be performed and documented as complete as possible.

All ongoing serious adverse events of withdrawn subjects have to be followed up until no more signs and symptoms are verifiable or the health condition of the subject has stabilized but no longer than 30 days after the subject’s discontinuation from the trial.

Withdrawn subjects will not be replaced.

3.5.5 Premature closure of trial sites

Trial sites may be prematurely closed due to the following reasons:

- major protocol violations

- violations of legal and ethical regulations (GCP)
- poor recruitment (<5 subjects/year) or no subjects recruited during 6 months
- non-compliance of investigator

3.5.6 Premature closure of the clinical trial

For the following reasons the whole trial may be discontinued at the discretion of the Sponsor:

- New risks for subjects become known
- Inefficacy of the trial medication becomes evident
- Occurrence of AEs unknown to date in respect of their nature, severity, and duration or the unexpected increase in the incidence of known AEs
- Medical or ethical reasons negatively affecting the continued performance of the trial
- Difficulties in the recruitment of subjects

The ethics committees (EC) and the competent authorities must then be informed. Should the trial be closed prematurely, all trial material (completed, partially completed, blank CRF, randomization envelopes, investigational medicinal products, etc.) must be returned to IZKS Mainz, Germany or collected by the monitor.

4 TRIAL TREATMENTS

4.1 Investigational Medicinal Product

4.1.1 General information about investigational medicinal product (IMP)

Investigational Product

Brand Name:	Vitadral® (54900 IU/ml) oral drops
Drug code:	3000071.00.00
International nonproprietary name (INN):	Retinyl Palmitate
Pharmaceutical form:	Oral solution
Manufacturer:	Aristo Pharma
Dosage approved (in Germany):	Two to three drops per day in children younger than 1 year of age

Comparative Product (Placebo)

Generic Name:	Placebo (peanut oil = Oleum arachidis) oral drops
Pharmaceutical form:	Oral solution
Manufacturer:	Pharmacy department, University Medical Center of the Johannes Gutenberg-University Mainz, Germany

In this multicenter trial the investigational medicinal product is VA provided as Vitadral® drops. Vitadral® drops contain retinyl palmitate. The volume of 1.0 ml contains 30.2 mg retinyl palmitate, equivalent to 54900 IU. A detailed description of Vitadral® drops can be found in the drug property leaflet which is added to this protocol as a separate document. The comparator trial medication is peanut oil (Placebo oral drops). Vitamin A and placebo (oral drops) will be provided in bottles that contain 30 ml.

4.1.2 Therapeutic effects

Vitadral® drops are indicated for the therapy of VAD that cannot be counterbalanced by nutrition alone. Vitadral® drops may therefore correct a state of VAD.

Vitamin A is required in the fetal lung for both cellular differentiation and surfactant synthesis.

4.1.3 Known side effects

Known side effects of peanut oil (contained both in Vitadral® drops and placebo):

- Peanut oil can cause severe allergic reactions in rare cases

Known side effects of VA include:

Acute overdose symptoms:

- Headaches, lethargy, nausea, papilledema, nycturia, irritability, anorexia, weight loss, slight increased body temperature, subcutaneous swelling and tinnitus, excessive exfoliation of the skin, bulging fontanelle

Acute overdose effects on laboratory parameters:

- Increase in the fibrinolysis time, increased INR, increased ALT and AST. Symptoms resolve after 36 hours. Very rarely occur anemia, thrombocytopenia

Chronic overdose symptoms:

- Early symptoms: dried, scaling or itchy skin, perioral lesions, hair growth impairment, tiredness, bone pain, hemorrhagic events
- Late symptoms: hepatosplenomegaly, hypertrophy of liver fatty tissue, fibrosis of the liver, hepatic central vein sclerosis, liver cirrhosis following portal hypertension and ascites, pseudotumor cerebri through increase of cerebrospinal liquor production, early closure of the epiphysis and enlargement of the cortical region of long bones resulting in skeletal growth impairment

Chronic overdose effects on laboratory parameters

- Increase of serum alkaline phosphate (ALP) and serum calcium

4.1.4 Dosage schedule and administration of the trial drug

All subjects randomized to the trial have commenced minimal enteral feeds ($\geq$2.5 / <10 ml/kg body weight per day) and will start treatment with the trial medication within 72 hours after birth. Subjects will receive either an additional provision of 5000 IU/kg/d of VA supplementation or placebo (peanut oil) enterally for a period of 28 days.

We chose this particular starting point for the trial because premature ELBW infants are known to have low retinol plasma concentrations at birth [21, 22] and early VA supplementation may be supportive for lung development during this crucial time of early lung formation [52].

Trial medication will be provided in bottles that contain 30 ml VA or placebo (oral drops). 1 ml (about 27 Drops) of Vitadral® contains 30.2 mg Retinol palmitate (corresponding to 54900 IU); one drop contains 2033 IU.

The appropriate dose will be prescribed in advance for one week. It has to be calculated as weekly required amount and then distributed evenly over 7 days using complete drops.

Therefore the following calculation has to be performed:

- Calculation of weekly required amount of trial medication
    - Body weight (kg) x 5000 IU x 7 = weekly required additional VA supplementation (IU)
    - Weekly required VA supplementation divided by 2033 IU = required drops/week

- The number of required drops per week is thereafter evenly distributed over 7 days, using full drops only. Rounding is performed for practical purposes as required

The calculation of the weekly dosage/dose of trial medication and prescription of trial medication drops for 7 days will be documented in the CRF and on the clinical drug chart in advance for every week. For correct dosing per body weight, trial subjects have to be weighed once every week.

Birth weight will be the basis for the initial calculation of the weekly required dosage of VA. Thereafter the weekly measured new body weight will be used for further weekly drug calculations.
The IMP will be given by nursing staff as drops added to the infants' enteral feeds once daily with the first feed after 8 am in the morning. The applied dose of trial medication has to be documented in the CRF. Overall duration of the interventional therapy will be 28 days.

An exception to the "8 am rule" may be the first administration of the IMP. Initial enteral feeds may be introduced during midday, afternoon or evening feeds. If this is the case, drops will be added to the infants' enteral feeds during the time, when the first feed was commenced.

Depending on clinical reasoning, time of IMP administration may vary at the discretion of the investigator.

4.1.5 Treatment assignment

The trial medication will be administered only to subjects included in this trial. Assignment of trial treatment follows the randomization list.

Subjects withdrawn from the trial retain their identification codes (e.g. medication number). New subjects will always receive a new identification code.

4.1.6 Treatment after the end of the trial

After the end of the trial, subjects will be treated according to the local unit protocol.

4.1.7 Overdose instructions

There is no rescue medication or specific antidote treatment concerning a VA overdose. If overdose with Vitadral® drops occurs, treatment should be symptomatic and supportive.

As soon as a suspected overdose becomes known, supportive measures should be initiated and further management should be discussed between the investigator and the Sponsor as soon as possible.

Supportive measures as outlined in the Vitadral drug leaflet are not all applicable for the ELBW infant population (e.g. induced vomiting, laxative agents). In case of an overdose, gastric emptying via oro-/nasogastric tube may be considered as well as the use of emollients for itchy, dry skin if indicated.

4.1.8 Emergency procedures or treatment, rescue medication

During and following a subject's participation in the trial, the investigator should ensure that adequate medical care is provided to a subject for any AEs including clinically significant laboratory values.

According to the Vitadral Drug leaflet, peanut oil may very rarely cause severe allergic reactions.

In case of an anaphylactic reaction, standard protocols for the emergency treatment of anaphylaxis should be applied [72].

If, during clinical assessment, concerning features for NEC are detected, ongoing management should follow nationally agreed guidelines [73]. In addition to the management suggested in the relevant guideline, the investigator should test the stool of the subject for occult blood.

4.1.9 Packaging and labeling

The trial medication, placebo oral drops, will be packed and labeled by Pharmacy department, University Medical Center of the Johannes Gutenberg-University Mainz according to the randomization list and German drug law. Peanut oil as placebo will be packed in dark glass bottles 30ml with vertical dropper and polypropylene screw cap. The contents of the IMP Vitadral® will not be modified by the manufacturing process. The IMP will be relabeled; no further component will be added and no further action will be conducted.

The trial medication will be labeled according to §5 GCP regulation. The label contains the following details:

- For use in clinical trial only
- Name and address of the Sponsor
- Name and dosage of investigational medicinal product (see above)
- Batch number „Ch.-B. or code number", date of manufacturing
- Administration form (drops)
- Content specified by weight, volume or quantity
- Application form (po)
- Dosage instructions
- Expiry date using note "usable until"
- Storage conditions
- Keep away from children
- Medication number
- Protocol ID,
- EudraCT Number (if not contained in an accompanying document handed out to subjects)

According to §5, chapter 8 GCP-V for approved drugs, that are intended for use in clinical trials without additional manufacture arrangements, all required details may be listed in an accompanying document according to §5, chapter 1.

Additional statements will be printed on the label(s) as required by local regulations.

4.1.10 Drug storage, supplies and accountability

The trial medication (Vitadral® drops and Placebo drops) will be provided, labeled and delivered to the trial centers by the Pharmacy department, Universitätsmedizin der Johannes Gutenberg-Universität Mainz, Germany. The Pharmacy department provides the certificate of analysis for the trial medication. The batch numbers of the drugs used in the trial will be given in the trial report.

The investigator will take inventory and acknowledge the receipt of all shipments of the trial medication. All trial medication must be kept in a locked area with access restricted to designated trial staff. The investigators will be responsible for proper storage of the trial medication at each center until dispensation to the subjects.

The trial medication (unopened bottles) must be stored dry and in accordance with the manufacturer's instructions at 2 °C – 8 °C. Opened bottles have to be stored at room temperature and used within 8 weeks.

The investigator will also keep accurate records of the quantities of trial medication dispensed, used, and returned for each subject on the drug accountability form.

The site monitor will periodically check the supplies of trial medication held by the investigator to verify the correct accountability of all trial medication used. At discretion of the sponsor trial medication which has been returned and released by the monitor may be destroyed by the investigator.

It will be assured that a final drug accountability report is prepared and maintained by the investigator.

4.1.11 Procedures for monitoring treatment compliance

Trial medication will be given to the subjects by nursing staff according to the calculated weekly dose prescribed in the CRF and on the drug chart (see 4.1.4).

After application, nursing staff will document the number of drops given in the CRF and on the drug chart.

Compliance will be assessed by the investigator through review of the applied amount of drops of trial medication recorded in the CRF and the content of bottles.

Details will be recorded in the CRF and on the drug accountability form in the investigator site file.

5 Trial Schedule

The complete trial schedule includes, a baseline visit, daily visits during treatment with trial medication, daily visits after treatment with trial medication and an end of trial visit at 36+0 weeks PMA or at date of discharge to home, whichever comes first. At 12 and 24 months corrected gestational age follow-up visits are scheduled to observe clinical long-term outcome. Figure 6 details the trial schedule.

Details on the assessment and recording of below-mentioned proceedings are provided in Chapter 6.

Figure 6: Trial schedule

**Type of Visits**	**Baseline**	**Treatment**	**Post-Treatment**	**End of trial**	**FU 1**	**FU 2**
**Time of Visits**	**Within 72 hours of life**	**27 Treatment Days**	**Post-Treatment**	**36+0 weeks/ discharge**	**12 months corrected age**	**24 months corrected age**
**Assessments**						
Parental informed consent	X					
Subject trial inclusion	X					
Maternal medical records	X (b)					
Neonatal medical records	X	X (b)	X (b)	X (b)		
Inclusion/exclusio n criteria	X					
Randomization	X					
Calculation of the required amount of trial medication	X	X				
Application of trial medication	X (c)	X (d)				
Assessment of evolving BPD	X (a)	X	X	X		
Assessment of BPD				X		
Assessment of clinical condition(e)	X-(a)	X (f)	X (g)	X		
Assessment of complications and sequelae of prematurity (h)	X	X	X	X		
Assessment of growth (i)	X	X	X	X		

Nutritional assessment	X (a)	X	X	X		
Blood tests for VA status(k)	X		X	X		
Blood tests for safety		X (l)				
CUSS examination	X (m)	X (m, n)		X (n)		
Adverse events	X	X	X	X		
End-of-trial assessments				X		
Anthropometric data (o)					X	X
Neurological and non-neurological diseases					X	X
Physical therapy and Frühförderung					X	X
Use of drugs					X	X
Pulmonary assessment (p)					X	X
Neurological assessment (q)						X

(a) In case the baseline visit occurs one, two or three days after birth clinical assessments which were performed during this time should also be documented in the eCRF.

(b) Review and recording of concomitant diseases and concomitant treatment.

(c) First application of trial medication can be anytime during the day, but should take place with the enteral feed following randomization.

(d) Any subsequent application of trial medication should be in the morning with the first feed after 8:00 am if possible. However, a delay of at least 12 hours between the first and second application should be respected, even if that means, that the second application must be delayed until the second or third feed after 8:00 am on the second trial day.

(e) If bulging fontanelle was found on assessment of clinical condition, an extraordinary CUSS examination including PW-duplex/Doppler sonography should be performed and results documented in the eCRF.

(f) Assessment of clinical condition during drug treatment: Twice daily.

(g) Assessment of clinical condition post treatment: Once daily.

(h) IVH/PVL will be evaluated during CUSS examinations performed according to the outlined schedule above (see also 6.3.2). NEC screening using clinical examination for assessment of clinical condition will be performed once at baseline, twice daily during treatment days and once daily afterwards. ROP screening will take place according to the ophthalmological screening schedule (see also 6.3.2).

(i) Growth parameters will be measured at birth, once weekly thereafter and at 36+0 weeks PMA or at date of discharge to home, whichever comes first.

(k) For investigation of VA status the first blood sample should be taken from (venous)/arterial cord blood at birth; alternatively venous or arterial blood prior to first application of Vit A Subsequent samples must be obtained 20-28 hours after the last application of the trial medication and at 36+0 weeks PMA or at date of discharge to home, whichever comes first (± 2 days) and can be either venous, arterial or capillary samples.

(l) Trial specific blood tests for safety analysis will be performed on treatment days 4 (± 3 days), 12 (± 3 days), 19 (± 3 days) and 26 (± 3 days).

(m) Baseline CUSS examination may be performed (once between day one and three of life).

(n) Second CUSS examination will be performed on day 7 (± 3 days) of life, a third at treatment day 28 (± 7 days). The last CUSS examination will be at 36+0 weeks PMA or at date of discharge to home, whichever comes first (± 7 days).

(o) Anthropometric data: body weight, length, head circumference, failure to thrive

(p) Pulmonary assessment: total number of antibiotic treatments as outpatient and total number of hospital admissions both for all causes and for pulmonary infections, respectively

(q) Neurological assessment: Bayley II or Bayley III-scale (MDI, PDI)

Details on the assessment and recording of below-mentioned proceedings are provided in Chapter 6.

5.1 Review of clinical records and *Informed Consent* procedures

To facilitate timely enrollment in the trial, most suitable subjects will be identified prior to trial inclusion through review of maternal medical records before birth. However, due to the unpredictable nature of premature birth, suitable subjects may also be identified after birth through review of maternal and neonatal medical records.

As soon as a potential trial subject has been identified, the subject's parents or legal representatives will receive complete information about the trial, both orally and in writing.

At birth a cord blood sample will be taken in all potential trial subjects for potential analysis of VA status. However these blood samples will only be sent for laboratory analysis if informed consent from either parents or legal representatives has been obtained and the subject is eligible for trial participation.

A written "*Informed Consent*" will be obtained from both parents or the legal representatives in accordance with GCP and the local legislation for each subject prior to initiating any trial-specific procedure and cord blood analysis for VA status.

Thereafter, subjects will be assigned a subject-ID, which must be recorded on the Screening-Log.

5.2 Baseline visit

After obtaining *Informed Consent* and after birth the baseline visit will be performed within 72 hours of life to facilitate the enrolment of the subject. During this visit all trial inclusion and exclusion criteria will be assessed, and, if compliance with all criteria is confirmed, the subject will be enrolled in the trial and documented in the eCRF. Finally the subject will be randomized and assigned a medication number (see chapter 3.4.1).

The subject's randomization must be completed within 72 hours after birth. Treatment allocation will be performed as outlined in chapter 4.1.5. Once randomization is completed, the first dose of trial medication will be applied.

During the baseline visit the following steps will be carried out:

- Blood tests
  - VA status (cord blood sample at birth), if applicable; alternatively venous, capillary or arterial blood prior to first application of Vit A
- Review of maternal medical records and documentation of
  - Demographics
  - Medical history including obstetric history
  - Previous and concomitant diseases

  - Previous and concomitant treatment
- Review of neonatal medical records and documentation of the subject's
  - Demographics
  - Birth history
  - Previous and concomitant diseases
  - Previous and concomitant treatment
- Review of neonatal medical records and documentation of clinical examination/assessment (from birth until randomization)
  - Assessment of evolving BPD (once daily)
  - Assessment of clinical condition (once daily)
  - Assessment of complications and sequelae of prematurity
    - NEC assessment (once daily)
    - IVH/PVL (according to CUSS examination schedule (day 1-3 of life))
- Nutritional assessment (from birth until randomization)
- Assessment of growth (at birth)
- Baseline CUSS examination – may alternatively be performed on day 1-3 of life (see below)
- Confirmation of eligibility
  - Assessment of inclusion and exclusion criteria
- Randomization
- Trial medication
  - Calculation and prescription of the required amount of daily trial medication during the first week
  - Application of first dose of trial medication
- Monitoring of AEs

5.3 Daily visits during trial treatment

During the following 27 days of drug treatment with trial medication, twice-daily trial visits will take place.

Tasks:

- Trial medication
  - Calculation and prescription of the required amount of daily trial medication (once weekly in advance)
  - Application of trial medication (daily, in the morning with the first feed after 8:00 am if possible). A delay of at least 12 hours between first and second application should be respected
- Clinical examination/assessment
  - Assessment of evolving BPD (once daily)
  - Assessment of clinical condition (twice daily)
  - Assessment of complications and sequelae of prematurity
    - NEC assessment (twice daily)
    - IVH/PVL: according to CUSS examination schedule (day 7 of life (± 3 days), treatment day 28 (± 7 days))
- Blood tests
  - Safety (treatment days 4 (± 3 days), 12 (± 3 days), 19 (± 3 days) and 26 (± 3 days)

- Nutritional assessment (once daily)
- Assessment of growth (once weekly)
- CUSS examination: Second CUSS examination will be performed on day 7 (± 3 days) of life, third at treatment day 28 (± 7 days)
- Review and recording of neonatal concomitant diseases and treatments (once daily)
- Monitoring of AEs (daily)

During these trial visits the trial medication should be given in the morning if possible, followed by the clinical examination/assessment. Blood tests should coincide with any regular clinical blood sampling procedures. If needed, a time window (see above) is allowed to allow for congruence of routine and trial specific blood sampling procedures. All other assessments should be performed during the morning round.

5.4 Post-treatment visits

After the trial treatment period has finished once daily trial visits for monitoring of the trial subjects will take place until 35+6 weeks PMA or one day before date of discharge to home, whichever comes first.

Tasks:

- Clinical examination/assessment
  - Assessment of evolving BPD (once daily)
  - Assessment of clinical condition (once daily)
  - Assessment of complications and sequelae of prematurity
    - Assessment for ROP (First ROP examination should be scheduled for the 6th postnatal week (between the 36th and 42nd day of life), however not before 31 weeks PMA see also paragraph 6.3.2)
      Follow-up examinations will be arranged by the treating Pediatric Ophthalmologist usually either weekly or bi-weekly
    - NEC assessment (once daily)
- Blood tests
  - VA status (sample should be obtained 20-28 hours after the last application of the trial medication)
- Nutritional assessment (once daily)
- Assessment of growth (once weekly)
- Review and recording of neonatal concomitant diseases and treatments (once daily)
- Monitoring of AEs

5.5 End of trial visit

At 36+0 weeks PMA or at date of discharge to home, whichever comes first, the end of trial visit will take place.

Tasks:

- Clinical examination/assessment
  - Assessment of evolving BPD
  - Assessment of clinical condition
  - Assessment of complications and sequelae of prematurity
    - IVH/PVL (according to CUSS examination schedule) (± ~~1~~ 7 days)
    - NEC assessment

  - Assessment of BPD
- Blood tests
  - VA status (± 2 days)
- Nutritional assessment
- Assessment of growth
- Review and recording of neonatal concomitant diseases and treatments
- Monitoring of AEs
- End of trial assessments

5.6 Follow-up (FU) Visits

At 12 and 24 months corrected age follow-up visits will be done to observe clinical long-term outcome.

5.6.1 12 month-FU-visit

- Anthropometric data: body weight, length, head circumference, failure to thrive
- Social parameters: single or two-parent household, availability of supportive (family) network
- Neurological and non-neurological diseases
- Physical therapy and Frühförderung
- Use of drugs
- Assessment of pulmonary function: total number of antibiotic treatments as outpatient and total number of hospital admissions both for all causes and for pulmonary infections, respectively

5.6.2 24 month-FU-visit

- Anthropometric data: body weight, length, head circumference, failure to thrive
- Social parameters: single or two-parent household, availability of supportive (family) network
- Neurological and non-neurological diseases
- Physical therapy and Frühförderung
- Use of drugs
- Assessment of pulmonary function: total number of antibiotic treatments as outpatient and total number of hospital admissions both for all causes and for pulmonary infections, respectively
- Assessment of neurological development: Bayley II or Bayley III scale

6 TRIAL METHODS

6.1 Assessment of efficacy

The trial-specific efficacy variables as well as the timing for recording of the findings are detailed below.

6.1.1 Assessment of evolving BPD

For the assessment of evolving BPD the following variables will be assessed daily from trial inclusion until the end of the trial:
In case the baseline visit occurs one, two or three days after birth clinical assessments listed below which were performed during this time should also be documented in the eCRF.

- Application of oxygen supplementation (with FiO_2 of >0.21) >12 hours for respiratory support to maintain SaO_2 ≥92% or SaO_2 <92% >12 hours with or without application of oxygen supplementation
- Application of any respiratory support to maintain SaO_2 ≥92%
- Mode and duration of respiratory support (PPV or PPS), if applicable
- If PPV, mode of PPV (conventional ventilation, HFOV, combination of conventional ventilation and HFOV, other [non-invasive ventilation] e.g. nIPPV / CPAP with frequency
- If PPS, mode of PPS (CPAP or HFNC ≥2l/min flow in oxygen or air)

One day of evolving BPD is defined in this trial as any period of >12 hours in a 24 hour period which the infant spent on >0.21 of inspired fractional oxygen (FiO_2) for respiratory support to maintain oxygen saturations ≥92% as measured by an oxygen saturation probe for at least 5 minutes
or as any period of >12 hours in a 24 hours period which the infant spent on oxygen saturations <92% with or without oxygen supplementation.
Any combination of oxygen supplementation to maintain oxygen saturations ≥92% and saturations <92% with or without oxygen supplementation >12 h (as detailed above) will also count as one day of evolving BPD.
The oxygen reduction test according to Walsh [74] will not be routinely necessary, but may be employed under specific circumstances.

Days of evolving BPD will be counted from birth up to 36+0 weeks PMA or date of discharge to home, whichever comes first (see also 6.1.3).

One day of PPV or PPS is defined in this trial as any duration of the respective respiratory support during a 24 hour period.

Days of PPV or PPS will be counted as BPD days from birth up to 36+0 weeks PMA or date of discharge to home, whichever comes first

6.1.2 Assessment of BPD

The following variables will be assessed for the definition of BPD at 36+0 weeks PMA or at date of discharge to home, whichever comes first.

Variables:

- Days of evolving BPD from birth up to 36+0 weeks PMA or date of discharge to home, whichever comes first
- Breathing room air at 36+0 weeks PMA or at date of discharge to home, whichever comes first
- Amount of oxygen supplementation at 36+0 weeks PMA or at date of discharge to home, whichever comes first, if applicable
  - o FiO_2 of >0.21 and FiO_2 of <0.3 or
  - o FiO_2 ≥0.3
- Respiratory support (PPV or PPS) at 36+0 weeks PMA or at date of discharge to home, whichever comes first

For the purpose of this trial the following definition of BPD will be used:

- Mild BPD: at least 28 days of oxygen supplementation with FiO_2 of >0.21 to maintain SaO_2 ≥92% for respiratory support or SaO_2 <92% >12 hours with or without application of oxygen supplementation

and breathing room air at 36+0 weeks PMA or at date of discharge to home, whichever comes first

- Moderate BPD: at least 28 days of oxygen supplementation with FiO_2 of >0.21 to maintain SaO_2 ≥92% for respiratory support or SaO_2 <92% >12 hours with or without application of oxygen supplementation
  and the additional need for oxygen supplementation with FiO_2 of >0.21 and FiO_2 of <0.3 at 36+0 weeks PMA or at date of discharge to home, whichever comes first
- Severe BPD: at least 28 days of oxygen supplementation with FiO_2 of >0.21 to maintain SaO_2 ≥92% for respiratory support or SaO_2 <92% >12 hours with or without application of oxygen supplementation
  and the need for oxygen supplementation with FiO_2 of ≥0.3 and/or PPV or PPS for respiratory support at 36+0 weeks PMA or at date of discharge to home, whichever comes first

Also, for the purposes of this trial HFNC ≥2l/min is considered positive pressure support (PPS), independent of the concentration of oxygen or air flow [75].

The oxygen reduction test according to Walsh [74] is not routinely necessary for trial purposes, but may be employed under specific circumstances (eg, oxygen supplementation for other purposes (ROP), and for exact quantification of oxygen requirements at 36 p.m.a.

The BPD definition used for this trial is adapted from the NICHD network definition of BPD (Figure 7) according to Jobe and Bancalari [3].

Figure 7: NICHD network definition of BPD; Adapted from Jobe and Bancalari, 2001 [3]. BPD = bronchopulmonary dysplasia, NCPAP = nasal continuous positive airway pressure, PMA = postmenstrual age, PPV = positive-pressure ventilation. Note: Only a small number of ELBW infants (<5%) are expected to be discharged home before 36+0 weeks PMA. * A physiologic test confirming that the oxygen requirement at the assessment time point remains to be defined.

Gestational Age	< 32 wk	≥ 32 wk
Time point of assessment	36 wk PMA or discharge to home, whichever comes first	> 28 d but < 56 d postnatal age or discharge to home, whichever comes first
	Treatment with oxygen > 21% for at least 28 d **plus**	
Mild BPD	Breathing room air at 36 wk PMA or discharge, whichever comes first	Breathing room air by 56 d postnatal age or discharge, whichever comes first
Moderate BPD	Need* for < 30% oxygen at 36 wk PMA or discharge, whichever comes first	Need* for < 30% oxygen at 56 d postnatal age or dis-charge, whichever comes first
Severe BPD	Need* for ≥ 30% oxygen and/or positive pressure, (PPV or NCPAP) at 36 wk PMA or discharge, whichever comes first	Need* for ≥ 30% oxygen and/or positive pressure (PPV or NCPAP) at 56 d postnatal age or discharge, whichever comes first

6.1.3 *End-of-trial assessment*

The following efficacy variable will be assessed at the end of the trail at 36+0 weeks PMA or at date of discharge to home, whichever comes first.

Variable:

- Date of death, if applicable

6.1.4 Assessment of neurological development

Neurological assessment can be done using standardized test batteries at corrected age of 24 months (Bayley-II or Bayley III-scales) (https://www.testzentrale.de/shop/bayley-scales-of-infant-and-toddler-development-third-edition.html; accessed 10.07.2017; Essentials of Bayley Scales of Infant Development II Assessment Maureen M. Black, Kathleen Matula. New York: John Wily, 1999. ISBN 978-0-471-32651-9).

Variable: MDI (mental Development Index), PDI (Psychomotor development Index)
Also, hearing and visual impairment will be assessed.

6.2 Blood tests for VA status

In order to assess safety and efficacy of VA supplementation blood sampling will be performed for VA status analysis in all subjects across all trial sites.

Timing for VA status assessment:

- At birth (venous cord blood), alternatively venous, arterial or capillary blood prior first application
- Post treatment day 1 (20-28 hours after the last application of the trial medication)
- End of trial at 36+0 weeks PMA or at date of discharge to home, whichever comes first (± 2 days)

At birth an arterial cord blood sample should be taken, if available, alternatively venous, arterial or capillary blood prior first application. All subsequent samples may be either arterial, venous or capillary samples. Blood tests for trial purposes should always be combined with any regular clinical blood sampling procedures, if possible.

Blood sampling for VA status assessments will be done using the dried blood spot (filter paper) method [76, 77]. Whatman 903™ filter paper cards will be used.

Labeling of the filter card must be unequivocal and must include the following information:

- NeoVitaA Trial
- Subject ID of the patient
- Sampling date and time
- Sample material (arterial cord blood, arterial, venous or capillary blood)
- Visit name

Per sampling procedure, two blood samples with a volume of ca. 50 µl blood each (approximately one large drop of blood per spot) will be necessary and will subsequently be applied to two circled areas on the filter paper.

Each blood sample applied to the filter paper should nearly completely fill the circled area on the filter paper. The blood spots do not need to expand over the circled area and should be clearly visible at the back of the paper.

After applying the blood to the labeled filter paper card, it must be dried for at least 4 hours (better overnight). During the drying time the filter card must be protected from direct sun light. After complete drying the filter card must be stored protected from light in a plastic bag within the provided envelope at room temperature (between 20-25 degrees Celsius) and sent the next working day to:

Universität Hohenheim
Institut für Biologische Chemie und Ernährungswissenschaft (140A)
NeoVitaA Trial - VA Status Analysis
Garbenstraße 30 (Bio I)
70593 Stuttgart.

Further details on the collection, storage and posting procedures of the DBS samples will be outlined in a separate document and will be provided to the trial sites prior to commencement of the trial.

VA status parameters:

- Serum retinol level
- Serum RBP level
- Serum RE level

For assessment of retinol and RE, a spot of 6 mm cross-sectional diameter will be punched and prepared for HPLC chromatography according to the method published by Craft et al. [76]. The method was modified in order to also measure RE. RBP measurement will be performed according to the method published by Erhardt et al. [78].

6.3 Assessment of safety and tolerability

6.3.1 Assessment of clinical condition

The following variables will be assessed once daily from birth until randomization, twice daily during the treatment period, once daily from the end of the treatment period until the end of the trial. In case the baseline visit occurs one, two or three days after birth clinical examinations listed below which were performed during this time should also be documented in the eCRF. These variables were selected to appropriately assess the clinical condition of the subjects of this trial and to monitor safety and tolerability.

Variables:

- General appearance
- Vigilance (not influenced by medication)
- Perfusion: capillary refill time
- Temperature instability
- Apnea
- Fontanelle
- Vomiting
- Abdominal distension not due to positive pressure support (CPAP/non-invasive ventilation)
- Rectal bleeding
- Bloody stools
- Bilious vomiting

Capillary refill time is assessed at either an arm or at a lower leg. The skin over the testing side is pressed with the ball of the thumb for 3-5 seconds so that it blanches. The thumb is then lifted and the time taken for refilling of the capillaries and a return to original skin color is noted.

Temperature instability is considered when there is a variability of >1 degree Celsius around the baseline temperature of 37.0 degrees Celsius which cannot be explained by environmental factors.

Apnea is defined as the occurrence of no effective respiratory effort in combination with bradycardia (heart rate <80 beats per minute independent of duration) and hypoxemia (defined as SaO_2 <80%).

A bulging fontanelle is an outward curving of an infant's fontanelle, is tense to touch and protruded from the skull. Diagnosis can only be made when the infant is not crying. A bulging fontanelle should prompt an additional cranial ultrasound investigation as outlined in 6.3.4.

Abnormal clinical examination/assessment results, should prompt a further, detailed clinical assessment and must, if the abnormal examination/assessment is clinical significant be documented as concomitant disease or AE.

6.3.2 Assessment of complications and sequelae of prematurity

The following assessments of complications and sequelae of prematurity will be performed at the intervals specified below.

Variables:

- Diagnosis of ROP
- Diagnosis of IVH by CUSS examination
- Diagnosis of PVL by CUSS examination
- Diagnosis of necrotizing enterocolitis (NEC) (Bell stage ≥ IIa)

ROP

All subjects will be screened for ROP by a Pediatric Ophthalmologist according to the guidelines of the "International Committee for the Classification of Retinopathy of Prematurity (ICROP)", revised in 2005 [79].

Timing of screening procedures:

- First ROP examination should be scheduled for the 6th postnatal week (between the 36th and 42nd day of life), however not before 31 weeks PMA [80].

- Follow-up examinations will be arranged by the treating Pediatric Ophthalmologist according to the relevant guidelines, usually either weekly or bi-weekly.

Only severity (stage 1-5; [79]) is used as a parameter for ROP documentation in this trial.

Any stage and change of stage of **ROP** must not be documented on the AE page but is to be documented on the specific page in the eCRF. Documentation of ROP on this eCRF page will be handled as AE documentation. ROP with a fatal outcome must be reported as SAE. For details see chapter 7.

IVH and PVL

All subjects will be screened for IVH, and PVL using cranial ultrasound examinations. Those should be performed as outlined in the DEGUM criteria for standard documentation [81].

Timing for both screening procedures (IVH and PVL)

- Once between days of life 1-3
- On day 7 of life (± 3 days)
- On treatment day 28, and (± 7 days)
- At 36+0 weeks PMA or at date of discharge to home, whichever comes first (± 7 days)

Time window for examinations is plus or minus one day.

IVH grading for documentation in this trial will be according to Papile, 1978 [82]

- Grade I – subependymal hemorrhage
- Grade II – intraventricular hemorrhage without ventricular dilatation
- Grade III – intraventricular hemorrhage with ventricular dilatation
- Grade IV – intraventricular hemorrhage with parenchymal hemorrhage

Any grade and change of grade of **IVH and/or PVL** [83] must not be documented on the AE or concomitant disease page but is to be documented on the specific page in the eCRF. Documentation of IVH on this eCRF page will be handled as concomitant disease or AE documentation, respectively. IVH and/or PVL with a fatal outcome must be reported as SAE. For details see chapter 7.

NEC

All subjects will be screened for NEC using clinical examination for assessment of clinical condition.

Timing of NEC screening procedures

- Once daily from birth until randomization
- Twice daily during trial treatment
- Once daily after trial treatment until 36+0 weeks PMA or date of discharge to home, whichever comes first

Abnormal clinical examination results will prompt further center specific routine care assessments for the evaluation of NEC.

Severity of NEC will be graded according to the modified classification of Bell et al.[84, 85] and as outlined in the relevant guideline [73].

Grading-stages for documentation

- Ia, Ib
- IIa, IIb,
- IIIa, IIIb.

Any grade and change of grade of NEC must not be documented on the AE or concomitant disease page but is to be documented on the specific page in the eCRF. Documentation of NEC on this eCRF page will be handled as concomitant disease or AE documentation, respectively. NEC with a fatal outcome must be reported as SAE. For details see chapter 7.

6.3.3 Blood tests – Safety

Blood tests for safety will be analyzed on trial center site in local laboratories or by radiometer and are regularly scheduled for the following time points:

- At treatment day 4 (± 3 days)
- At treatment day 12 (± 3 days)
- At treatment day 19 (± 3 days)
- At treatment day 26 (± 3 days)

Parameters

- Liver function tests: ALT, AST, CHE (optional if clinically indicated), Bilirubin, ALP
- Renal function tests: Urea (optional if clinically indicated), Creatinine, Sodium, Potassium, Calcium
- Hematological parameters: Hb, WBC, platelets

6.3.4 Cranial Ultrasound Investigations

Screening cranial ultrasound investigations will be performed at the following time points:

- Once between days of life 1-3
- On day 7 of life (± 3 days)
- On treatment day 28, (± 7 days) and
- On day 36+0 weeks PMA or at date of discharge to home, whichever comes first (± 7 days)

If bulging fontanelle was found on assessment of clinical condition, an additional CUSS examination including PW-duplex/Doppler sonography should be performed and results documented in the eCRF. Pathological PW-duplex/Doppler sonography, which is not explained by IVH or by a persistent ductus arteriosus, should prompt a further, detailed clinical assessment and must be reported as concomitant disease or AE.

6.3.5 Adverse events

Adverse events will be reported and assessed as outlined in chapter 7.

6.4 Other assessments

6.4.1 Maternal medical history

Variables:

- Demographics
    - Date of birth
    - Height
    - Weight
- Previous and concomitant diseases
- Previous and concomitant (if breastfeeding) treatments

6.4.2 Maternal obstetric history

Variables:

- Gravida
- Parity
- Singleton or multiple birth
- Expected date of delivery
- Premature rupture of membranes
- Prolonged rupture of membranes
- Premature prolonged rupture of membranes
- Maternal antenatal infections including chorioamnionitis
- Pregnancy induced hypertension
- Maternal pre-eclamptic toxemia
- Maternal smoking during pregnancy
- Maternal recreational drug use

- Other complications of pregnancy

6.4.3 Subject's medical history

Variables:

- Demographics
    - o Date of birth
    - o Ethnicity
    - o Sex
    - o Gestational age
    - o PMA

- Birth History
    - o Mode of delivery
    - o Umbilical arterial pH
    - o APGAR scores (at 1, 5 and 10 minutes)
    - o Any birth complication
- Previous and concomitant diseases
- Previous and concomitant treatment

6.4.4 Growth

The following parameters will be assessed at birth, once weekly thereafter and at 36+0 weeks PMA or at date of discharge to home, whichever comes first.

Parameters:

- Weight
- Length
- Head circumference

6.4.5 Nutritional assessment

The following variables will be assessed daily from trial inclusion until the end of treatment with trial medication:
In case the baseline visit occurs one, two or three days after birth assessments listed below which were performed during this time should also be documented in the eCRF.

- Parenteral nutrition without minimal enteral feeds
- Parenteral nutrition with minimal enteral feeds (≥2.5 / <10 ml/kg body weight per day)
- Parenteral and enteral nutrition (≥10 ml/kg body weight per day)
- Full enteral nutrition
- No nutrition without minimal enteral feeds
- No nutrition with minimal enteral feeds (≥2.5 / <10 ml/kg body weight per day)
- Type of enteral nutrition
    - o Formula
    - o Breast milk
- Oral intake of vitamin A in µg/kg (IU./kg) provided by enteral feeds on day 7, 14, 21 , 28 of life as well as at 36 +0 weeks PMA or at date of discharge to home whichever comes first

6.4.6 End-of-trial assessment

- Checks of regular trial completion

7 SAFETY

7.1 Definitions

Adverse Event (AE)

According to GCP, an adverse event (AE) is defined as any untoward medical occurrence in a patient or clinical investigation subject administered a pharmaceutical product and which does not necessarily have a causal relationship with this treatment. An AE can therefore be any unfavorable and unintended sign (including an abnormal laboratory finding), symptom, or disease temporally associated with the use of a medicinal (investigational) product, whether or not related to the medicinal (investigational) product.

An AE may be:

- A new symptom or a new diagnosis
- A new medical condition or an accident
- A change in laboratory parameters
- Worsening of a medical condition/diseases existing before the start of the clinical trial
- Recurrence of a disease
- An increase in frequency or intensity of episodic diseases.

Surgical procedures themselves are not AEs; they are therapeutic measures for conditions that require surgery. The condition for which the surgery is required may be an AE. Planned surgical measures permitted by the clinical trial protocol and the condition(s) leading to these measures are not AEs, if the condition leading to the measure was present before inclusion in the trial. In the latter case the condition should be reported as medical history.

Change in laboratory parameters: The criteria for determining whether an abnormal test finding should be reported as an adverse event are as follows:

- Test result is associated with accompanying symptoms, and/or
- Test result requires additional diagnostic testing or medical/surgical intervention, and/or
- Test result leads to a change in trial dosing outside of protocol-stipulated dose adjustments, or discontinuation from the trial, significant additional concomitant drug treatment, or other therapy, and/or
- Test result is considered to be an adverse event by the investigator or Sponsor.

Additional to the Adverse Events mentioned above in this clinical trial all new occurrence or worsening of abnormal and clinically significant clinical conditions as defined in 6.3.1, will be documented as AEs (and reported as SAEs, if applicable).

Any grade and change of grade of ROP, IVH, PVL and/or NEC falling in the period of observation of AE must not be documented on the AE page but is to be documented on the specific page in the eCRF. Documentation on these eCRF pages will be handled as AE documentation. If a ROP/IVH/PVL and/or NEC has a fatal outcome within the safety reporting period, then it should be reported as SAE.

BPD is defined and documented as primary endpoint of this trial and therefore BPD and criteria for BPD should not be documented as Adverse Event. If a BPD or symptoms fulfilling criteria for BPD have a fatal outcome within the safety reporting period, then it should be documented as AE and reported as SAE.

Serious adverse event (SAE)

A serious adverse event (SAE) is any untoward medical occurrence that at any dose:

- Results in death
- Is life-threatening
- Requires subject hospitalization or prolongation of existing hospitalization
- Results in persistent or significant disability/incapacity or
- Is a congenital anomaly/birth defect
- Is an important medical event.

Comment:

Death is an outcome of an event. The event that resulted in death should be recorded and reported as SAE.

Life-threatening means that the subject was at immediate risk of death at the time of the serious adverse event; it does not refer to a serious adverse event that hypothetically might have caused death if it were more severe.

If the admission is pre-planned (i.e., elective or scheduled surgery arranged prior to start of the trial) or not associated with an adverse event (e.g., social hospitalization for purpose) or results in a hospital stay less than 12 hours, the serious criterion “hospitalization” is not fulfilled. However, it should be noted that invasive treatment during a hospitalization (less than 12 hours) may fulfil the criteria of “medically important” and may be reportable as a serious adverse event dependent on clinical judgment.

Persistent or significant disability or incapacity” means that there is a substantial disruption of a person’s ability to carry out normal life functions. The irreversible injury of an organ function (e.g., paresis, diabetes, cardiac arrhythmia) fulfils this criterion.

Medical and scientific judgment should be exercised in deciding whether expedited reporting is appropriate in situations where none of the outcomes listed above occurred. Important medical events that may not be immediately life-threatening or result in death or hospitalization but may jeopardize the subject or may require intervention to prevent one of the other outcomes listed in the definition above should also usually be considered serious. A diagnosis of cancer during the course of a treatment should be considered as medically important.

In this trial BPD and criteria for BPD should not be documented as Adverse Event or reported as SAE. Only if BPD or symptoms fulfilling criteria of BPD have a fatal outcome within the safety reporting period, then it should be documented as AE and reported as SAE.

ROP, IVH, PVL and NEC are typical, well known complications of prematurity. All trial subjects will be monitored for these complications according to the routine procedures in the respective trial centre. The new occurrence or worsening of ROP, IVH, PVL and NEC will be closely monitored and assessed for subject safety and tolerability reasons throughout the course of this trial. In addition to the routine examinations, performed in all participating NICUs, the trial schedule defines regular time points for screening of all trial subjects for ROP, IVH, PVL and NEC using adequate examinations. The results of these examinations are documented on specially designed eCRF AE pages for careful safety monitoring.

As the effect of VA supplementation on the development of ROP, IVH, PVL and NEC belongs to the secondary objectives of this trial the occurrence of these complications of prematurity will additionally be evaluated as secondary endpoints. ROP, IVH, PVL and NEC with a fatal outcome within the safety reporting period must be reported as SAE.

Clarification of the onset and end date of AEs and SAEs

The onset date of the AE is defined as the onset of signs and symptoms or a change from baseline. The onset date of the SAE is defined as the date the signs and symptoms/diagnosis became serious, i.e., met at least one of the above stated criteria for seriousness.

The end date of the AE is defined as the date when the symptoms resolve, or the event is considered stable. The end date of the SAE is defined as the time the seriousness criteria are no longer applicable.

The end date of the SAE must not be later than the end date of the corresponding AE.

AEs and SAEs that are ongoing at the time of death are considered not resolved or resolving.

Clarification of the difference in meaning between "serious" and "severe":

The terms “serious” and “severe” are not synonymous but are often used interchangeably. The term ‘severe’ is often used to describe the intensity (severity) of a specific event; the event itself, however, may be of relatively minor significance (such as severe headache). This is not the same as “serious”, which is based on subject/event outcome or action criteria usually associated with events that pose a threat to a subject’s life or functioning. Seriousness (not severity) serves as a guide for defining regulatory reporting obligations.”

Suspected Unexpected Serious Adverse Reaction (SUSAR)

A SUSAR is every SAE with an at least possible relationship to the investigational medicinal product which is unexpected.

An unexpected serious adverse reaction is any serious adverse reaction, the nature or severity of which is not consistent with the applicable product information.

7.2 Assessment of AEs by investigator

Subjects must be carefully monitored for adverse events by the investigator. The seriousness, the intensity of the adverse events and the causal relation to trial medication and/or procedures are to be assessed.

Intensity/Severity

The maximum intensity of an AE will be assessed by the investigator as follows:

Mild: Temporary event which is tolerated well by the subject and does not interfere with normal daily activities.

Moderate: Event which results in discomfort for the subject and impairs his/her normal activity.

Severe: Event which results in substantial impairment of normal activities of subject.

If the event is serious, the severity reported in the adverse event must be consistent with the severity included in the serious adverse event report.

Causal relation to trial medication/procedures

The assessment of the relationship of an adverse event to the administration of trial drug is a clinical decision by the investigator based on all available information at the time of the documentation. Factors to be considered in assessing the relationship of the adverse event to trial drug include:

- The temporal sequence from drug administration: The investigator has to consider, if the event occurred before first intake of trial medication or long time after last intake. Additionally the length of time from drug exposure to event should be evaluated in the clinical context of the event.

• Recovery on discontinuation (de-challenge), recurrence on reintroduction (re-challenge): Subject's response after drug discontinuation (de-challenge) or subjects response after drug re-introduction (re-challenge) should be considered in the view of the usual clinical course of the event in question.

• Underlying, concomitant, intercurrent diseases: Each report should be evaluated in the context of the natural history and course of the disease being treated and any other disease the subject may have.

• Concomitant medication or treatment: The other drugs the subject is taking or the treatment the subject receives should be examined to determine whether any of them may be suspected to cause the event in question.

Positive causal relationship:

An assessment of 'related' implies a reasonable possibility of a causal relationship between the event and the investigational medicinal product (IMP).

This means that there are facts (evidence) or arguments to suggest a causal relationship, such as:

- A close temporal relationship
- A common drug reaction to the IMP
- No plausible alternative cause

Negative causal relationship:

An assessment of 'not related' means that there is no reasonable possibility of a causal relationship between the event and the investigational medicinal product.

This includes for example:

- Another cause of the adverse event is more plausible
- A temporal sequence cannot be established with the onset of the adverse event and administration of the trial treatment
- A causal relationship is considered biologically implausible.

7.3 Period of observation

In this trial, the period of observation for collection of adverse events extends from the time the patient is randomized up to End of trial Visit.

If the investigator detects a serious adverse event (SAE) in a trial patient before or after the end of the period of observation, and considers the event related to trial treatment or trial medication, he should document and report this SAE as detailed /outlined in 7.5.

7.4 Documentation of AEs and Follow-up

All AEs (whether serious or not) detected by the investigator will be documented on the appropriate pages of the case report form (CRF). AEs must also be documented in the subject's medical records. If the adverse event is serious (see Section 7.1), the investigator must complete, in addition to the "Adverse Event Page", a "Serious Adverse Event Form" at the time the serious adverse event is detected.

Every attempt should be made to describe the adverse event in terms of a diagnosis. If a clear diagnosis has been made, individual signs and symptoms will not be recorded unless they represent atypical or extreme manifestations of the diagnosis, in which case they should be reported as separate events. If a clear diagnosis cannot be established, each sign and symptom must be recorded individually.

All subjects who have adverse events, whether considered associated with the use of the investigational products or not, must be monitored to determine the outcome. The clinical course of

the adverse event will be followed up according to accepted standards of medical practice, even after the end of the period of observation, until a satisfactory explanation is found or the investigator considers it medically justifiable to terminate follow-up, but no longer than 28 days after the end of the trial.

Should the adverse event result in death, a full pathologist's report should be supplied, if possible.

7.5 Immediate reporting of SAEs by investigator

SAEs must immediately (within 24 hours of the investigator's awareness) be reported to:

IZKS Mainz
Safety Management
Langenbeckstr. 1
55131 Mainz, Germany
FAX 0049 6131/17-9916

The initial SAE Report should be as complete as possible including the essential details of subject's identification (screening number/random number), the serious adverse event (medical term, diagnosis), the trial medication and the assessment of the causal relationship between the event and the trial medication. The SAE report must be reviewed and signed by the investigator.

The investigator should provide related additional information on the clinical course and the outcome of each SAE as soon as possible (Follow up report).

The "Serious Adverse Event Form" is provided in the Investigator Site File.

7.6 Safety evaluation and reporting by Sponsor

The Sponsor will ensure that all legal reporting requirements are met. According to GCP the Sponsor is responsible for the continuous safety evaluation of the investigational product(s) and the clinical trial.

On behalf of the Sponsor IZKS will conduct the management of SAEs and the expedited reporting as required by applicable Laws and regulations:

in Germany: German Drug Law (AMG) and GCP regulation (GCP-V)

in Austria: Austrian Drug Law (AMG)

Suspected unexpected serious adverse reactions (SUSARs) and safety issues as defined by GCP are determined for expedited reporting: The competent authorities and the ethics committees should be notified as soon as possible but not later than 15 calendar days, and 7 calendar days if it was fatal or life-threatening.

All investigators will be informed, too.

Work flow and procedures concerning Safety management will be described in a separate document.

During the clinical trial the Sponsor will submit the Development Safety Update Report (DSUR) including a list of all serious adverse reactions to the ethics committee(s) and the competent authorities once a year.

A data and safety monitoring committee will be established for this trial (see 11.8).

7.7 Immediate reporting of pregnancy by investigator

Not applicable.

7.8 Documentation of Abuse, Misuse, Overdose and Medication Error

All special events such as trial medication abuse, misuse, overdose and medication errors including dilution errors have to be documented in the subject's CRF and source documents. If any abuse,

misuse, overdose, or medication errors lead to an adverse event, then the event has to be documented and reported as AE/SAE.

7.9 Emergency procedures

During and following a subject's participation in the trial, the investigator should ensure that adequate medical care is provided to a subject for any AEs including clinically significant laboratory values.

7.10 Other safety data

7.10.1 Previous and intercurrent illnesses

Not applicable.

7.10.2 Previous and intercurrent medical treatments

Not applicable.

8 STATISTICS

Details of the statistical analysis of the data collected in this trial will be documented in a Statistical Analysis Plan (SAP) that will be generated by IZKS Mainz and finalized before closing the data base and prior to breaking the blind. The SAP is based on the protocol including all amendments. The document may modify the plans outlined in this protocol; however any major modifications of the primary endpoint definition and/or its analysis will also be reflected in a protocol amendment. Any deviation from the original statistical plan must be described and justified in the final report. The statistical analysis will be conducted by means of SAS®.

8.1 Sample size

Sample size calculation is based on comparison of the primary outcome between the two trial groups with an expected event rate (moderate/severe BPD or death) of 42% in the standard group compared to 33% in the experimental treatment group, thus a clinically relevant difference of 9% in event rates between the two trial groups is expected.

Sample sizes of at least 457 in the "standard treatment group" (placebo) and 457 in the "experimental treatment group" (5000 IU/kg/d) are required to achieve 80% power to detect a difference in the event rates between both treatment groups using a 2-sided χ^2-test with 0.05 significance level. Considering interim analysis after 50% of subjects and multiple testing, these results assume that 2 sequential a priori specified statistical tests are made and the *O'Brien-Fleming α-spending function* is used to determine the test boundaries. No continuity correction was applied and no futility stop is incorporated in this design. Due to the intensive neonatal care until 36+0 weeks PMA or until date of discharge to home, whichever comes first dropouts after randomization are not expected.
The two-sided significance level is 0.0052 for the interim analysis and 0.0480 for the final analysis. 20.96% power of the interim analysis is expected in this design.

Sample size calculation was performed by ADDPLAN 6.0.6, ADDPLAN™ 6. ADDPLAN, Inc., an Aptiv Solutions company.
Although sample size was planned with a χ^2-test, analysis will be performed by a logistic regression analysis with treatment group and trial sites as covariates. Since this approach will presumably reduce unexplained variability, power is expected to increase.
In order to randomize 914 subjects, approximately 1000 potential trial subjects will be screened, assuming a screening failure rate of 10%.

8.2 Analysis populations

All randomized subjects will be included in the Intention-to-treat (ITT) population. Within ITT population analyses subjects will be assigned to the treatment to which they were randomized.

To be eligible for the per protocol (PP) population, subjects must fulfil the following criteria:

- Treatment as randomized
- Treatment compliance of at least 75% measured by drug accountability
- No visits attended out of schedule
- Meeting all inclusion criteria and not meeting any exclusion criteria

The completer population comprises all randomized subjects who suffer BPD (moderate/severe) or death until 36+0 weeks PMA or discharge to home, whichever comes first or complete the trial without event until 36+0 weeks PMA or discharge to home, whichever comes first.

The safety population comprises all subjects who received at least one dose of trial treatment. In analyses of the safety population subjects will be assigned to the treatment which they actually received.

Allocation of all subjects to the analysis populations will be determined prior to database closure.

8.3 Efficacy analyses

The primary population for the analyses of efficacy is the ITT Population. The primary analysis takes place after all patients reached the end of trial visit. The follow up data will be analysed after all follow up data will be collected (assumed to be 24 months later).

8.3.1 Definition and analysis of primary endpoint

Incidence of BPD (moderate/severe) or death at 36+0 weeks PMA or at date of discharge to home, whichever comes first will be compared between treatment groups by a logistic regression analysis with treatment group and trial site as covariates at a global two-sided significance level of 5% (0.52% for interim analysis and 4.8% for final analysis). Trial sites with less than two events in at least one treatment group will be aggregated in order to increase robustness of estimation results. A discrepancy between stratification factors at randomization and covariates in the analysis is thus accepted.
In addition to the confirmatory test in the Intention to treat population, supportive analyses in the Per Protocol Population and the completer population will be performed.
Subjects that drop out after randomization without event (moderate/severe BPD or death) will not be available for the confirmatory analysis.
If at least 2% of randomized subjects are ineligible for the primary analysis, a sensitivity analysis will be performed, in which missing primary endpoint data will be imputed by multiple imputation techniques.

8.3.2 Analysis of secondary endpoints

Evaluation of secondary outcomes is explorative. Descriptive statistical analyses are used for each of the two trial groups separately. For quantitative variables appropriate distributional parameters like mean, standard deviation and quantiles will be provided and explorative two-sample t tests may be performed. For categorical variables absolute and relative frequencies are computed and explorative Fisher tests may be applied.

8.3.3 Analysis of subgroups

For the following variables, subgroup analyses are planned:

- Trial site
- Birth weight < / ≥ 750 g

- Gender
- Antenatal steroid treatment
- Postnatal steroid treatment

Within these analyses, the primary analysis model will be extended by the respective subgroup variable and its interaction with the treatment effect, if the resulting analysis model is capable of properly estimating all effects in the model. Otherwise, separate analyses for all subgroups will be provided.

8.3.4 Interim analyses

One interim analysis is planned after 50% of subjects have reached 36+0 weeks PMA or discharge to home, whichever comes first. An O'Brien-Fleming alpha spending function is used to account for the issue of multiple testing. The two-sided significance level of the interim analysis is 0.0052. A global significance level of 0.05 for the complete test procedure (interim and final analysis) is ensured. Trial sites with less than two events in at least one treatment group will be aggregated for the interim logistic regression analysis (see also 8.3.1).

8.4 Analysis of adverse events

All summaries and listings of safety data will be performed for the safety population.
AEs will be coded according to MedDRA terminology. Detailed information collected for each AE will include: A description of the event, duration, whether the AE was serious, intensity, relationship to trial drug, action taken, clinical outcome. Summary tables will present the number of subjects observed with AEs by MedDRA System Organ Class and Preferred Term and corresponding percentages. Additional subcategories will be based on event intensity and relationship to trial drug. A subject listing of all AEs will be prepared.

8.5 Analysis of clinical laboratory findings

Listings will be prepared for each laboratory measure and will be structured to permit review of the data per subject as they progress on treatment.
Summary tables will be prepared to examine the changes of laboratory measures over time. If required, shift tables will be provided to examine the changes of laboratory data from normal baseline to values outside the corresponding reference range during/after treatment.

8.6 Analysis of Follow-up

All surviving patients from the NeoVitaA trial who present to the scheduled follow-up visits at 12 and 24 months of age (corrected for prematurity). The analysis of the follow-up data will be separate from the primary and secondary outcomes. Although opening the treatment group assignments, the analysis will still be valid. The follow-up data will be collected from social pedagological centers that are not otherwise included in the data collection. Those centers still stay blinded to the treatment allocation. The statistical analysis will be based on the number of all included patients. The number of antibiotic medications and hospital admissions for pulmonary infections and other reasons will be compared by Wilcoxon rank sum tests between treatment groups. Bayley II or Bayley III scores will be analyzed by linear models. The number of important neurological and non-neurological disease will be calculated and compared by Wilcoxon rank sum tests between treatment groups. If indicated, negative binomial or Poisson regression models with time in study as offset variable will be fitted. Social parameters and use of physical therapies will be compared by Chi-square tests and their influence on the development of BPD will be investigated. Anthropometric data will be analyzed by linear models.

9 QUALITY CONTROL AND QUALITY ASSURANCE

The sponsor will continuously manage quality and risks to ensure patient protection and reliability of trial results. The measures used will be proportionate to the inherent risks and the importance of the data collected.

9.1 Requirements for investigator, investigational sites and members of the investigational staff

The investigator should be able to demonstrate (e.g. based on retrospective data) a potential for recruiting the required number of suitable subjects within the agreed recruitment period.

The investigator should be aware of, and should comply with, GCP and the applicable regulatory requirements.

The investigator should have sufficient time to properly conduct and complete the trial within the agreed trial period.

The investigator has to select his investigating team and ensure that all members are adequately qualified, informed about the protocol, any amendments to the protocol, the trial treatments, and their trial-related duties and functions. Furthermore, he must nominate an adequately qualified deputy.

If the investigator/institution retains the services of any individual or party to perform trial-related duties and functions, the investigator/institution should ensure this individual or party is qualified to perform those trial-related duties and functions and should implement procedures to ensure the integrity of the trial-related duties and functions performed and any data generated.

The investigator/institution should maintain adequate and accurate source documents and trial records that include all pertinent observations on each of the site's trial patients.
Source data should be attributable, legible, contemporaneous, original, accurate, and complete. Changes to source data should be traceable, should not obscure the original and should be explained if necessary (e.g., via an audit trail).
When a copy is used to replace an original document, the copy has to fulfill the requirements for certified copies.

Data entered in the CRF directly are listed in the Monitor Manual/ Monitoring Plan in the section source data control.

9.2 Direct entries

Data entries which are directly entered in the eCRF, are listed in the Monitor Manual.

9.3 Direct access to source data/documents

The investigator/institution must permit trial-related monitoring and auditing by appointed staff of the monitoring body, IZKS, as well as inspections by the appropriate competent authorities, providing direct access to source data/documents (Confidentiality see 11.3).

The subjects will be informed that representatives of the Sponsor or competent authorities may inspect their medical records to verify the information collected, and that all personal information made available for inspection will be handled in strictest confidence and in accordance with local data protection laws.

9.4 Investigator site file and archiving

The investigator will be provided with an investigator site file (ISF) at the start of the trial. The investigator will archive all trial data and relevant correspondence in the ISF. The ISF, all source data and all documents will be kept filed according to the requirements of the ICH-GCP guidelines after termination of the trial.

It is the responsibility of the investigator to ensure that the subject-identification sheets are stored for at least 15 years beyond the end of the clinical trial. All original subject files must be stored for the longest possible time permitted by the regulations at the hospital, research institute, or practice in question. If archiving can no longer be maintained at the site, the investigator will notify the Sponsor.

9.5 Monitoring

Monitoring will be conducted according to the SOPs of IZKS Mainz.

On-site monitoring will be done by personal visits from a clinical monitor. To initiate the trial, the monitor will visit all participating local trial sites and trial centers. The monitor shall ensure that the investigators and members of the investigating staff understand all requirements of the protocol and their regulatory responsibilities.

The monitor will ensure that the investigator will maintain a list of members of the investigating staff to whom they have delegated significant trial-related duties ("delegation log").

Each site will be visited by the monitor at regular intervals to ensure compliance with the trial protocol, GCP and legal aspects. The monitor will review the entries into the CRFs for completeness and correctness and verify the entries on the basis of the source documents. The presence of correct informed consents will be checked for every patient. By frequent communications (letters, telephone, fax), the monitor will ensure that the trial is conducted according to the protocol and regulatory requirements.

The investigator must allow the monitor to look at all relevant documents and must provide support at all times to the monitor.

To ensure continuous oversight and risk management by the sponsor between the monitor visits and for preparation of the monitor visits, the monitor or other sponsor staff adequately qualified and trained (e.g. data managers) will check the entries in the (e-)CRFs at regular intervals (centralized monitoring). The intervals and the items to be checked will be specified in the monitoring manual and/or related documents (e.g. data management plan, safety management plan). Centralised monitoring will help to identify erroneous and potentially unreliable data and sites/processes for targeted on-site monitoring]

Details and the rationale for the chosen monitoring strategy will be specified in the monitoring manual for this trial.

9.6 Risk Management

During protocol development and the entire course of the trial, all potential and emerging risks (e.g. delayed CRF data entry) to patient protection and reliability of the trial results will be closely monitored, and preventive and corrective measures will be specified. The sponsor will identify, evaluate, control, communicate, review and report the relevant risks periodically to ensure continuous and timely risk management.

9.7 Measures to secure compliance

Any significant noncompliance with the protocol, SOPs, GCP and regulatory requirements by any party involved in the conduct of the trial will be analysed (e.g. root cause analysis) by the sponsor, and appropriate corrective and preventive actions will be implemented.

9.8 Inspection by authorities

Competent authorities may request access to all source documents, CRF, and other trial documentation in case of an inspection. Direct access to these documents must be guaranteed by the investigator who must provide support at all times for these activities. Source data documents can be copied during inspection provided the identity of the patient have been made unrecognizable.

9.9 Audits

Audits will be conducted at discretion of the sponsor.

10 DATA MANAGEMENT

10.1 Responsibilities

The Data management team is authorized in the case of discrepancies or correction of data errors to contact directly the responsible person at trial site. For the response the investigator has to comply with a term defined by IZKS. The investigator has to agree the contact per e-mail or phone.

A detailed methodology for the data management in this trial will be documented in a data management plan (DMP) that will be dated and maintained by IZKS. This plan has to be signed by the Sponsor, the head of the data management team and the responsible data manager. The document may modify the plans outlined in this protocol; however any major modifications of the data handling will also be reflected in a protocol amendment.

10.2 Data collection

This trial will be performed using an electronic case report form (eCRF) or remote data entry (RDE). The investigator and the trial site staff will receive system documentation, training and support for the use of the eCRF. In the case of new trial site staff the training can be performed by personnel of the trial site.

For support with data entry Data Management at IZKS can be contacted at 06131/17-9920 or 06131/17-9922.

All protocol-required information collected during the trial must be entered by the investigator, or a designated representative in the eCRF. All data entry, modification or deletion will be recorded automatically in an electronic audit trail indicating the individual subject, the original value, the new value, the reason for change, who made the change and time and date of the change. All data changes will be clearly indicated. Former values can be viewed in the audit trail. All electronic data will be entered by the site (including an electronic audit trail) in compliance with applicable record retention regulations.

The system will be secured to prevent unauthorized access to the data or the system. Only people provided with a user ID and a password will be able to enter or change data. The investigator will maintain a list of individuals who are authorized to enter or correct data and their system ID.

Computer hardware and software (for accessing the data) will be maintained at or made available for the site in compliance with applicable regulations. All technical preconditions for each trial site are record in the DMP.

The system is capable of producing exact copies of data in legible paper form for inspections and audits. The investigator or a designated member of the investigational staff, following review of the data in the eCRF, will confirm the validity of each subject’s data by electronic signature or by signing a paper printout of a listing of all subjects enrolled in the trial.

The architecture of the computer system will be described in the DMP.

10.3 Data handling

During data entry integrity checks help to minimize entry failures. These data entry checks are based on the data validation plan, signed by the LKP. The data entry system allows the trial monitors to control the entry process with the help of the built-in review functions. Comments and requests can be promptly processed by the trial site.

Checks for plausibility, consistency and completeness of the data will be performed during data entry. Based on these checks, queries will be produced. Any missing data or inconsistencies will be reported back to the respective site and clarified by the responsible investigator.

After completion of data entry and if no further corrections are to be made in the database, the access rights will be taken away and the database will be declared closed and used for statistical analysis.

All data management activities will be done according to the current Standard Operating Procedures (SOPs) of IZKS.

10.4 Storage and archiving of data

According to GCP, the investigator will archive all trial data (subject identification list, source data) and relevant correspondence in the Investigator Site File (ISF). The ISF, all source data and all documents itemized in section 8 of the ICH Consolidated Guideline on GCP will be archived after finalization of the trial according to the legal regulations.

Responsible for storage and archiving of the trial data (source data and CRFs) will be the principal investigator. Storage and archiving of the electronic data during the trial will be assured by IZKS. After completion of the trial all electronic data will be handed over to the Sponsor.

11 ETHICAL AND LEGAL ASPECTS

11.1 Good clinical practice

The procedures set out in this trial protocol, pertaining to the conduct, evaluation, and documentation of this trial, are designed to ensure that all persons involved in the trial abide by GCP and the ethical principles described in the current version of the Declaration of Helsinki. The trial will be carried out in keeping with local legal and regulatory requirements..

11.2 Subject information and *Informed Consent*

Before being admitted to the clinical trial, the subject must consent to participate after being fully informed about the nature, scope, and possible consequences of the clinical trial. Since the subjects in this trial are children, the consent of their parents or legally authorized representatives* must be sought.

The written information must be in a language understandable to the subject's parents or legally authorized representative and must specify who informed the subject's parents or legally authorized representative. The subject's parents or legally authorized representative should also have the opportunity to consult the Investigator, or a physician member of the investigational staff, about the details of the clinical trial.

A copy of the signed *Informed Consent* document must be given to the subject's parents or legally authorized representative. The original signed consent document will be retained by the investigator.

The investigator will not undertake any measures specifically required only for the clinical trial until valid consent has been obtained.

If the subject has a primary physician the investigator should inform the subject's primary physician about the subject's participation in the trial and if the subject's parents or legally authorized representative agree to the primary physician being informed.

After reading the *Informed Consent* document, the subject's parents or legally authorized representative must give consent in writing. The consent of the subject's parents or legally authorized representative must be confirmed by the personally dated signature of the subject's parents or legally authorized representative and by the personally dated signature of the person conducting the *Informed Consent* discussions.

An additional patient information and informed consent document for participation at the sub-study has to be signed by the subject's parents or legally authorized representative, if the subject takes part at the sub-study.

Footnote to define "legally authorized representative"

* "Legally authorized representative" means an individual or judicial or other body authorized under applicable law to consent on behalf of a prospective subject to the subject's participation in the procedure(s) involved in the research.

11.3 Confidentiality

The name of the subjects and other confidential information are subject to medical professional secrecy and the regulations of the local national laws on data protection. The name of the subjects and other confidential information will not be supplied to the Sponsor. During the clinical trial, subjects will be identified solely by means of an individual identification code (e.g. subject number, medication number). Trial findings stored on a computer will be stored in accordance with local data protection law and will be handled in strictest confidence. For protection of these data, organizational procedures are implemented to prevent distribution of data to unauthorized persons. The appropriate regulations of data legislation will be fulfilled in its entirety.

The subject will declare in the written consent to release the investigator from the medical professional secrecy to allow identification of subject's name and/or inspection of original data for monitoring purposes by health authorities and authorized persons (monitors).

The investigator will maintain a personal subject identification list (subject numbers with the corresponding subject names) to enable records to be identified.

11.4 Responsibilities of investigator

The investigator will ensure that the members of the investigational staff are adequately informed about the protocol, any amendments to the protocol, the trial treatments, and their trial-related duties and functions. Furthermore, he should nominate an adequately qualified deputy.

The investigator will maintain a list of the members of the investigational staff to whom he or she has delegated significant trial-related duties.
Any changes of the authorized trial personnel are to be communicated without delay to the Sponsor and the IZKS Mainz.

11.5 Approval of trial protocol and substantial amendments

Before the start of the trial, the trial protocol, *Informed Consent* document, and any other appropriate documents will be submitted to the independent ethics committee (IEC).

Approval (respectively formal approval) by the IEC should preferably mention the title of the trial, the trial code, if applicable the trial site, and the documents they reviewed. It must mention the date on which the decision was made and must be officially signed by a committee member.

This documentation must also include a list of members of the IEC present on the applicable EC meeting.

If applicable, the documents will also be submitted to the local national competent authorities (in Germany approval by the highest competent authority, BfArM), in accordance with the respective local legal requirements.

Investigational products can only be supplied to the investigator after documentation on all ethical and legal requirements for starting the clinical trial has been received by the Sponsor (IZKS Mainz: "regulatory green light"). Before the first subject is enrolled in the trial, all ethical and legal requirements must be met.

Neither the investigator nor the Sponsor will alter this trial protocol without obtaining the written agreement of the other. The IEC and, if applicable, the competent authorities must be informed of all subsequent protocol amendments and administrative changes, in accordance with the respective local legal requirements.

Amendments must be evaluated to determine whether formal approval must be sought and whether the *Informed Consent* document should also be revised.

The investigator must keep a record of all communications with the IEC and the competent authorities.

11.6 Continuous information to independent ethics committee

The EC must be informed by the Sponsor of all subsequent protocol amendments which require formal approval in accordance with the legal requirements.

The EC must be informed by the Sponsor of the end of the trial in accordance with legal requirements (within 90 days or within 15 days in case of premature closure of the clinical trial).

The independent EC must be informed of all subsequent protocol amendments and administrative changes, in accordance with local legal requirements.

11.7 Submission to local regulatory/competent authorities

Before the start of trial, the Sponsor is responsible for submission of all documents necessary to the local competent authorities for approval, in accordance to the respective local legal requirements. The local regulatory authority responsible for the investigator will be informed of the trial by the investigator.

The competent authorities must be informed by the Sponsor of all subsequent protocol amendments which require formal approval in accordance with the legal requirements.

The competent authorities must be informed by the Sponsor of the end of the trial in accordance with local national legal requirements.

The local regulatory authority responsible for the investigator must be informed by the investigator of the end of the trial in accordance with local national legal requirements.

11.8 Independent data safety and monitoring board (DSMB)

The independent data safety and monitoring board (DSMB) will supervise the conduct of this trial and will issue recommendations for early termination, modifications or continuation of the trial according to the DSMB Operating Procedure.

11.9 Insurance

According to current laws and regulations, the Sponsor has to subscribe to an insurance policy covering, in its terms and provisions, its legal liability for injuries caused to participating persons and arising out of this research performed strictly in accordance with the scientific protocol as well as with local national applicable laws and professional standards.

The insurance for Germany was taken out at Allianz Versicherungs-AG, Berlin (insurance number: AS-9100160962, maximum limit: € 500.000 per participating person, € 50.000.000 for all cases). For patients enrolled from first of January 2020, the insurance for Germany was taken out at Newline Europe Versicherung AG, Schanzenstraße 28a, 51063 Köln (insurance number: NEV 20957175A, maximum limit: € 500.000 per participating person, € 5.000.000 for all cases).

The insurance for Austria is Zürich Versicherungs AG, Schwarzenbergpaltz 15 ,A 1010 Vienne (insurance number 07229622-2). Otherwise identical conditions.

Any impairment of health which might occur in consequence of trial participation must be notified to the insurance company. The subject’s parents or legal representatives are responsible for notification.

The insured person's parents or legal representatives will agree to all appropriate measures serving for clarification of the cause and the extent of damage as well as the reduction of damage.

During the conduct of the trial, the subject must not undergo other clinical treatment except for cases of emergency. The subject's parents or legal representatives are bound to inform the investigator immediately about any adverse events and additionally drugs taken. The terms and conditions of the insurance should be delivered to the subject's parents or legal representatives.

Insurance provisions for this clinical trial are given in separate agreements.

11.10 Agreements

11.10.1 Financing of the trial

The trial is funded by the German Research Foundation (Project ID: ME 3827/1-1).

The general conditions of financing for this trial are given in separate agreements.

11.10.2 Report

After conclusion of the trial, a report shall be written by the Sponsor, in cooperation with the coordinating investigator. The report will include a statistical analysis and an appraisal of the results from a medical viewpoint. It will be based on the items listed in this trial protocol.

Pursuant to §42b AMG the investigators assent to the disclosure of their names in the report.

11.10.3 Publication policy

Any publication of the results, either in part or in total (articles in journals or newspapers, oral presentation, etc.) by the investigators, their representatives, or by the Sponsor, shall require the approval of Prof. Dr. med. L. Gortner and Prof. Dr. med. S. Meyer. It is planned to publish the results of the trial as an original article in an appropriate medical journal as well as presentation at congresses. Prof. Dr. med. S. Meyer is first author of the article and will present the data at the major congresses. The choice of the journal for the publication will be made by Prof. Dr. med. L. Gortner and Prof. Dr. med. S. Meyer in agreement with the co-authors. Besides the Principal Investigator, further authors of this article have to meet at least one of the following points:

- Substantial contribution to the recruitment of subjects
- Substantial contribution to interpretation of the data
- Substantial contribution to drafting the trial protocol
- Substantial contribution to drafting the article or revising it critically for important intellectual content

12 SIGNATURES

The present trial protocol was subject to critical review and has been approved in the present version by the persons undersigned. The information contained is consistent with:

- The current risk-benefit assessment of the investigational medicinal product.
- The moral, ethical, and scientific principles governing clinical research as set out in the Declaration of Helsinki and the principles of GCP.

Sponsor/ Operating Institution of Sponsor

Name: Prof. Dr. med. Sascha Meyer

_________________ _________________________________

Date Signature

Coordinating Investigator

Name: Prof. Dr. med. Sascha Meyer

_________________ _________________________________

Date Signature

Biometrician

Name: Christian Ruckes, Dipl. Math.

_________________ _________________________________

Date Signature

13 DECLARATION OF INVESTIGATOR

I have read the above trial protocol and I confirm that it contains all information to accordingly conduct the clinical trial. I pledge to conduct the clinical trial according to the protocol.

I will enroll the first subject only after all ethical and regulatory requirements are fulfilled. I pledge to obtain written consent for trial participation from all subjects' parents or legally authorized representatives.

I know the requirements for accurate notification of serious adverse events and I pledge to document and notify such events as described in the protocol.

I pledge to retain all trial-related documents and source data as described. I will provide a Curriculum Vitae (CV) before trial start. I agree that the CV may be submitted to the responsible competent authorities.

I will conduct the trial in compliance with the protocol, GCP and the applicable regulatory requirements.

Investigator

Name
Address
Phone
Fax
E-mail

Date Name Signature

Deputy Investigator

Name
Address
Phone
Fax
E-mail

Date Name Signature

14 REFERENCES

1. Smith, V.C., et al., *Trends in severe bronchopulmonary dysplasia rates between 1994 and 2002.* J Pediatr, 2005. **146**(4): p. 469-73.

2. Thomas, W. and C.P. Speer, *Management of infants with bronchopulmonary dysplasia in Germany.* Early Hum Dev, 2005. **81**(2): p. 155-63.

3. Jobe, A.H. and E. Bancalari, *Bronchopulmonary dysplasia.* Am J Respir Crit Care Med, 2001. **163**(7): p. 1723-9.

4. Schmidt, B., et al., *Impact of bronchopulmonary dysplasia, brain injury, and severe retinopathy on the outcome of extremely low-birth-weight infants at 18 months: results from the trial of indomethacin prophylaxis in preterms.* JAMA, 2003. **289**(9): p. 1124-9.

5. Mercier, J.C., et al., *Inhaled nitric oxide for prevention of bronchopulmonary dysplasia in premature babies (EUNO): a randomised controlled trial.* Lancet, 2010. **376**(9738): p. 346-54.

6. Van Marter, L.J., et al., *Does bronchopulmonary dysplasia contribute to the occurrence of cerebral palsy among infants born before 28 weeks of gestation?* Arch Dis Child Fetal Neonatal Ed, 2010. **96**(1): p. F20-9.

7. Humphrey, J.H., et al., *Impact of neonatal vitamin A supplementation on infant morbidity and mortality.* J Pediatr, 1996. **128**(4): p. 489-96.

8. Rahmathullah, L., et al., *Impact of supplementing newborn infants with vitamin A on early infant mortality: community based randomised trial in southern India.* BMJ, 2003. **327**(7409): p. 254.

9. World Bank, *The world bank development report 1993: investing in health*. 1993, Oxford University Press: Oxford.

10. Humphrey, J.H. and A.L. Rice, *Vitamin A supplementation of young infants.* Lancet, 2000. **356**(9227): p. 422-4.

11. Mactier, H. and L.T. Weaver, *Vitamin A and preterm infants: what we know, what we don't know, and what we need to know.* Arch Dis Child Fetal Neonatal Ed, 2005. **90**(2): p. F103-8.

12. Inder, T.E., et al., *Plasma vitamin A levels in the very low birthweight infant--relationship to respiratory outcome.* Early Hum Dev, 1998. **52**(2): p. 155-68.

13. Peeples, J.M., et al., *Vitamin A status of preterm infants during infancy.* Am J Clin Nutr, 1991. **53**(6): p. 1455-9.

14. Mactier, H., et al., *Vitamin A provision for preterm infants: are we meeting current guidelines?* Arch Dis Child Fetal Neonatal Ed, 2011. **96**(4): p. F286-9.

15. Shenai, J.P., *Vitamin A*, in *Nutritional needs of the preterm infant: scientific basis and practical guidelines*, R.C. Tsang, et al., Editors. 1993, Williams and Williams: Baltimore. p. 87-100.

16. Ong, D.E., *A novel retinol-binding protein from rat. Purification and partial characterization.* J Biol Chem, 1984. **259**(3): p. 1476-82.

17. Biesalski, H.K. and D. Nohr, *Importance of vitamin-A for lung function and development.* Mol Aspects Med, 2003. **24**(6): p. 431-40.

18. Metzler M. D., S.J.M., *Retinoic acid differentially regulates expression of surfactant-associated proteins in human fetal lung.* Endocrinology, 1993. **133**(5): p. 1990-8.

19. Shenai, J.P., et al., *Plasma retinol-binding protein response to vitamin A administration in infants susceptible to bronchopulmonary dysplasia.* J Pediatr, 1990. **116**(4): p. 607-14.

20. Northway, W.H., Jr., R.C. Rosan, and D.Y. Porter, *Pulmonary disease following respirator therapy of hyaline-membrane disease. Bronchopulmonary dysplasia.* N Engl J Med, 1967. **276**(7): p. 357-68.

21. Brandt, R.B., et al., *Serum vitamin A in premature and term neonates.* J Pediatr, 1978. **92**(1): p. 101-4.

22. Shenai, J.P., et al., *Plasma vitamin A and retinol-binding protein in premature and term neonates.* J Pediatr, 1981. **99**(2): p. 302-5.

23. Kennedy, K.A., *Epidemiology of acute and chronic lung injury.* Semin Perinatol, 1993. **17**(4): p. 247-52.

24. Darlow, B.A. and P.J. Graham, *Vitamin A supplementation for preventing morbidity and mortality in very low birthweight infants.* Cochrane Database Syst Rev, 2002(4): p. CD000501.

25. Shenai, J.P., et al., *Clinical trial of vitamin A supplementation in infants susceptible to bronchopulmonary dysplasia.* J Pediatr, 1987. **111**(2): p. 269-77.

26. Tyson, J.E., et al., *Vitamin A supplementation for extremely-low-birth-weight infants. National Institute of Child Health and Human Development Neonatal Research Network.* N Engl J Med, 1999. **340**(25): p. 1962-8.

27. Mactier, H., et al., *Vitamin A supplementation improves retinal function in infants at risk of retinopathy of prematurity.* J Pediatr, 2012. **160**(6): p. 954-9 e1.

28. Hack, M., et al., *Neurodevelopment and predictors of outcomes of children with birth weights of less than 1000 g: 1992-1995.* Arch Pediatr Adolesc Med, 2000. **154**(7): p. 725-31.

29. Wagner, E., T. Luo, and U.C. Drager, *Retinoic acid synthesis in the postnatal mouse brain marks distinct developmental stages and functional systems.* Cereb Cortex, 2002. **12**(12): p. 1244-53.

30. Zile, M.H., *Function of vitamin A in vertebrate embryonic development.* J Nutr, 2001. **131**(3): p. 705-8.

31. Stephensen, C.B., et al., *Vitamin A enhances in vitro Th2 development via retinoid X receptor pathway.* J Immunol, 2002. **168**(9): p. 4495-503.

32. Stephensen, C.B., *Vitamin A, infection, and immune function.* Annu Rev Nutr, 2001. **21**: p. 167-92.

33. Baraldi, E. and M. Filippone, *Chronic lung disease after premature birth.* N Engl J Med, 2007. **357**(19): p. 1946-55.

34. Gortner, L., et al., *Rates of bronchopulmonary dysplasia in very preterm neonates in Europe: results from the MOSAIC cohort.* Neonatology, 2010. **99**(2): p. 112-7.

35. The Committee for Medicinal Products for Human Use (CHMP) and paediatric committee (PDCO). *Guideline on the investigation of medicinal products in the term and preterm neonate*. 2010 [cited 2014, January 20th]; Available from: http://www.ema.europa.eu/docs/en_GB/document_library/Scientific_guideline/2009/09/WC500003754.pdf.

36. Darlow, B.A. and P.J. Graham, *Vitamin A supplementation to prevent mortality and short and long-term morbidity in very low birthweight infants.* Cochrane Database Syst Rev, 2007(4): p. CD000501.

37. Wardle, S.P., et al., *Randomised controlled trial of oral vitamin A supplementation in preterm infants to prevent chronic lung disease.* Arch Dis Child Fetal Neonatal Ed, 2001. **84**(1): p. F9-F13.

38. Bassler, D., et al., *The Neonatal European Study of Inhaled Steroids (NEUROSIS): an eu-funded international randomised controlled trial in preterm infants.* Neonatology, 2010. **97**(1): p. 52-5.

39. BMG (Bundesministerium für Gesundheit). *Bekanntmachung eines Beschlusses des Gemeinsamen Bundesausschusses über eine Änderung der "Vereinbarung über Maßnahmen zur Qualitätssicherung der Versorgung von Früh- und Neugeborenen",*. eBAnz AT11.11.2013 B3 2013 [cited 2014, January 20th]; Available from: https://www.bundesanzeiger.de/ebanzwww/wexsservlet?session.sessionid=464268b8b5f76b562324387cf755274b&page.navid=detailsearchlisttodetailsearchdetail&fts_search_list.selected=b4bec0e8dc776f6c&fts_search_list.destHistoryId=72027.

40. Griffiths R., *The Abilities of Babies: a Study in Mental Measurement.* 1976, Bucks: Amersham, UK.

41. Bayley N., *Bayley Scales of Infant Development*. Second Edition ed. 1993, San Antonio, TX: The Psychological Cooperation.

42. Massaro, D. and G.D. Massaro, *Lung development, lung function, and retinoids.* N Engl J Med, 2010. **362**(19): p. 1829-31.

43. Checkley, W., et al., *Maternal vitamin A supplementation and lung function in offspring.* N Engl J Med, 2010. **362**(19): p. 1784-94.

44. Koletzko, B., et al., *1. Guidelines on Paediatric Parenteral Nutrition of the European Society of Paediatric Gastroenterology, Hepatology and Nutrition (ESPGHAN) and the European Society for Clinical Nutrition and Metabolism (ESPEN), Supported by the European Society of Paediatric Research (ESPR).* J Pediatr Gastroenterol Nutr, 2005. **41 Suppl 2**: p. S1-87.

45. Greene, H.L., et al., *Guidelines for the use of vitamins, trace elements, calcium, magnesium, and phosphorus in infants and children receiving total parenteral nutrition: report of the Subcommittee on Pediatric Parenteral Nutrient Requirements from the Committee on Clinical Practice Issues of the American Society for Clinical Nutrition.* Am J Clin Nutr, 1988. **48**(5): p. 1324-42.

46. Agostoni, C., et al., *Enteral nutrient supply for preterm infants: commentary from the European Society of Paediatric Gastroenterology, Hepatology and Nutrition Committee on Nutrition.* J Pediatr Gastroenterol Nutr, 2009. **50**(1): p. 85-91.

47. Kaplan, H.C., et al., *Understanding variation in vitamin A supplementation among NICUs.* Pediatrics, 2010. **126**(2): p. e367-73.

48. Zachman, R.D., et al., *Use of the intramuscular relative-dose-response test to predict bronchopulmonary dysplasia in premature infants.* Am J Clin Nutr, 1996. **63**(1): p. 123-9.

49. Georgieff, M.K., et al., *Effect of postnatal steroid administration on serum vitamin A concentrations in newborn infants with respiratory compromise.* J Pediatr, 1989. **114**(2): p. 301-4.

50. Shenai, J.P., B.G. Mellen, and F. Chytil, *Vitamin A status and postnatal dexamethasone treatment in bronchopulmonary dysplasia.* Pediatrics, 2000. **106**(3): p. 547-53.

51. Georgieff, M.K., et al., *The effect of glucocorticosteroids on serum, liver, and lung vitamin A and retinyl ester concentrations.* J Pediatr Gastroenterol Nutr, 1991. **13**(4): p. 376-82.

52. Biesalski H.K., *The importance of vitamin A for the development and function of lungs in newborns.* Sight and Life, 2011. **25**: p. 16-29.

53. Koletzko, B., et al., *1. Guidelines on Paediatric Parenteral Nutrition of the European Society of Paediatric Gastroenterology, Hepatology and Nutrition (ESPGHAN) and the European Society for Clinical Nutrition and Metabolism (ESPEN), Supported by the European Society of Paediatric Research (ESPR).* J Pediatr Gastroenterol Nutr, 2005. **41 Suppl 2**: p. S1-87.

54. Ambalavanan, N., et al., *A comparison of three vitamin A dosing regimens in extremely-low-birth-weight infants.* J Pediatr, 2003. **142**(6): p. 656-61.

55. Woodruff, C.W., et al., *Vitamin A status of preterm infants: the influence of feeding and vitamin supplements.* Am J Clin Nutr, 1986. **44**(3): p. 384-9.

56. Schmiedchen, B., et al., *The relative dose response test based on retinol-binding protein 4 is not suitable to assess vitamin a status in very low birth weight infants.* Neonatology, 2014. **105**(2): p. 155-60.

57. Rush, M.G., et al., *Intramuscular versus enteral vitamin A supplementation in very low birth weight neonates.* J Pediatr, 1994. **125**(3): p. 458-62.

58. Landman, J., et al., *Comparison of enteral and intramuscular vitamin A supplementation in preterm infants.* Early Hum Dev, 1992. **30**(2): p. 163-70.

59. Bental, R.C., PA; Cummins, RR; Sandler, DL; Wainer, S; Rotschild A, *Vitamin A therapy - effects on the incidence of bronchopulmonary dysplasia.* South African Journal of Food Science and Nutrition, 1994. **6**(4): p. 141-5.

60. Papagaroufalis, C., et al., *A trial of vitamin A supplementation in infants susceptible to bronchopulmonary dysplasia [abstract].* Pediatric Research, 1988. **23**: p. 518A.

61. Pearson, E., et al., *Trial of vitamin A supplementation in very low birth weight infants at risk for bronchopulmonary dysplasia.* J Pediatr, 1992. **121**(3): p. 420-7.

62. Ravishankar, C., et al., *A trial of vitamin A therapy to facilitate ductal closure in premature infants.* J Pediatr, 2003. **143**(5): p. 644-8.

63. European Commission. *Ethical considerations for clinical trials on medicinal products conducted with the paediatric population*. 2008 [cited 2014, January 20th]; Available from: http://ec.europa.eu/health/files/eudralex/vol-10/ethical_considerations_en.pdf.

64. Short, E.J., et al., *Developmental sequelae in preterm infants having a diagnosis of bronchopulmonary dysplasia: analysis using a severity-based classification system.* Arch Pediatr Adolesc Med, 2007. **161**(11): p. 1082-7.

65. Short, E.J., et al., *Cognitive and academic consequences of bronchopulmonary dysplasia and very low birth weight: 8-year-old outcomes.* Pediatrics, 2003. **112**(5): p. e359.

66. Spears, K., C. Cheney, and J. Zerzan, *Low plasma retinol concentrations increase the risk of developing bronchopulmonary dysplasia and long-term respiratory disability in very-low-birth-weight infants.* Am J Clin Nutr, 2004. **80**(6): p. 1589-94.

67. Speer, C.P. and M. Silverman, *Issues relating to children born prematurely.* Eur Respir J Suppl, 1998. **27**: p. 13s-16s.

68. Smith, V.C., et al., *Rehospitalization in the first year of life among infants with bronchopulmonary dysplasia.* J Pediatr, 2004. **144**(6): p. 799-803.

69. Hennessy, E.M., et al., *Respiratory health in pre-school and school age children following extremely preterm birth.* Arch Dis Child, 2008. **93**(12): p. 1037-43.

70. Doyle, L.W., et al., *Bronchopulmonary dysplasia in very low birth weight subjects and lung function in late adolescence.* Pediatrics, 2006. **118**(1): p. 108-13.

71. Wong, P.M., et al., *Emphysema in young adult survivors of moderate-to-severe bronchopulmonary dysplasia.* Eur Respir J, 2008. **32**(2): p. 321-8.

72. Dinakar, C., *Anaphylaxis in children: current understanding and key issues in diagnosis and treatment.* Curr Allergy Asthma Rep, 2012. **12**(6): p. 641-9.

73. Arbeitsgemeinschaft der Wissenschaftlichen Medizinischen Fachgesellschaften e.V. (AWMF). *S2k Leitlinie: Nekrotisierende Enterokolitis (NEK)*. 1996 11/2010 [cited 2014, January 20th]; Available from: http://www.awmf.org/leitlinien/detail/ll/024-009.html.

74. Walsh, M.C., et al., *Impact of a physiologic definition on bronchopulmonary dysplasia rates.* Pediatrics, 2004. **114**(5): p. 1305-11.

75. Yoder, B.A., et al., *Heated, humidified high-flow nasal cannula versus nasal CPAP for respiratory support in neonates.* Pediatrics, 2013. **131**(5): p. e1482-90.

76. Craft, N.E., et al., *Retinol analysis in dried blood spots by HPLC.* J Nutr, 2000. **130**(4): p. 882-5.

77. Erhardt, J.G., et al., *Rapid and simple measurement of retinol in human dried whole blood spots.* J Nutr, 2002. **132**(2): p. 318-21.

78. Erhardt, J.G., et al., *Combined measurement of ferritin, soluble transferrin receptor, retinol binding protein, and C-reactive protein by an inexpensive, sensitive, and simple sandwich enzyme-linked immunosorbent assay technique.* J Nutr, 2004. **134**(11): p. 3127-32.

79. International Committee for the Classification of Retinopathy of Prematurity, *The International Classification of Retinopathy of Prematurity revisited.* Arch Ophthalmol, 2005. **123**(7): p. 991-9.

80. Arbeitsgemeinschaft der Wissenschaftlichen Medizinischen Fachgesellschaften e.V. (AWMF). *S1 Leitlinie: Augenärztliche Screening-Untersuchung von Frühgeborenen*. 1998 update currently in progress [cited 2014, January 20th]; Available from: http://www.awmf.org/leitlinien/detail/ll/024-010.html.

81. Riccabona M., et al. *Standarddokumentation der Sonografie des kindlichen Neurokraniums*. Dokumentationsleitlinien [cited 2014, January 20th]; Available from: http://www.degum.de/fileadmin/dokumente/sektionen/paediatrie/dokumentationsempfehlungen/P__08_Poster_SCh_del_Dokumentationsempfehlung_Sch_del__Poster_.pdf.

82. Papile, L.A., et al., *Incidence and evolution of subependymal and intraventricular hemorrhage: a study of infants with birth weights less than 1,500 gm.* J Pediatr, 1978. **92**(4): p. 529-34.

83. Hill, A., et al., *Hemorrhagic periventricular leukomalacia: diagnosis by real time ultrasound and correlation with autopsy findings.* Pediatrics, 1982. **69**(3): p. 282-4.

84. Bell, R.S., *Neonatal necrotizing enterocolitis.* N Engl J Med, 1970. **283**(3): p. 153-4.

85. Walsh, M.C., R.M. Kliegman, and M. Hack, *Severity of necrotizing enterocolitis: influence on outcome at 2 years of age.* Pediatrics, 1989. **84**(5): p. 808-14.
