# Supplementary material for: Two-year outcomes after early postnatal high-dose fat-soluble enteral vitamin A supplementation in extremely low birth weight infants: follow-up of the NeoVitaA randomized controlled trial
Source: eClinicalMedicine. 2025 Sep 15;89:103495. doi: 10.1016/j.eclinm.2025.103495 (PMC12675030; doi:10.1016/j.eclinm.2025.103495)
Supplement: Statistical Analysis Plan [file mmc4.pdf]

IZKS
Interdisziplinäres Zentrum
Klinische Studien Mainz

A prospective, multicenter, double blind, placebo-controlled, two-arm parallel group phase 3 trial to evaluate the effect of early postnatal additional high dose oral vitamin A supplementation of 5000 IU/kg/d versus placebo for 28 days for preventing bronchopulmonary dysplasia (BPD) or death in extremely low birth weight (ELBW) infants.

NeoVitaA Trial

Statistical Analysis Plan

Version 1.0 05.04.2023

EudraCT Number: 2013-001998-24
Clinical Trial Code (IZKS): 2012-008 NeoVitaA

Prof. Dr. Sascha Meyer<br>Coordinating Investigator	[illegible]<br>Date, Signature
Dr. Christian Ruckes<br>Statistician	5.4.2023 Christian Ruckes<br>Date, Signature

1 Background

Bronchopulmonary dysplasia (BPD) affects as many as 35% of extremely low birth weight infants (ELBW; birth weight < 1000 g) [1, 2]. BPD is defined as the need for oxygen supplementation at 36 weeks postmenstrual age or at date of discharge to home, whichever comes first (PMA) [3]. The disease is marked by respiratory compromise and is associated with high mortality and severe long-term morbidity [4], including cerebral palsy. Unfortunately, many therapeutic approaches are non-effective (e.g., inhaled nitric oxide) or have significant side effects (administration of steroids). The beneficial role of intramuscular (i.m.) vitamin A (VA) supplementation has been demonstrated in a recent Cochrane meta-analysis; however, because of the substantial pain associated with repetitive i.m. injection, this form of VA supplementation is not common practice. Moreover, recent data indicate that there is substantial variation in how and how much VA is given to preterm infants in different Neonatal Intensive Care Units (NICUs).

The aim of this trial is to assess the role of early postnatal additional high-dose oral VA supplementation versus placebo for 28 days for preventing BPD or death in ELBW infants.

Bronchopulmonary dysplasia is associated with an increased mortality rate and continues to carry significant long-term morbidity. Although a variety of medical interventions have been examined, most of them have proven to be futile (e.g., inhaled nitric oxide), or the interventions themselves carry substantial side effects (e.g., early administration of steroids). The intra-muscular administration of VA has been shown to be effective in significantly reducing the rate of BPD in preterm infants. However, given the fact that i.m. VA injections are painful, and that they have to be repeated several times, this practice has not been widely accepted in neonatal intensive care medicine.

Therefore, a randomized controlled trial (RCT) that will assess the role of early postnatal additional high-dose oral VA supplementation to reduce and prevent the occurrence of BPD or death in this susceptible cohort (ELBW infants) is warranted.

This statistical analysis plan (SAP) covers the analyses from the final analysis. The analyses for the interim analysis were described in a separate document.

1.1 Study Objectives

Primary:
To investigate whether early postnatal additional high dose oral vitamin A supplementation (5000 IU/kg body weight/day) for 28 days reduces the absolute risk of bronchopulmonary dysplasia (moderate/severe) or death at 36+0 weeks PMA or at date of discharge to home, whichever comes first in ELBW infants when compared to placebo treatment.

Secondary:
To assess the impact on high dose oral vitamin A supplementation on:

1. All-cause mortality
2. All grade BPD (mild/moderate/severe)
3. Duration of Positive pressure ventilation (PPV) and Positive pressure support (PPS)
4. Duration of evolving BPD since birth
5. Serum VA status
6. Retinopathy of prematurity (ROP)
7. Intraventricular hemorrhage (IVH)
8. Periventricular leukomalacia (PVL)
9. Necrotizing enterocolitis (NEC)
10. Safety and tolerability of trial medication

11. Pulmonary assessment at 12 and 24 months c. a.
12. Neurological assessment at 24 months c.a.
13. Anthropoemetric data

1.2 Study Design

A prospective, multicenter, double-blind, randomized, placebo-controlled parallel group trial

2 Analysis Populations

2.1 Definitions

The analysis will be performed for the Intention-to-treat (ITT), the per protocol (PP) population and the safety population.

All randomized subjects will be included in the Intention-to-treat (ITT) population. For the ITT population subjects will be assigned to the treatment to which they were randomized.

The PP population consists of all patients of the ITT population without any major protocol violation.

- Treatment as randomized
- Treatment compliance of at least 75% measured by drug accountability
- No visits attended out of schedule
- Meeting all inclusion criteria and not meeting any exclusion criteria
- Unavailability/uncertainty of the primary endpoint parameter

The safety population comprises all subjects who received at least one dose of trial treatment. For the safety population subjects will be assigned to the treatment to which they actually received.

2.2 Scope

After the interim analysis it was decided to continue the study after the interim analysis. The two-sided significance level was 0.0052 for the interim analysis and 0.0480 for the final analysis. This document applies to the final analysis only.

2.3 Major Protocol Violations

The following derivations from the protocol will be considered as major protocol violations (see also section 2.1):

- Treatment compliance of less than 70% or more than 130% measured by drug accountability
- Violation of at least one inclusion or exclusion criterion
- Unavailability/uncertainty of the primary endpoint parameter
- Other major protocol violations documented in the CRF (case by case decision, if relevant)
- Treatment given not as randomized

Details and individual decisions are documented in 2023_02_23_Blinded Data Meeting NeoVitaA_final.pptx and its appendix.

3 Study Centres

28 trial sites in Germany (26) and Austria (2). For analysis trial sites with less than two events in at least one treatment group will be aggregated.

4 Analysis Variables

The following parameters were collected in this study. Only the parameters in bold letters were considered for analysis.

4.1 Patient disposition

- **Number of patients fulfilling inclusion criteria**
- **Number of patients negating exclusion criteria**
- **Number of patients in the ITT and safety population**
- **Number of patients completing the trial**
- **Reasons for premature end of study**
- **Number of patients (from randomization) in the study**
  - **Day 1**
  - **Days 2-7**
  - **Days 8-14**
  - **Days 15-21**
  - **Days 22-28**

4.2 Maternal Demographics and obstetric history

- **Maternal Age [years]**
- **Maternal Weight [kg]**
- **Maternal Height [cm]**
- **Gravida including current pregnancy**
- **Parity including current pregnancy**
- **Current pregnancy: singleton or multiple? (singleton/multiple)**
- **Premature rupture of membranes (yes/no)**
- **Prolonged rupture of membranes (yes/no)**
- **Pregnancy induced hypertension (yes/no)**
- **Maternal pre-eclamptic toxemia (yes/no)**
- **Maternal smoking during pregnancy (yes/no)**
- **Maternal recreational drug use (yes/no)**
- **Other complications during pregnancy (yes/no)**

4.3 Neonatal birth history

- **Mode of delivery (Assisted vaginal delivery without instruments/ Assisted vaginal delivery with instruments/Non-emergency cesarean section/emergency/cesarean section)**
- **Umbilical arterial pH**
- **APCAR score 1 min, 5 min, 10 min**
- **Birth complications (yes/no)**
- **Kind of birth complications (Asphyxia/Hemorrhage/Other)**

4.4 Neonatal demographics

- **Gestational age [days]**
- **Postmenstrual age [days]**
- **Gender (male/female)**

- **Race (Caucasian/African/Asian/Mixed parentage/Other)**

4.5 Assessment of neonatal growth

- **Birth weight [g]**
- **Birth length [cm]**
- **Birth head circumference [cm]**

4.6 Extent of exposure (derived)

- **Number of days in the study (from randomization to regular end of study or drop-out)**
- **Number of IE units administered (from randomization to regular end of study or drop-out). The number of IE units will be derived from the number of drops.**
- **Average number of IE units administered per day (from randomization to regular end of study or drop-out)**
- **Kind of enteral nutrition (parental nutrition without minimal enteral feeds, parental nutrition with minimal enteral feeds, parental and enteral nutrition, full enteral nutrition, no nutrition without minimal enteral feeds, no nutrition with minimal enteral feeds)**
- **Type of enteral nutrition (formula, breast milk, formula + breast milk, formula + fortifier, breast milk + fortifier, formula + breast milk + fortifier)**
- **Basic therapy of VA supplementation: Number of days and total dose**

4.7 Primary Variable

The primary variable is the incidence of "BPD (moderate/severe) or death" (yes/no). Timepoint: at 36+0 weeks PMA or at date of discharge to home, whichever comes first. If end of treatment is not reached or the date is not available, the patient is considered as censored at the last available end of treatment date (end of trial visit date or date of study end).

The primary variable will be compared between treatment groups by a logistic regression analysis with treatment group and trial site as covariates at a global two-sided significance level of 5% (0.52% for interim analysis and 4.8% for final analysis). Trial sites with less than two events in at least one treatment group will be aggregated in order to increase robustness of estimation results. A discrepancy between stratification factors at randomization and covariates in the analysis is thus accepted.

4.8 Secondary Variables

4.8.1 Efficacy

The following parameters were collected in this study. Only the parameters in bold letters were considered for the interim analysis.

1. **All-cause mortality**
2. **All grade BPD (mild/moderate/severe)**
3. **Duration of PPV and PPS**
4. **Duration of evolving BPD since birth**
5. **Serum VA status (Serum retinol levels, RBP, RE)**
6. **Retinopathy of prematurity (ROP)**
7. **Intraventricular hemorrhage (IVH)**
8. **Periventricular leukomalacia (PVL)**
9. **Necrotizing enterocolitis (NEC)**

10. Occurrence of any adverse event
11. Total number of antibiotic treatments at 12 and 24 months of c.a.
12. Total number of antibiotic treatments for pulmonary infections at 12 and 24 months of c.a.
13. Total number of hospital admissions at 12 and 24 months of c.a.
14. Total number of hospital admissions for pulmonary infections at 12 and 24 months of c.a.
10. *Safety and tolerability of trial medication (not an efficacy variable, see safety analyses)*
11. Pulmonary assessment at 12 and 24 months c.a. (antibiotic treatments (yes/no, number, number because of pulmonary infections), hospital admissions (yes/no, number, number because of pulmonary infections))
12. Neurological assessment at 24 months c.a. (Results of Bayley III scale: Scale Language (sign, value), MDI ("Mental Development Index", sign, value), PDI ("Psychomotor Development Index") sign, value). Neurological diseases and their drugs and physiotherapy, Non-neurological diseases and their drug and therapy.
13. Anthropoemetric data (weight, height, head circumference)

4.8.2 Safety

4.8.2.1 Adverse Events
All summaries and listings of safety data will be performed for the safety population.
AEs will be coded according to MedDRA terminology. Detailed information collected for each AE will include: A description of the event, duration, whether the AE was serious, intensity, relationship to trial drug, action taken, clinical outcome.

4.8.2.2 Laboratory values
Liver function tests: ALT, AST, CHE, Bilirubin, ALP
Renal function tests: Urea, Creatinine, Sodium, Potassium, Calcium
Hematological parameters: Hb, WBC, differential count including reticulocytes, platelets

4.8.2.3 Cranial Ultrasound Investigations
Screening cranial ultrasound investigations will be performed at the following time points:
- Once between days of life 1-3
- On day 7 of life (± 1 day)
- On treatment day 28, (± 1 day) and
- On day 36+0 weeks PMA or at date of discharge to home, whichever comes first (± 1 day)

If bulging fontanelle was found on assessment of clinical condition, an additional CUSS examination including PW-duplex/Doppler sonography should be performed and results documented in the eCRF. Pathological PW-duplex/Doppler sonography, which is not explained by IVH or by a persistent ductus arteriosus, should prompt a further, detailed clinical assessment and must be reported as concomitant disease or AE.

4.8.3 Quality of Life

Not applicable.

4.8.4 Health Economics

Not applicable.

5 Treatment of Missing Values and Outliers

5.1 Missing Values

Missing values will not be replaced.

5.2 Outliers

No methods to detect outliers will be applied.

6 Statistical Analyses

Categorical data will be analysed descriptively by absolute and relative frequencies. For continuous or quasi-continuous data the descriptive statistics: N (number of non-missing values), mean, standard deviation, minimum, median, and maximum will be presented.

6.1 Patient Disposition

Patient disposition will be presented for all patients included in the study.

6.2 Maternal Demographics and obstetric history

The maternal demographics and obstetric history (see section 4.1) will be presented by descriptive statistics.

6.3 Neonatal birth history

All neonatal birth history data (see section 4.2) will be presented by descriptive statistics.

6.4 Neonatal demographics

All neonatal demographics data (see section 4.3) will be presented by descriptive statistics.

6.5 Assessment of neonatal growth

All neonatal growth data (see section 4.4) will be presented by descriptive statistics.

6.6 Previous Medication and Concomitant Diseases

Summary tables will present the number of subjects observed with neonatal prior and concomitant diseases by MedDRA System Organ Class and Preferred Term and corresponding percentages.
Absolute and relative frequencies for the CRF listed substances and the indication will be displayed.
Summary tables will present the number of maternal prior and concomitant diseases by MedDRA System Organ Class and Preferred Term and corresponding percentages.

6.7 Extent of Exposure

Sample characteristics for the number of days in the study, number of drops administered, and average number of drops administered per day will be displayed. Kind and type of enteral nutrition will be displayed by absolute and relative frequencies. For the basic therapy of VA supplementation sample characteristics of number of days and total dose will be provided.

6.8 Compliance

Not applicable.

6.9 Primary Analysis

Description of the primary efficacy analysis and population:

Incidence of BPD (moderate/severe) or death at 36+0 weeks PMA or at date of discharge to home, whichever comes first will be compared between treatment groups by a logistic

regression analysis with treatment group as fixed effect and trial site as covariate at a global two-sided significance level of 5% (0.52% for interim analysis and 4.8% for final analysis). For the final analysis the odds ratio together with the 95% Confidence Interval and with the 95.2% Confidence Interval will be presented.
Trial sites with less than two events in at least one treatment group will be aggregated in order to increase robustness of estimation results. A discrepancy between stratification factors at randomization and covariates in the analysis is thus accepted.

The following null hypothesis will be tested:

H_0: $\pi_A = \pi_P$ versus H_1: $\pi_A \neq \pi_P$

Where π_A, π_P is the true moderate/severe BPD rate in the Vitamin A treatment group and the placebo treatment group, respectively.

Sample code:

```
PROC LOGISTIC DATA=data1;
      CLASS group;
      MODEL primvar = group centre / alpha=0.048;
      QUIT;
```

For sensitivity the differences of the primary endpoint between the two treatment groups will be analysed by a Chi Square test. The primary analysis will be performed for the ITT and PP population. The odds ratio together with the 95% Confidence Interval and with the 95.2% Confidence Interval will be presented.

Sample code:

```
PROC FREQ DATA=data1;
      TABLES primvar*group / chisq relrisk alpha=0.048;
      QUIT;
```

Additionally, social parameters and use of physical therapies will be compared by Chi-square tests and their influence on the development of BPD will be investigated by means of a logistic regression model with social parameters and physical therapies included in the model.

6.10 Secondary Analyses

6.10.1 Efficacy

Secondary endpoints:

1. All-cause mortality: The number of deaths will be compared by a Chi Square test.
2. All grade BPD (mild/moderate/severe): If a BPD occurred and the grade by neonate will be determined and tabulated. BPD occurred (yes or no) will be compared by a Chi Square test between groups.
3. Duration of PPV and PPS: The duration of respiratory ventilation/support by neonate will be determined and tabulated. If 'both' is documented in the database, the respectively ventilation time will be split equally to ventilation and support. The duration of respiratory ventilation/support will be compared by a Wilcoxon test between groups.
4. Duration of evolving BPD since birth: The duration of BPD will be determined (BPD-day=yes, last date minus first date + 1) and compared by a Wilcoxon test between groups.
5. Serum VA status: Sample characteristics for Serum retinol levels, RBP, RE will be displayed for the vitamine A treatment group.

6. Retinopathy of prematurity (ROP): If a ROP occurred and the (maximum) grade by neonate will be determined and tabulated. ROP occurred (yes or no) will be compared by a Chi Square test between groups. This analysis will be performed for the ITT and PP population.
7. Intraventricular hemorrhage (IVH): If a IVH occurred and the (maximum) grade by neonate will be determined and tabulated. IVH occurred (yes or no) will be compared by a Chi Square test between groups. This analysis will be performed for the ITT and PP population.
8. Periventricular leukomalacia (PVL): If a PVL occurred and the (maximum) intensity by neonate will be determined and tabulated. PVL occurred (yes or no) will be compared by a Chi Square test between groups. This analysis will be performed for the ITT and PP population.
9. Necrotizing enterocolitis (NEC): If a NEC occurred will be determined and tabulated. NEC occurred (yes or no) will be compared by a Chi Square test between groups. This analysis will be performed for the ITT and PP population.
10. Safety and tolerability of trial medication (see section 6.8.2)
11. Pulmonary assessment at 12 and 24 months c. a.: antibiotic treatments (yes/no, number, number because of pulmonary infections), hospital admissions (yes/no, number, number because of pulmonary infections) will be compared by Chi-Square test or Wilcoxon tests.
12. Neurological assessment at 24 months c.a.: Results of Bayley III scale: Scale Language (sign, value), MDI ("Mental Development Index", sign, value), PDI ("Psychomotor Development Index") sign, value). Neurological diseases and their drugs and physiotherapy, Non-neurological diseases and their drug and therapy will be compared by Chi-Square test or Wilcoxon tests.
13. Anthropoemetric data: Weight, height, head circumference will be compared by Wilcoxon tests between treatment groups.

If data of study end could not be collected until the interim analysis and BPD, ROP, IVH, PVL, NEC (which should not be the case) and no BPD, ROP, IVH, PVL, NEC was collected, then the BPD, ROP, IVH, PVL, NEC was considered as not occurred for the patient.

6.10.2 Safety

6.10.2.1 Adverse Events

In the following analyses of the adverse events BPD, ROP, IVH, PVL and NEC are excluded.

Summary tables will present the number of subjects observed with AEs by MedDRA System Organ Class and Preferred Term and corresponding percentages. Additional subcategories will be based on event intensity and relationship to trial drug. A subject listing of all AEs will be prepared.

Medical terms will be coded by MedDRA terminology.
An overview table will display the absolute and relative number of patients with:

- At least one AE
- At least one AE with causal relationship
- At least one severe AE
- At least one related severe AE
- At least one SAE
- Death

Moreover, the absolute and relative frequencies will be displayed by preferred term within system organ class and treatment group.

6.10.2.2 Laboratory Parameters

Summary tables will be prepared to examine the changes of laboratory measures over time. Additionally, this will be visualized by Box-Whisker Plots over time.

6.10.2.3 Vital Parameters

Not applicable.

6.10.2.4 Pharmacokinetics

Not applicable.

6.10.3 Quality of Life

Not applicable.

6.11 Subgroup Analyses

For the following variables, subgroup analyses are planned:

- Trial site
- Birth weight < / ≥ 750 g
- Gender
- Antenatal steroid treatment
- Postnatal steroid treatment

Within these analyses, the primary analysis model will be extended by the respective subgroup variable and its interaction with the treatment effect, if the resulting analysis model is capable of properly estimating all effects in the model. Otherwise, separate analyses for all subgroups will be provided.

6.12 Interim Analysis

See section 2.2.

7 Software

All analyses will be performed by SAS, Version 9.4.
